# What does the literature mean by social prescribing? A critical review using discourse analysis

**DOI:** 10.1111/1467-9566.13468

**Published:** 2022-04-11

**Authors:** Sara Calderón‐Larrañaga, Trish Greenhalgh, Sarah Finer, Megan Clinch

**Affiliations:** ^1^ Centre for Primary Care and Mental Health Wolfson Institute of Population Health Queen Mary University of London London UK; ^2^ Bromley‐by‐Bow Health Partnership XX Place Health Centre Mile End Hospital London UK; ^3^ Nuffield Department of Primary Care Health Sciences University of Oxford Radcliffe Primary Care Building Radcliffe Observatory Quarter Oxford UK; ^4^ Barts Health NHS Trust Newham University Hospital London UK

**Keywords:** discourse analysis, literature review, primary health care, social prescribing

## Abstract

Social prescribing (SP) seeks to enhance the role of the voluntary and community sector in addressing patients' complex needs in primary care. Using discourse analysis, this review investigates how SP is framed in the scientific literature and explores its consequences for service delivery. Theory driven searches identified 89 academic articles and grey literature that included both qualitative and quantitative evidence. Across the literature three main discourses were identified. The first one emphasised increasing social inequalities behind escalating health problems and presented SP as a response to the social determinants of health. The second one problematised people's increasing use of health services and depicted SP as a means of enhancing self‐care. The third one stressed the dearth of human and relational dimensions in general practice and claimed that SP could restore personalised care. Discourses circulated unevenly in the scientific literature, conditioned by a wider political rationality which emphasised individual responsibility and framed SP as ‘solution’ to complex and contentious problems. Critically, this contributed to an oversimplification of the realities of the problems being addressed and the delivery of SP. We propose an alternative ‘care‐based’ framing of SP which prioritises (and evaluates) holistic, sustained and accessible practices within strengthened primary care systems.

## INTRODUCTION

Over the past decades, there has been an increasing interest in enhancing the role of the voluntary and community sector (VCS) in addressing patients' needs in primary care (Milbourne, 2009; Teasdale et al., 2012). Such efforts have mostly focussed on structuring intersectoral connections through the development of supported pathways for referring patients into the VCS. Social prescribing (SP) is one such example, whose most distinctive feature is the deployment of a new role, link workers or social prescribers, responsible for facilitating patients' journey from general practice to community‐based activities and organisations (Drinkwater et al., 2019). Link workers' role may range from signposting to more intensive approaches involving patients' needs assessments, ongoing support, coaching and motivational interviewing, or the development of new VCS activities where gaps exist (Brown et al., 2021). Community recommendations may be ‘lifestyle’ related (such as, cooking classes, exercise, or weight management schemes) or have a wider remit, including community engagement (volunteering, befriending) or welfare advice programmes (related to employment, housing or financial advice), depending on patients' needs and availability (Roland et al., 2020).

Social prescribing is growing internationally, with initiatives in United States, New Zealand, Australia, Spain and elsewhere (Aggar et al., 2020; Alderwick et al., 2018; Calderón‐Larrañaga & Braddick, 2021; Tava’e & Nosa, 2012). However, United Kingdom seems to be leading the way in establishing formalised, national SP pathways, with explicit mentions in subsequent policy reports, such as the NHS Five Year Forward View (NHS England, 2014), the General Practice Forward View (NHS England, 2016) and, more recently, the NHS Long‐Term Plan (NHS, 2019). By considering SP into its *“comprehensive model of personalised care”*, the NHS Long‐Term Plan marked a step change in ambition and set a target of recruiting enough link workers to make the service available in every NHS England GP practice by 2023/2024 (Hancock, 2018).

However, despite growing policy interest and proliferation, the evidence‐base for the effectiveness of SP interventions is still sparse and inconclusive. Quantitative studies and systematic reviews have often failed to prove consistent health, service utilisation or cost benefits (Bickerdike et al., 2017; Chatterjee et al., 2018; Gottlieb et al., 2017; Pavey et al., 2011; Pescheny et al., 2020; Public Health England, 2019), in part due to research methods and designs not best suited to evaluate such complex interventions. Qualitative studies and novel methodological approaches have enabled a better understanding of ‘why’, ‘for whom’, and ‘in what circumstances’ interventions might (or might not) work (Fixsen et al., 2020; Husk et al., 2020; Skivington et al., 2018; Tierney et al., 2020). Our previous realist review, for instance, critically explored what ‘good’ practice in SP looked like and how this could be best achieved, by identifying relevant individual, relational, organisational and policy resources (Calderón‐Larrañaga, Milner, et al., 2021).

Beyond uncertainty of whether and how SP works, there is also a need to explore how SP is being framed, conceptualised and ‘used’ in contemporary society and the scientific literature. Social prescribing programmes are developed and implemented within a wider social and cultural context where different (and often competing) interests, expectations and priorities co‐exist. As highlighted by the systematic review of Rempel et al. (2017), the intended aims of SP programmes often vary and might be different for different stakeholders: from cost savings, to resource reallocation or improved patients' mental, physical or social wellbeing. Questions, therefore, need to be asked about how such a complex set of claims and concerns become seemingly rational and coherent, as well as their potential impact on the way services are designed, implemented and evaluated.

In this study, undertaken as a background to an empirical realist evaluation on SP in populations at high risk of type 2 diabetes (Calderón‐Larrañaga, Clinch, et al., 2021), we sought to analyse how meaning and expectations around SP were constructed and reproduced in existing scientific literature. We adopted a critical and reflexive approach to the literature to identify recurring and conflicting discourses in evidence and theory. Our research questions were: ‘How is SP represented and understood in the scientific literature?’, ‘What are the implications of these different understandings for the development and implementation of SP?’ and ‘How do these understandings relate to larger overarching discourses within a broader socio‐historical and political context?’

## METHODS

### Epistemological position: Constructing meaning from discourse patterns

As Dryzek (1997) puts it, discourses can be understood as “*shared ways of apprehending the world*”. Each discourse rests on certain assumptions, judgements and claims that can be analysed in relation to specific social and historical contexts. Different social understandings of the world lead to different social actions. Discourse analysis is, therefore, not only interested in how meaning is constructed, but also in its wider social consequences (Yazdannik et al., 2017).

There is a wide range of approaches to discourse analysis, depending on the focus, sources of data or level of analysis (Glynos et al., 2009; Hodges et al., 2008). In this study, we drew on diverse discourse analytical approaches to explore the conceptual framings of SP in the scientific literature. Our study adopted a critical approach (in the sociological sense) to existing literature on SP that would go beyond a methods‐focussed critical appraisal. We sought to question the way in which the scientific literature frames its object of study (namely, SP) and the nature of the assumptions on which it draws. Following the classification of critique within the discourse‐historical approach proposed by Reisigl and Wodak (2009), we focussed on the contradictions, paradoxes and dilemmas in the text or discourse (immanent critique), while also revealing the underlying “*belief* (and knowledge) *systems*” in and by which these discourses operate (socio‐diagnostic critique).

All discourses are populated and constituted by elements of other texts, generating dynamic discourse systems linked across time and space (Conde, 2009). Within the SP literature, for instance, authors constantly quote and refer to previous texts in a dialogue that generates meaning (‘horizontal intertextuality’; Kristeva, 1991). Yet, as Fairclough (1992) emphasises, any given text is not only *built out of* texts from the past, but also transformed and emphasised in a manner which is socially and politically constrained. It is at this level that discourses come to be considered in light of broader ‘systems of knowledge’ or ‘ways of thinking’ (also referred to as ‘political rationalities’; Cornelissen, 2018). Our study sought to illuminate this *dialectical* relationship between discourses within particular scientific texts (‘micro‐’ or ‘little d’ discourse; Gee, 1999) and broader discursive patterns within a wider socio‐historical and political context (‘macro‐’ or ‘big D’ Discourse; Gee, 1999).

Discourse analysis is also concerned with the way in (and extent to) which certain behaviours or phenomena become a problem. The object of problematisation is, however, different across different discourse analysis approaches. In keeping with the discourse‐historic approach in critical discourse analysis, our review went beyond the identification of the contradictions and tensions within (and between) discourses, to also challenge the validity of these claims and their potential consequences (Glynos et al., 2009).

### Methodological approach

We applied critical discourse analysis to the studies included in a realist review on SP which we have published elsewhere (Calderón‐Larrañaga, Milner, et al., 2021). The search strategy combined a protocol driven database search with additional manual searches as per best practice recommendations for systematic reviews of complex evidence (Greenhalgh & Peacock, 2005).

Two distinct literature searches were carried out between September 2019 and May 2020 under the guidance of a specialist librarian. The strategy and databases for the *main search* are specified in Appendix 1. The main search and de‐duplication were reproduced by a second reviewer for consistency and discrepancies were solved by discussion. In addition to database searching, we manually retrieved citations contained in the reference list of relevant articles included in the review and searched for grey literature in websites of national charitable organisations related to SP. Based on the retrieved literature, policy‐level dimensions (including drivers and contractual agreements) were identified as in need of further exploration and refinement. In keeping with the iterative and theory driven nature of realist approaches to evidence synthesis (Pawson et al., 2004), *additional targeted searches* focussing on these specific domains were performed by manually retrieving articles from the reference list of relevant studies. The review included all studies published in English, French or Spanish on interventions linking adults (>18) in primary care with VCS organisations, regardless of study design (quantitative, qualitative and mixed methods) and including all SP related outcome measures. The relevance, rigour, and richness of all studies included were assessed (Appendix 2). We advise readers to access the review protocol (PROSPERO CRD42020196259) and article for further details (Calderón‐Larrañaga, Milner, et al., 2021).

Data analysis followed the guidelines provided by Willig (2008) and Potter and Wetherell (1987), supplemented by (macro‐level) features of Hajer's (2006) argumentative discourse analysis. It was conducted in five stages as specified in Table 1: reading, coding, analysis, validation and writing. In practice, these stages did not adopt a clear sequential order, but rather merged together in a dynamic and iterative process. We first read and reread all the studies during an initial familiarisation stage to gain an overview of the data and explore the construction and function of texts. Using the research questions as the basis for selection, we considered each article in its ‘wholeness’ and identified recurring and dominant themes across sections, which were coded and grouped together developing what Potter and Wetherell (1987) refer to as ‘bodies of instances’. At this stage, we followed an ‘inclusive’ approach that avoided setting limits to the data. All studies coded as containing relevant instances and the preliminary coding frame were then uploaded to Nvivo, which provided a platform to manage the organisation of data (see Appendix 3 for further detail on the coding frame and data extracts).

**TABLE 1:** Components of the discourse analysis in the critical literature review

Reading	Familiarisation with the topic area Underlining and marking of sections of texts with surprising, contradictory data Reading while ‘looking beyond the literal meanings of language’
Coding	Selection and organisation of data in preliminary ‘broad’ categories relevant to the research questions ‘Pragmatic’ (rather than an ‘analytic’) orientation ‘Inclusiveness’ during data selection (e.g., data which seemed only vaguely related to the research questions were also included)
Analysis	Identification of systematic patterns within the coded data in the form of both ‘variability’ (differences and contradictions in the content of accounts) and ‘consistency’ (similar features across accounts) Development of hypothesis about the functions of texts and the arguments being articulated and ‘pushed’ within (and across) discourses Identification of ‘discursive affinities’ across texts and broader systems
Validation	Analytic techniques for the validation of study findings included: Coherence: The capacity to explain how the discourse fits together and its identified effects and functions. Fruitfulness: The scope of our analytic scheme to facilitate understanding of new kinds of discourses and explain new phenomena. Investigator triangulation: Convergence of findings across different evaluators through ongoing discussion within the research team.
Writing	Ongoing clarification and development of the analysis and findings Detailed descriptions of data analysis and conclusions in order to allow the reader to assess and understand researchers' interpretations (e.g., we linked our analytic claims to specific parts and aspects of the data providing a representative set of examples)

*Source*: Adapted from other sources (Hajer, 2006; Potter & Wetherell, 1987; Willig, 2008).

Analysis involved careful reading and rereading of the coded data to identify relevant discursive patterns, both in terms of variation (differences and contradictions in the content of accounts) and consistency (similar features across accounts). We explored the potential function of texts, paying attention to the arguments being articulated and ‘pushed’ within (and across) discourses. We investigated the extent to which the identified discursive patterns embed, entail and presuppose other discourses, both in relation to previous texts and to broader systems of knowledge (‘discursive affinity’; Hajer, 2006). We validated our analysis iteratively by testing the *coherence* and *fruitfulness* of our findings, and through discussion within the research group. The process of writing helped clarify analytic issues and was therefore undertaken ongoingly. It involved writing down detailed explanations of the reasoning process and documenting our analytic claims and conclusions with specific examples and extracts from the data.

## RESULTS

### Overview of search results

**FIGURE 1:**
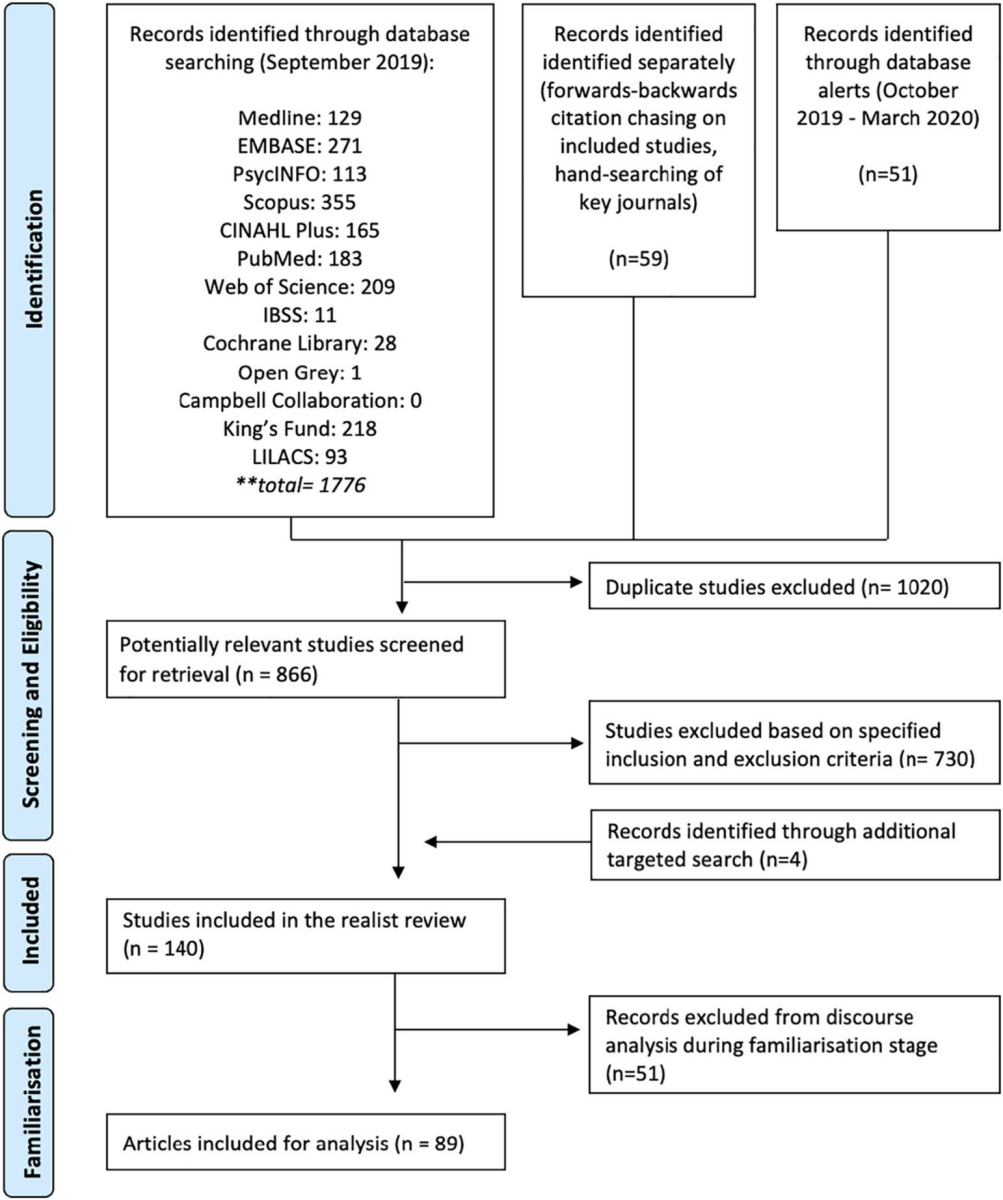
PRISMA flow diagram

Figure 1 illustrates the screening and selection process for our literature review. The above‐specified search strategy and inclusion criteria led to 140 studies. Following a familiarisation and coding stage, 89 references were included in the review for analysis. Of these, 28 were mixed‐methods studies, 26 used qualitative methods, 19 used quantitative methods, 15 were literature reviews and there was also an evaluability assessment study. 62 articles were published in peer‐reviewed journals, while the remaining 27 were publicly available reports produced by different academic companies and organisations (grey literature). Of our 89 texts, 83 were from UK, 4 from elsewhere in Europe, one from New Zealand and one from Australia. The characteristics of the studies included are further described in Appendix 4.

We identified three main discourses within the SP scientific literature (summarised in Table 2). Discourse 1 (*SP as helping to overcome the social determinants of health*) emphasised the influence of underlying social and structural factors on patients’ health outcomes and proposed SP as a response to the social determinants of health. Discourse 2 (*“From dependence to independence”: SP as supporting patients' journey towards self‐activation*) depicted SP as a means (‘temporary’, ‘limited’) of supporting patients become ‘independent’ and reducing their reliance on overstretched health and social services. Discourse 3 (*SP as enhancing personalised care in general practice*) presented SP as shared, open‐ended and personalised care practices, capable of restoring person‐centredness in primary care. Discourses were distributed unevenly across different type of studies and article sections within the literature reviewed (see Appendix 3 for further detail). Although each discourse made distinct arguments and claims, they shared a tendency towards framing SP in terms of ‘solution’ delivered though individual patient care to address complex and contentious social and health system ‘problems’.

**TABLE 2:** Summary of different discourses in the social prescribing scientific literature

	Discourse 1. SP as helping to overcome the social determinants of health	Discourse 2. SP as supporting patients' journey towards self‐activation	Discourse 3. SP as enhancing personalised care in general practice
What is the rationale for SP?	Growing health and social inequalities	Growing demand and use of healthcare resources	Declining human and relational dimensions in general practice
What is the main aim of SP?	To address the social determinants of health	To reduce health service utilisation	To provide personalised, empathetic care
What does SP look like?	A referral pathway to community‐based services (related to employment, welfare advice, housing, etc.)	Coaching, activation, motivational interventions, time‐bound	Ongoing, dynamic, shared, open‐ended care networks and relationships
What arguments and claims are being made?	That SP will contribute to redress health and social inequalities by addressing the social determinants of health	That SP will contribute to reduce health service utilisation and ease pressure on the system by enhancing self‐care	That SP will contribute to restore person‐centeredness in general practice by providing personalised, un‐hurried and empathetic care
Assumed characterisation of general practice	Biomedical, clinical, at risk of ‘over‐medicalising’ patients' ‘social’ dimensions	Overstretched, overused, unsustainable	Impersonal, instrumental, fragmented, devoid of affective or socio‐emotional components
Assumed characterisation of SP users	Individuals with social needs (social isolation, unemployment, housing problems)	Individuals with ‘capacity’ to choose and overcome problems (‘clients’)	Individuals with enduring and complex health needs (‘patients’)
What is considered to be of value?	Service model, organisational rearrangements	Efficiency, cost‐effectiveness	Human dimensions, relationships, experiences, reciprocities
Distribution within papers	Introductory sections, to define the rationale and potential of SP	Methods and results sections, to design, measure, interpret the potential of SP	Qualitative verbatims within results sections, to understand the reality of patients and providers involved
Typical research design	Epidemiological, population‐based, observational. Emphasis on describing social and health inequalities	Randomised controlled trial (hypothesis‐driven, deductive), emphasis on size, scale and generalisability	Ethnography, in‐depth interview, focus group (qualitative, inductive), emphasis on understanding individuals' lived experience

### Discourse 1. Social prescribing as helping to overcome the social determinants of health

Social prescribing interventions were often framed within a broader body of literature that emphasised the influence of wider “*social, economic and cultural factors*” on health outcomes (Bungay & Clift, 2010). ‘Unhealthy’ behaviours and subsequent higher risk of disease among study populations were often explained in terms of unequal distribution of opportunities and socio‐economic disadvantage. Health and community sectors were identified as key actors in addressing social inequalities, which meant that the response was often framed in terms of health and social service provision (e.g., SP, community‐centred approaches to health): *“Evidence that people's education, income, housing and other social issues have a major impact on their health and wellbeing is well established. Given this important relationship, there is growing international interest in the role of healthcare systems in addressing patients' social (i.e. non‐medical) needs”* (Pescheny et al., 2020).

References to the social determinants of health were also present when characterising access and healthcare usage patterns in primary care. Growing pressures in general practice were explained in terms of the increasing number of patients contacting a healthcare professional for *“non‐medical”* reasons (Ferguson & Hogarth, 2018). This time, however, ‘social’ and ‘medical’ dimensions were not depicted as mutually determined (e.g., adverse social circumstances lead to poor health and greater healthcare need), but rather as separate (even dichotomised) reasons for consultation (*“20% of people attend GP surgeries for social problems”*; Coan, 2016): *“GPs spend nearly a fifth of their consultation time dealing with non‐medical issues at a cost of £395 million* per annum*, equivalent to the salaries of 3750 full‐time GPs. Almost three‐quarters of GPs state that the proportion of time they spend dealing with non‐health issues as part of consultations has increased”* (Ferguson & Hogarth, 2018).

Social prescribing users were depicted as individuals facing mainly “*social problems*”, such as *“social isolation, loneliness, housing issues or bereavement”* (Pescheny et al., 2020). General practice was presented as unable to address adequately these ‘non‐medical’ concerns (for instance, as not having enough time to acknowledge patients' social circumstances, at risk of over‐medicalising patients' illnesses) and, consequently, in need of a structural change. Social prescribing was then framed as a means to addressing both existing failures of the health system and patients' wider social determinants of health:

*… [name of the SP scheme] illustrates how social prescribing can offer the opportunity to address social needs through individual consultations. An added bonus may be the reduction of workload and more capacity to focus on medical problems. […] A claim can be made that social prescribing, through addressing the wider determinants of health, represents a reorientation of health services […]* (South et al., 2008).

References to the social determinants of health were mainly present in the introductory sections of the articles when defining the rationale and potential of SP interventions. Few studies acknowledged the value of link workers' advice (and support) for problems related to housing, employment or welfare benefits referencing patients' experience in the results section (Moffatt et al., 2017; Pescheny et al., 2020). In the remaining cases, the impact of SP interventions on socio‐economic dimensions was either not measured (mostly) or not demonstrated (Aggar et al., 2020).

### Discourse 2. “From dependence to independence”: Social prescribing as supporting patients' journey towards self‐activation

Within this discourse, SP was contextualised in a socio‐sanitary reality characterised by people's increasing use of and reliance on public services that were depicted, consequently, as being overstretched. Social prescribing was then presented as an alternative potentially capable of enhancing patients' capacity to self‐manage and reducing their reliance on health and social services: *“In the UK, an ageing population combined with a growing number of people living with long term medical conditions is increasing demand and cost pressures on the acute, primary and social care services […] A key demand has been for services to become more integrated to better serve the complex needs of the older, frail population and to be more focussed on encouraging supported self‐management, as a means to reduce demand on primary and secondary care services, making them more sustainable”* (Elston et al., 2019).

As outlined by the title of the Rotherham SP evaluation report, “*From dependence to independence*” (Dayson et al., 2013), patients were meant to overcome a status of ‘dependency’ (also referred to as ‘lack of control’, ‘vulnerability’) and move towards a state of ‘self‐efficacy’ (or ‘independence’, ‘activation’) with the help of appropriate techniques and community‐based interventions: “*The [SP programme] endeavours to signpost and provide the person with the information and support they require in order to help them to remain independent in their own homes for as long as possible and reduce their future reliance on health and social services.”* (Beech et al., 2017)

Lack of ‘self‐perception’, ‘motivation’ or ‘confidence’ was considered a barrier for successful ‘engagement’ and ‘behavioural change’. Interventions, therefore, comprised and prioritised “*coaching*” and “*motivational*” strategies for achieving intended outcomes (Husk et al., 2020). Training of link workers (also referred to as “*wellbeing coaches*” (Heijnders & Meijs, 2018)) often involved motivational interviewing or goal‐setting techniques (Wildman, Moffatt, Penn, et al., 2019).

Within this discourse, ‘independence’ was equated with self‐management and reduced utilisation of services, whereas ‘dependency’ was deemed problematic. Being (or becoming) ‘too’ reliant on others did not only need to be overcome (potentially with SP), but could also represent a threat to SP implementation and delivery: *“There is a danger of patients becoming dependent on a link worker as the source of support; this should be tempered if individuals create new and meaningful connections within the community, which may include reconnecting with friends and family because of a more positive outlook on life. Such an improved outlook may encourage those with existing health conditions to actively engage in self‐care.”* (Tierney et al., 2020)

Social prescribing schemes, consequently, developed different ‘boundary setting’ strategies to prevent or address ‘dependency’. Certain schemes offered a limited pre‐established number of appointments (*“up to three appointments of approximately up to 40 min each”*; South et al., 2008) to discuss patients' needs and identify relevant community‐based resources. Further approaches identified by Wildman, Moffatt, Penn, et al. (2019) included *“regularly reminding clients of the limits of the link worker role, creating distance by doubling‐up, swapping link workers or running group activities and reasserting the importance of empowerment rather than dependency”*. Support (or care) was conceptualised either as a menu from which referred patients were encouraged to ‘choose’ or as a means (‘temporary’, ‘limited’) of helping patients become free from further needing it.

Both behavioural and social determinants of health were often framed as matters that could be addressed through individual action. The focus was placed on the individual (as opposed to on the structural constrains) and their capacity to ‘engage’ with (as opposed to ‘access’) the ‘prescribed’ advice, support and/or activities. This discourse reframed the ‘solution’ in terms of inner rearrangements (e.g., “change in attitude”, “raise of expectations”, “self‐confidence”, “re‐activation”; Beech et al., 2017; Bertotti et al., 2018), assuming that a rational decision‐making and behavioural change would follow: “*Improvements in confidence, self‐esteem, independence, and motivation enabled clients not only to set new goals, but also to actively pursue them”* (Payne et al., 2020).

Constrains were also often depicted as belonging to the private or personal sphere (of their *“own”*; Bertotti et al., 2018), rather than structural and hence shared by those in similar socio‐economic positions. ‘Empowerment’ was equated with patients' capacity to take *“ownership of their problems”* (Faulkner, 2004), successfully overcome them, and lessen any reliance on health services: *“Socially orientated approaches delivered through [SP] may broaden community capacity and empower patients to better manage their own health and make more appropriate use of health services”* (Southby & Gamsu, 2018). This discourse reinforced the idea of ‘positive change’ (‘advancement’, ‘improvement’, ‘progress’) as a single endeavour by placing the responsibility in the individual: *“while a link worker could ‘encourage and support’, long‐term change was about ‘taking responsibility for yourself…nobody else is going to do it’”* (Wildman, Moffatt, Steer, et al., 2019).

The way authors measured and made sense of their study outcomes was heavily influenced by this discourse. Researchers often drew on theoretical references and frameworks that emphasised individual agency, resilience, and self‐efficacy. Morton et al. (2008), for instance, sought to investigate whether an exercise on prescription scheme could foster self‐determined motivation and subsequent behavioural change. Hanlon et al. (2019) explored whether *Self Determination Theory* could be used to understand the change (or lack thereof) in behaviour and wellbeing resulting from patients' involvement in a Links Worker Programme. Tierney et al. (2020) drew on *Patient Activation Theory* to conceptualise and analyse the role of link workers in SP interventions. Other studies drew on *Salutogenesis Model* (Beech et al., 2017; Jensen, 2019) and its focus on *“people's resources, capabilities and the mechanisms that create and sustain health*” (Swift, 2017). Bertotti et al. (2018) referenced self‐efficacy within *Social Cognitive Theory* to explain behavioural change when evaluating the conditions and mechanisms that facilitated (or hampered) the implementation of a SP intervention. The *Social Cure* perspective was also used to explain how social group membership developed within a SP programme enhanced participants' confidence building and wellbeing (Kellezi et al., 2019).

Interventions were evaluated (and deemed successful) based on their potential to enhance participants' self‐concept, self‐management and/or behavioural change. Mental wellbeing questionnaires (such as, the Warwick‐Edinburgh Mental Wellbeing Scale), which involved the assessment of participants' self‐perception (e.g., confidence in themselves), were widely used amongst studies included in our sample. Physical health and behavioural questionnaires were also often employed. Many primary and secondary studies chose social and healthcare service utilisation indicators, including primary care attendance, secondary care referrals and/or contacts with community‐based NHS services and Accident and Emergency as outcome variables to monitor effectiveness (see Appendices 3 and 4 for further detail). Different SP interventions identified in our literature review targeted patients who frequently visited their GP or other primary care providers. Loftus et al. (2017), for instance, focussed on patients over 65 with long term conditions who attended their GP frequently or had multiple medications. Brandling and House (2007) evaluated a SP programme aimed at patients defined as “high resource users”. In all cases, researchers assessed the capacity of the intervention to reduce service utilisation or primary care workload, generally through the enhancement of self‐management or ‘activation’ strategies.

### Discourse 3. Social prescribing as enhancing personalised care in general practice

This third discourse emphasised the dearth of human and relational dimensions within general practice. Clinical appointments were depicted as ‘rushed’, ‘hurried’, ‘impersonal’ and hence unable to accommodate patients' needs and expectations. Within this context, clinicians were often characterised as unable and/or unwilling to explore and listen to patients' wider psychosocial concerns, leading to ‘judgemental’, ‘prescriptive’ and ‘un‐empathetic’ encounters: *“I am stuck in this wheelchair and have a lot of problems. I knew that my GP just wanted to get rid of me out of the door. I knew she didn't want to open up the can of worms that were in my head and forcing me to talk to the Samaritans”* (Kimberlee, 2016).

Social prescribing was then framed as an alternative capable of counteracting these relational shortfalls, by removing ‘time bound’ appointments and providing a holistic, caring and personalised service. Time and space for “*feeling listened to and valued*” (Pescheny et al., 2018) were considered key programme components and preconditions for ‘good’ practice: *“I knew what was going on in my head, but I couldn't always, I didn't always want to tell anyone. It seemed, with the link‐worker, it seemed as though I could get over that more quickly. He wasn't demanding. He was very quiet and very gentle with it, and* that is *the way that I needed somebody to be, to maybe listen to me, really listen to me, and hear what I was saying […]”* (Kellezi et al., 2019).

Rather than a provisional transaction, SP was conceptualised as an ongoing ‘practice’ which required perseverance and attentiveness. Care was understood as a ‘need’, rather than a ‘choice’ (“I will always need somebody to help me”; Wildman, Moffatt, Steer, et al., 2019), refined and reinvented dynamically over time depending on its results and patients' fluctuating needs: *“[…] another [link worker] suggested that it takes time to develop relationships because of people's complex problems […]: ‘it took time, you know, to build up that relationship with the individual, but you can see just the difference it's made, you know, he knows I'm there and you know I guess it's like chiselling away, each time that I see him, you know, he'll tell me something else’”* (Mercer, 2017).

Social prescribing was no longer articulated as a linear referral pathway towards a predefined destination, but as a care network comprising different actors. Patients moved back and forth across settings and sectors depending on their changing needs, which required ongoing and bidirectional coordination between care providers. A caring and supportive SP was deemed necessary to ensure successful outcomes. Patients, for instance, were more likely to participate when link workers contacted them directly after receiving the referral, made regular follow up phone calls, or even came along with them to the planned activities. Emotional and practical support seemed to allow patients to overcome (or cope with) the barriers that often prevented them from engaging: “*I just expected the Link Worker to introduce me to the gym, and that would have been it. And I think, if it had just been [that] I would have turned round, and I would have gone the opposite direction. But because of the way it was so gradually and really professionally linked into different things, I just felt as though I'd floated into it, rather than getting shoved from behind. I just felt as though I was gradually moved into it*” (Moffatt et al., 2017).

Support and encouragement not only prevented dropouts, but also enabled people to push themselves harder than they would have by themselves. Similarly, patients were more likely to progress when feeling committed to a regular service provided (“*If you build up a relationship with somebody like Mary you're not going to let her down*”*;* Stirrat, 2014). Yet, care was not only considered a means towards ‘engagement’, but also an outcome in itself. Knowing that support was available, as well as feeling listened to and cared for were sufficient and relevant endpoints (“*it is very comforting to know that you are not by yourself, that you can ring someone*”; Beech et al., 2017). Social prescribing users were depicted as ‘patients’ (as opposed to ‘clients’) facing enduring and complex health issues and hence in need of continuous and open‐ended care for when things went wrong again: “*I mean with me, I'd still want to be in contact somewhere along the line, which I think they will do. If something happened to me, […] I think I would need them full time all the time then*” (Wildman, Moffatt, Steer, et al., 2019).

As the verbatims above reveal, this discourse mostly drew on patients', link workers' and community stakeholders' lived experience and accounts gathered through qualitative interviewing. Questions related to the provision of enhanced, ongoing care were not, however, addressed explicitly in study aims nor informed by relevant theoretical references. Within the discussion sections, findings were either included as recommendations for improved SP delivery (Faulkner, 2004; Husk et al., 2020), problematised in the context of an overstretched primary care system (Mercer, 2017) or treated as ‘unintended’ (and hence to be prevented) for potentially implying an increased patients' reliance on health services and running counter initial expectations (Wormald et al., 2006).

## DISCUSSION

This study, based on argumentative discourse analysis of 89 references, identified three main ways of understating the scope and potential of SP interventions. As summarised in Table 2, discourses differed in their rationale, claims and the characterisation of SP and social reality. Discourses circulated unevenly across different type of studies and article sections within the literature reviewed. While discourse 1 was mainly present as a rationale for SP, discourse 2 was consistently used to design, measure, and interpret existing interventions. Discourse 3 was mostly stressed by participants in qualitative studies and often criticised by study authors. We also identified a shared tendency across discourses, whereby SP initiatives were consistently framed in terms of ‘solutions’ to complex and contentious problems. The extent to which this SP discursive landscape is shaped (and reinforced) by a wider political rationality and the consequences of these alignments are discussed below.

### Tackling structural inequalities through health service innovation

Our first discourse exposed a tension whereby SP interventions tended to acknowledge structural injustice but then offered health service innovations and individualised strategies as ‘solutions’ for them. This critical distance between a starting upstream claim and an ultimate downstream denouement has already been acknowledged in the scientific literature (referred to as *“lifestyle drift”* [Popay et al., 2010; Williams & Fullagar, 2019] or “*neoliberal justice narratives”* [Littler, 2018]). Our study highlights that this ‘drift’ often happens through a process which enhances the role and responsibilities of individuals, health services and communities. We argue this may prove problematic on the following basis.

Growing health inequalities in the UK (and globally) are highly conditioned by underlying structural inequalities (Bambra & Garthwaite, 2015; Karanikolos et al., 2013; Stuckler et al., 2017). Maldistribution of power, wealth and resources operate through a wide range of social and economic pathways (including employment, income, housing and education) to generate unequal health outcomes (McCartney et al., 2020). As pointed out by the WHO Commission on Social Determinants of Health, individual and community‐level interventions, such as SP, are well‐placed to ‘reduce the consequences’ of such inequalities through the provision of enhanced care and support. However, they fail to tackle the system which generates (and reproduces) maldistribution, for which system‐level interventions would prove more appropriate (Commission on Social Determinants of Health, 2008). Presenting SP as ‘the solution’ may hamper a broader understanding of and response to the social determinants of health, which also addresses its fundamental structural causes and asserts policy‐level responsibilities (Baum & Fisher, 2014; Gibson et al., 2021).

Discourse 1 also depicted social determinants of health as definite (even computable) reasons for consultation in general practice, easily detachable from the more ‘medical’ ones. However, health and social dimensions tend to form a continuum, be mutually determined and appear intertwined in consultations (Heath, 1995). As pointed out by Stange and Ferrer (2009) the acknowledgement and understanding of these inter‐relations have proven to be a precondition for the provision of personalised, high quality clinical care in general practice. Presenting SP as a strategy capable of addressing the ‘non‐medical’ needs, may risk exacerbating this contrived dichotomy (‘social’ vs. ‘medical’) while eroding primary care clinicians' responsibility to explore, understand and integrate patients' wider social needs and circumstances in routine consultations (also referred to as ‘*holistic SP*’ and considered best practice (Calderón‐Larrañaga, Milner, et al., 2021).

### Easing pressure on the system through the enhancement of self‐care

Our review identified a dominant discourse around patients' ‘independence’, which depicted SP as a means of enhancing their capacity to self‐manage and easing pressure on the system. These expectations seem to have *solidified* in specific institutional arrangements. NHS England and Improvement, for instance, encourages the use of the Patient Activation Measure tool to assess the “*knowledge, skills and confidence of a person to manage their own health and care*”, as a proxy for reduced service utilisation when evaluating SP programmes (NHS England, 2018). The embeddedness of this discourse into specific institutional and organisational practices (also referred to as “*discourse institutionalisation”*; Hajer, 2006) highlights its consistency and dominance across the SP arena. We argue this may be problematic on the following basis.

This discourse assumedly linked self‐management with ‘independence’ and reduced reliance on further care. Yet, as highlighted by Hinder and Greenhalgh (2012), self‐management is rarely an individual, isolated endeavour. Rather, it is often enabled (or constrained) by economic, material and socio‐cultural conditions within the family, community and health services (Hinder & Greenhalgh, 2012). Shifting the work of (‘self’‐) care away from clinic risks placing additional demands and burdens on ‘informal’ care providers (family and community), raising ethical and sustainability issues (especially where sufficient or strengthened material and relational resources are not ensured; May et al., 2014). Voluntary and community sector organisations and local authorities operating in deprived communities, for instance, have reported an increased demand for services as a result of patients' underlying socio‐economic circumstances, along with ongoing funding deficits, which affect the sustainability and capacity of their services (NAO, 2018; NCVO, 2015).

Critically, the notion of a capable, self‐sufficient and independent individual might prove unrealistic for some patients, and lead to significant frustration and guilt when unattained (Peacock et al., 2014). For some patients and in certain circumstances, accepting personal boundaries (“relinquishing control” or “letting go”) and the need for help is beneficial and empowering (Aujoulat et al., 2008). Besides, there are cases where trustful and personalised relationships with link workers and/or the VCS made patients feel safe to disclose problems which often required further clinical input (Tierney et al., 2020). In such cases, patients were referred ‘back’ to general practice, enhancing access to (and utilisation of) health services. ‘Good’ practice in SP may not necessarily involve reduced service utilisation. A SP whose main aim is to ease pressure on the system risks overshadowing (and hence not strengthening) relations of interdependence, collaboration and mutual responsibility, which are relevant endpoints to patients and predict ‘good’ practice in SP (Calderón‐Larrañaga, Milner, et al., 2021).

Lastly, pressures in general practice have resulted from an increasing workload over the last decades, without a matched growth in either funding or workforce (Baird et al., 2016). While work has become more complex and intense in the UK general practice, funding for primary care as a share of the NHS overall budget has gradually fallen (Baird et al., 2016). Framing SP (and the enhancement of self‐care) as the ‘solution’ to overstretched health services, may hinder the consideration and tackling of system factors and supply‐side deficiencies which highly contribute to explain increasing pressures in primary care.

### Restoring person‐centredness in general practice through social prescribing

Discourse 3 depicted patient‐clinician interactions within general practice as overtly instrumental (oriented to preventing, diagnosing or treating disease – ‘cure’ talk) and devoid of any type of affective or socio‐emotional component (‘care’ talk) (Greenhalgh & Heath, 2010). Social prescribing was then presented as a strategy capable of restoring this imbalance, by creating a new role (link workers) in charge of providing a caring, person‐cantered, empathetic approach.

This discursive reality is reinforced by a general practice where the reason for consultation, rather than the relationship with the patient, shapes the organisation of service provision (Rudebeck, 2019). Triaging and task distribution have gradually replaced relationship continuity of care (understood as *“the relationship between a single practitioner and a patient that extends beyond specific episodes of illness or disease* *”*; Haggerty, 2003) despite being associated with better clinical outcomes and reduced all‐cause mortality (presumably in relation to improved clinical responsibility, physician knowledge, and patient trust; Baker et al., 2020). Clinicians are increasingly meant to deal with diseases efficiently, while all the rest (including relationship competence; Rudebeck, 2019) is no longer recognised as a vital professional asset and may therefore be shifted to other members of staff (usually less specialised and resourced; Nancarrow & Borthwick, 2005). Objective and definable processes (‘cure’ talk) are more easily monitored, owned and regulated than the numerous intangibles in routine consultations (‘care’ talk), which are more prone to be overlooked and transferred (Nancarrow & Borthwick, 2005). However, both ‘talks’ are required in order for clinical care to reach its full potential. Framing SP as a ‘solution’ risks disregarding existing trends, their consequences, and the need to ensure therapeutic relationships across disciplinary boundaries (including in general practice).

### Rethinking social prescribing beyond a ‘solutionist’ paradigm

As Peter Miller's and Nikolas Rose's (1990) work revealed, there tends to be a reciprocal interaction between language (*‘linguistic features’*) and wider systems of knowledge, where discourses act as means through which (and in which) specific political rationalities are reproduced, consolidate and influence human action (‘*govern’*). A wider neoliberal rationality resonates with a (dominant) understanding of SP which focused on patients' knowledge and resilience (via informed discussions with link workers and motivational coaching) as a means of consolidating positive lifestyle choices and reducing their reliance on further care. Similarly, a shared “*solutionist*” (Morozov, 2013) approach to SP also contributed to enhance the role and responsibilities of individuals, communities and health services in tackling structural and contentious problems (such as, social inequalities, overstretched health services, or increasing fragmentation in general practice). While end users and providers were expected to invest themselves with new skills and ‘ways of doing’ (Brown & Baker, 2013), the context of possibilities and constrains in which these actions may (or may not) happen was frequently overshadowed (Mackenzie et al., 2020; Scott‐Samuel & Smith, 2015).

The relationship between discourses, practices and wider political rationalities is, however, far from linear. As Brown (2015) points out, even when one political rationality becomes hegemonic, it carves itself against a range of other possibilities – “*tacitly arguing with them, keeping them at bay, or subordinating them*”. There are different ways of understanding and practicing SP which challenge (while co‐exist with) neoliberalism. Our realist review and ongoing realist evaluation, for instance, identified SP practices which contributed to enhance GPs' understanding of patients' wider needs and their capacity to provide ‘holistic’ and accessible care. Link workers and community organisations provided sustained, open‐ended care to better respond to patients' enduring and complex needs. Critically, this often involved going beyond what was expected, or even disregarding and questioning the way in which services had been designed and commissioned (Calderón‐Larrañaga, Milner, et al., 2021).

These examples allow for the configuration of an alternative ‘care‐based’ framing of SP, which sees the provision of holistic, sustained and accessible primary care not so much as a means to an end, but as an end in itself (Heath, 2021; Mol, 2008). We speculate that these dissenting (and inspiring) practices contribute to enact an alternative ‘belief system’ whose main rationale is meeting patients' primary care needs through publicly accountable and collaborative services (of which SP would constitute an example). This conceptualisation of SP necessarily shifts the attention of research from *measuring* impact (via service utilisation indicators) to *evaluating* the extent to which SP may (or may not) succeed to support people in greatest need while contributing to stronger, fairer health care systems.

### Strength and limitations

To our knowledge, this is the first study employing a discourse analysis approach to SP. Diverse and relevant theoretical references allowed us to explore the meanings and expectations around SP in the scientific literature, while highlighting the conditions of possibility and legitimacy for certain discourses to become dominant. Using a critical approach, our review unravelled existing tensions and taken‐for‐granted assumptions, and problematised what these assumptions meant and entailed for the implementation and delivery of SP.

The main limitation of this study is its reliance on a predefined literature search (as opposed to an iterative literature search strategy). As specified in the methods section, we applied a critical discourse analysis approach to the references included in our previous realist review on SP. However, the exhaustive realist review search strategy (which combined searches in 13 databases and additional manual searches, leading to the inclusion of 140 studies) proved sufficiently comprehensive to allow for the description and validation of the identified discursive axes.

## CONCLUSIONS

The way in which SP is framed and conceptualised in the scientific literature influences its implementation and evaluation. Our review identified three main ways of understanding SP and unravelled overlaps between them. Discourse 1 emphasised increasing social inequalities behind escalating health problems, while presenting SP as a response to the social determinants of health. Discourse 2 problematised people's increasing use of health and social services and depicted SP as a means of enhancing self‐management and reducing patients' reliance on further care. Discourse 3 stressed the dearth of human and relational dimension in general practice, while presenting SP as an alternative capable of restoring person‐centeredness. Discourses circulated unevenly in the scientific literature, conditioned by a wider political rationality which emphasised individual responsibility and framed SP in terms of ‘solution’ to complex and contentious problems. We speculate that this contributed to oversimplify both the realities and problems being addressed and constrain the way interventions are delivered. Critically, once the “*solutionist*” narrative is exposed as a cover‐story, a range of different narratives and evaluative frameworks become possible. We conclude that these alternative framings broaden our political imagination to rethink (and enhance) the scope and possibilities of SP interventions within stronger and fairer primary health care systems.

## CONFLICT OF INTEREST

The authors declare that no competing interests exist.

## ETHICS STATEMENT

This project has been approved by the Office for Research Ethics Committees Northern Ireland (reference: 20/LO/0713).

## AUTHOR CONTRIBUTIONS

**Sara Calderón‐Larrañaga**: Conceptualisation (lead); Data curation (lead); Formal analysis (lead); Funding acquisition (lead); Investigation (lead); Methodology (equal); Project administration (lead); Writing – original draft (lead). **Trish Greenhalgh**: Conceptualisation (supporting); Formal analysis (supporting); Funding acquisition (supporting); Investigation (supporting); Methodology (equal); Supervision (equal); Writing – review and editing (equal). **Sarah Finer**: Conceptualisation (supporting); Formal analysis (supporting); Funding acquisition (supporting); Investigation (supporting); Methodology (equal); Supervision (equal); Writing – review and editing (equal). **Megan Clinch**: Conceptualisation (supporting); Formal analysis (supporting); Funding acquisition (supporting); Investigation (supporting); Methodology (equal); Supervision (equal); Writing – review and editing (equal).

## Supporting information

Supporting Information 1

Supporting Information 2

Supporting Information 3

Supporting Information 4

## Data Availability

The data that supports the findings of this study are available in the supplementary material of this article.
