# Supplementary material for: What does the literature mean by social prescribing? A critical review using discourse analysis
Source: Sociol Health Illn. 2022 Apr 11;44(4-5):848–68. doi: 10.1111/1467-9566.13468 (PMC9321825; doi:10.1111/1467-9566.13468)
Supplement: Supplementary file 1 — Supporting Information 1 [file SHIL-44-848-s003.docx]

**Appendix 1. Literature searches**

1. **Literature searches**

The final search strategy included combinations of search terms for the concepts ‘social prescribing’, ‘primary care’, ‘community referral’ and was informed by previous related reviews on SP and further developed in ﻿MEDLINE (Ovid) using an iterative process of adding, removing and refining search terms.

In September 2019, the following databases were searched: MEDLINE (Ovid), EMBASE (Ovid), PsycINFO (Ovid), Scopus (Elsevier), Web of Science (Clarivate Analytics), CINAHL Plus (EBSCO), PubMed (NCBI), International Bibliography of the Social Sciences - IBSS (ProQuest), ﻿The Cochrane Database of Systematic Reviews (The Cochrane Library), Campbell Collaboration, Open Grey (INISR-CNRS), King’s Fund Database and LILACS (BIREME).

Database alerts were set up to identify studies published between October 2019 and March 2020.

**Database: MEDLINE**

Host: Ovid.

Date searched: 27 September 2019.

Searcher: Sara Calderón, Yasmin Milner.

Hits: 129.

Strategy:

1. (social prescri* and type 2 diabetes).mp.
2. (social prescri* or community referral).mp.
3. (social prescri* or community referral or non medical referral).mp.
4. (social prescri* and primary health care).mp.
5. (social prescri* and primary care).mp.
6. (social prescri* or community referral or referral scheme).mp.
7. ((((social prescri* or community referral) and primary care) or primary health care) and diabetes).mp.
8. ((social prescri* or community referral) and primary health care and diabetes).mp.
9. 1 or 2 or 3 or 4 or 5 or 8

**Database: EMBASE**

Host: Ovid.

Date searched: 27 September 2019.

Searcher: Sara Calderón, Yasmin Milner.

Hits: 271.

Strategy:

1. (social prescri* and type 2 diabetes).mp.
2. (social prescri* or community referral).mp.
3. (social prescri* or community referral or non medical referral).mp.
4. (social prescri* and primary health care).mp.
5. (social prescri* and primary care).mp.
6. (social prescri* or community referral or referral scheme).mp.
7. ((((social prescri* or community referral) and primary care) or primary health care) and diabetes).mp.
8. ((social prescri* or community referral) and primary health care and diabetes).mp.
9. 1 or 2 or 3 or 4 or 5 or 8

**Database: PsycINFO**

Host: Ovid.

Date searched: 27 September 2019.

Searcher: Sara Calderón, Yasmin Milner.

Hits: 113.

Strategy:

1. (social prescri* and type 2 diabetes).mp.
2. (social prescri* or community referral).mp.
3. (social prescri* or community referral or non medical referral).mp.
4. (social prescri* and primary health care).mp.
5. (social prescri* and primary care).mp.
6. (social prescri* or community referral or referral scheme).mp.
7. ((((social prescri* or community referral) and primary care) or primary health care) and diabetes).mp.
8. ((social prescri* or community referral) and primary health care and diabetes).mp.
9. 1 or 2 or 3 or 4 or 5 or 8

**Database: Scopus**

Host: Elsevier.

Date searched: 29 September 2019.

Searcher: Sara Calderón, Yasmin Milner.

Hits: 355.

Strategy:

1. TITLE-ABS-KEY ("Social Prescri*" OR "community referral" AND "type 2 diabetes")
2. TITLE-ABS-KEY ("Social Prescri*" OR "community referral")
3. TITLE-ABS-KEY ("Social Prescri*" OR "community referral" AND "diabetes" AND "primary health care")
4. TITLE-ABS-KEY ("Social Prescri*" OR "community referral" AND "primary health care")
5. TITLE-ABS-KEY ("Social Prescri*" AND "primary health care")

|  |
| --- |

**Database: CINAHL Plus**

Host: EBSCO.

Date searched: 30 September 2019.

Searcher: Sara Calderón, Yasmin Milner.

Hits: 165.

Strategy:

1. "Social prescri*" OR "community referral"

**Database: PubMed**

Host: NCBI.

Date searched: 30 September 2019.

Searcher: Sara Calderón, Yasmin Milner.

Hits: 183.

Strategy:

1. (((((((social prescri*) OR "community referral")) AND "primary health care")) OR ((social prescri*) OR "community referral"))) OR ((((social prescri*) OR "community referral")) AND diabetes)
2. (((((social prescri*) OR "community referral")) AND "primary health care")) OR ((social prescri*) OR "community referral")

**Database: Web of Science**

Host: Clarivate Analytics.

Date searched: 30 September 2019.

Searcher: Sara Calderón, Yasmin Milner.

Hits: 183.

Strategy:

1. ("social prescri*")
2. ("social prescri*" OR "community referral")
3. ("social prescri*" OR "community referral") *AND* (diabetes)
4. ("social prescri*" OR "community referral") *AND* ("primary health care")
5. #4 OR #3 OR #2 OR #1

**Database: IBSS**

Host: ProQuest.

Date searched: 30 September 2019.

Searcher: Sara Calderón, Yasmin Milner.

Hits: 11.

Strategy:

1. noft("community referral") OR noft("social prescri*")

**Database: Cochrane Library**

Host: Cochrane Collaboration.

Date searched: 30 September 2019.

Searcher: Sara Calderón, Yasmin Milner.

Hits: 28.

Strategy:

1. "social prescri*" in Title Abstract Keyword OR "community referral" in Title Abstract Keyword AND "primary care" in Title Abstract Keyword
2. "social prescri*" in Title Abstract Keyword OR "community referral" in Title Abstract Keyword AND diabetes in Title Abstract Keyword
3. "social prescri*" in Title Abstract Keyword OR "community referral" in Title Abstract Keyword

**Database: Open Grey**

Host: INISR-CNRS.

Date searched: 30 September 2019.

Searcher: Sara Calderón, Yasmin Milner.

Hits: 1.

Strategy:

1. "social prescribing" OR "community referral"

**Database: LILACS**

Host: BIREME.

Date searched: 30 September 2019.

Searcher: Sara Calderón, Yasmin Milner.

Hits: 93.

Strategy:

1. (tw:("community referral"))
2. (tw:("community referral")) AND (tw:("social prescribing"))

**Database: King’s Fund**

Host: The King’s Fund.

Date searched: 3 October 2019.

Searcher: Sara Calderón, Yasmin Milner.

Hits: 218.

Strategy:

1. "social prescribing"
2. "community referral" AND "social prescribing"
