# Supplementary material for: What does the literature mean by social prescribing? A critical review using discourse analysis
Source: Sociol Health Illn. 2022 Apr 11;44(4-5):848–68. doi: 10.1111/1467-9566.13468 (PMC9321825; doi:10.1111/1467-9566.13468)
Supplement: Supplementary file 2 — Supporting Information 2 [file SHIL-44-848-s004.docx]

**Appendix 2. Quality appraisal criteria**

1. **Relevance**

*Main search:*

- High relevance: studies that evaluated an intervention comprising a comprehensive assessment of patients’ circumstances and needs by a link worker or social prescriber.
- Low relevance: studies evaluating an intervention that did not comprise an assessment by a link worker or social prescriber and where the activities ‘prescribed’ in general practice were pre-defined from the outset, such us, Arts of Prescription or Exercise on Referral Schemes.

*Additional search:*

- High relevance: studies that focused on the organizational and policy environment within which SP interventions are commissioned and delivered.
- Low relevance: studies that did not focus on the organizational and policy environment within which SP interventions are commissioned and delivered.

1. **Richness,** based on the criteria proposed by Ritzer (1) and Roen et al. (2):

- Conceptually rich: studies with well-grounded and clearly described theories and concepts.
- Conceptually thick: studies with rich description of a programme was provided, but without explicit reference to the theory underpinning it.
- Conceptually thin: studies with weak programme description where discerning theory would have been problematic.

1. **Rigour**

- Critical Appraisal Skills Programme (CASP) for qualitative studies, systematic reviews, randomised controlled trials (RCT), case control studies, cohort studies and economic evaluations.
- National Institutes of Health (NIH) quality assessment tool for before-and-after studies with no control group and cross-sectional studies
- Mixed Methods Appraisal Tool (MMAT) for mixed methods studies

REFERENCES:

1. Ritzer G. Meta-theorizing in Sociology. Lexington Books, editor. Lexington, MA; 1991.

2. Roen K, Arai L, Roberts H, Popay J. Extending systematic reviews to include evidence on implementation: Methodological work on a review of community-based initiatives to prevent injuries. Soc Sci Med [Internet]. 2006 Aug;63(4):1060–71. Available from: https://linkinghub.elsevier.com/retrieve/pii/S0277953606000943
