# Supplementary material for: What does the literature mean by social prescribing? A critical review using discourse analysis
Source: Sociol Health Illn. 2022 Apr 11;44(4-5):848–68. doi: 10.1111/1467-9566.13468 (PMC9321825; doi:10.1111/1467-9566.13468)
Supplement: Supplementary file 3 — Supporting Information 3 [file SHIL-44-848-s001.docx]

**Appendix 3. Coding frame, data extract, article sections coded**

Appendix 3 provides further detail on the coding frame, data extracts and the article sections coded. The article section for each data extract is provided at the end of the quote and highlighted in different colours (introduction/background in red; methods section in green; results section in blue; and discussion in yellow).

**Discourse 1. SP as helping to overcome the social determinants of health**

Code 1.1. Aim and rationale related to SDH and health inequalities

Code 1.2. SP as able to address non-medical needs (characterisation of SP)

Code 1.3. Social vs medical types of demands (characterisation of general practice)

Code 1.4. Patients as facing mainly “social” problems (characterisation of SP users)

Code 1.5. Empirical references to the impact of SP on socio-economic domains

**Discourse 2. SP as supporting patients’ journey towards self-activation**

Code 2.1. Rationale related to health care utilisation

Code 2.2. SP aimed at reducing health service utilisation

Code 2.3. SP aimed at enhancing self-care, activation, independence, control, motivation, personal responsibility

Code 2.4. Intervention characteristics: coaching/motivational approach, lifestyle-related, time-bound, led by coaches trained in motivational techniques

Code 2.5. Characterisation of SP users: at risk of being/becoming dependent, lacking control, lacking motivation, frequent attenders

Code 2.6. Evaluation approaches in line with Discourse 2

- Code 2.6.1. Theoretical frameworks
- Code 2.6.2. Outcomes related to self-concept, self-management, behavioural change, independence, activation
- Code 2.6.3. Outcomes related to social and healthcare service utilisation

Code 2.7. Lifestyle drift – references to “choice”, “empowerment”

**Discourse 3. SP as enhancing personalised care in general practice**

Code 3.1. Impersonal, rushed, stretched general practice (characterisation of general practice)

Code 3.2. SP as able to deliver empathetic, person-cantered care (characterisation of SP)

Code 3.3. Impact of empathetic, supportive, person-cantered care on adherence, behavioural change, satisfaction

Code 3.4. Patients as individuals with enduring and complex health issues in need on ongoing care (characterisation of SP users)

**Discourse 1. SP as helping to overcome the social determinants of health**

**Code 1.1. Aim and rationale related to SDH and health inequalities**

*[…] Given the impact on practice, it would seem logical that primary care plays a part in addressing the wider determinants. (1) – Results section*

*[…] Until more recently, preventative health initiatives have focussed on “downstream” lifestyle interventions in spite of the strength of evidence challenging such approaches (Scott-Samuel and Smith, 2015). However, the publication of Sir Michael Marmot’s “Fair Society, Healthy Lives” Report in 2010 has strengthened the call to focus on the* ***“causes of the causes”*** *of poor health which he and others attribute to the “social inequalities in the conditions in which people are born, grow, live, work and age” (Marmot, 2010). Consequently, it has been argued that efforts to improve the nation’s health must also consider the social determinants of health and this has spawned a growing interest in community-centred health approaches (Rippon and Hopkins, 2015; Finnis et al., 2016). (2) - Introduction*

*It has long been recognized that health is influenced by a broad range of social, economic and cultural factors, and indeed the current political emphasis appears to be on the wider community and the sociocultural factors that may impact on health and well-being. This is a more* ***holistic*** *approach to care which takes account of wider social factors impacting on the individual and their specific illness or condition. (3)* *- Introduction*

*As an influential factor, the Marmot Review (Marmot, 2010) highlighted the social deter- minants of health inequity and although it did not refer overtly to social prescribing, it recommended the creation and development of sustainable communities, and strengthening the role and the impact of ill health prevention; key areas that social prescribing seeks to address. (4) - Introduction*

*As demonstrated by the Marmot Review on health inequalities, there is a close association between socio-economic factors and health outcomes. Other research has shown that in England, GPs spend nearly a fifth of their consultation time dealing with non-medical issues at a cost of £395 million per annum, equivalent to the salaries of 3,750 full-time GPs. Almost three-quarters of GPs state that the proportion of time they spend dealing with non-health issues as part of consultations has increased. (5) - Introduction*

*Health inequalities are a global problem, resulting from a fundamental inequity in the distribution of income, power, and wealth. This inequity limits opportunities across the life course, including In addition, people of low socioeconomic status experience multiple health problems and a concentration of risk factors,3 exacerbated by poor access to resources to manage them.4 (6) – Introduction*

*Academic research demonstrates that social well-being is closely tied to physical health, a well-known example being the impact of socioeconomic positioning on mortality as demonstrated in the Whitehall Studies, as well as other more recent work by Michael Marmot.2 3 Though this common understanding has not fully translated into clinical practice and public health. (7) – Introduction*

*Lifestyle and the physical, economic and social environment are among factors that determine individual health,4 and access to and use of healthcare services has less of an impact,5 accounting for approximately 10% of population health and well-being.6 UK GPs report that 20% of consultations are related to patient psychosocial needs. (8) – Introduction*

*Creative Alternatives operates as part of a network of social prescribing programmes within Sefton that also includes Active Sefton (physical activity), Relax and Revive (physical activity, including yoga and tai chi), Active Reading (bibliotherapy) and the Citizens Advice Health Outreach scheme. These programmes recognise the importance of social, economic and cultural factors on well‐being […] (9) – Introduction*

*Mental illness is affected by biological, psychological, and social factors, and treatments targeting a range of these factors, including the wider determinants of health, are more likely to be effective (WHO 2005). Social prescribing programs address these biopsychosocial factors via care coordination and linkage where individuals with mental illness are referred to local community-based social care services and structured social activities (Knapp et al. 2012). (10) – Introduction*

*The Marmot Review highlighted the value of providing social prescribing services, presenting a direct correlation between health inequalities and social, environmental and economic factors (Marmot et al., 2010), and promotes social prescribing as a model to address the wider health determinants (Wilson, 2015). (11) – Introduction*

*Central to our proposed theory of change was a recognition that health problems are caused or exacerbated by social problems (Marmot, 2010), and so we felt it was important to offer social solutions to respond to such challenges that would complement medical treatments. In respect of this, we reviewed the available literature looking at community wellbeing interventions and chose to focus on three core offers: community navigation, social prescribing and social action (South, 2015). (2) – Methods*

**Code 1.2. SP as able to address non-medical needs (characterisation of SP)**

*The potential for primary care to ‘rediscover’ a ‘social’ model of practice (Olesen et al., 2000) and contribute to addressing social determinants of health (British Medical Association (BMA), 2011) and health inequalities (Marmot, 2008), respectively, is gaining increasing policy prominence. (12) - Introduction*

*Living Well, Taking Control acts at a local institutional or community level. In this sense the programme is one part of a wider group of agencies, programmes and social practices that may impact on diabetes prevention and management. The role of the programme in contributing to a partnership and strategic agenda can be understood through policy and social change theory such as the Socio-Ecological Model (Glanz et al., 2008) (13) - Introduction*

*Examples of psychosocial problems are social isolation, loneliness, housing issues, bereavement and poor wellbeing. However, it is clear that the relationship between psychosocial factors and health is bidirectional. Evidence that people’s education, income, housing and other social issues have a major impact on their health and wellbeing is well established.3 Given this important relationship, there is growing international interest in the role of healthcare systems in addressing patients’ social (i.e. non-medical) needs.(14) – Introduction*

*Supporting people whose health problems are exacerbated or created by complex socioeconomic factors is a challenge to healthcare systems, especially for GPs and other primary care staff.1 Patients in these contexts can also be dissatisfied with the support they receive.2–6 However, GP practices in the UK are in a unique position, providing universal coverage free at the point of care in the community as part of the NHS, allowing continuity and trusting relationships to be fostered. They, therefore, provide an ideal setting to implement interventions with the potential to mitigate the impact of the social determinants of health,7 necessary for primary health care.8,9 (15) - Introduction*

*In essence, the underlying model of care needs to change to a biopsychosocial model, considering, in addition to biological factors and medical interventions, the wider determinants of health and coordination and integration of care across professionals and disciplines.(16) – Introduction*

*The Links Worker Programme (LWP) is a social prescribing initiative in areas of high deprivation in Glasgow, Scotland, that is designed to mitigate the negative impacts of the social determinants of health. […] GP practices in the UK […] provide an ideal setting to implement interventions with the potential to mitigate the impact of the social determinants of health,7 necessary for primary health care. (15) – Introduction*

*The CHAT scheme illustrates how social prescribing can offer the opportunity to address social needs through individual consultations. An added bonus may be the reduction of workload and more capacity to focus on medical problems. […] A claim can be made that social prescribing, through addressing the wider determinants of health, represents a reorientation of health services […] (1) – Introduction*

*Purpose – A community-centred approach to health called Community Wellbeing Practices (CWP) is being offered to patients at all 17 GP practices in Halton in order to respond more appropriately to patients’ social needs, which are often an underlying reason for their presentation at primary care services. (2) – Introduction*

*Social prescribing interventions benefit patients by supporting them to address the wider psychosocial determinants of health, enabling better health‐condition management and the adoption of healthier behaviours (Mossabir, Morris, Kennedy, Blickem, & Rogers, 2015) (17) – Introduction*

*‘Holistic’ social prescribing interventions seek to address the wider social determinants of health and, therefore, go beyond the neoliberal standpoint of viewing individual health behaviours as the personal failings […] (18) – Introduction*

*In the last few years there has been an emergence of interventions focusing on the social component of care, such as social prescribing, art on prescription, exercise/physical activity on prescription, walking groups and the introduction of health trainers, with some evidence for behaviour change [2–4]. These aim to help people manage their chronic condition, prevent more serious health problems developing, and contribute to addressing health inequalities […] (19) – Introduction*

*Mental illness is affected by biological, psychological, and social factors, and treatments targeting a range of these factors, including the wider determinants of health, are more likely to be effective (WHO 2005). Social prescribing programs address these biopsychosocial factors via care coordination and linkage where individuals with mental illness are referred to local community-based social care services and structured social activities (Knapp et al. 2012). (10) – Introduction*

*Therefore, a tool to increase social contact and promote community support and engagement within deprived populations has potential to address some of these factors and hopefully reduce the impact of social deprivation. (20) - Discussion*

*The Marmot Review highlighted the value of providing social prescribing services, presenting a direct correlation between health inequalities and social, environmental and economic factors (Marmot et al., 2010), and promotes social prescribing as a model to address the wider health determinants (Wilson, 2015). (11) – Introduction*

*Social prescribing is viewed as a means of addressing mental, psychosocial, or socioeconomic issues, and enhancing community well-being and social inclusion (Scottish Development Centre for Mental Health, 2007). As such, it is an emerging strategy for tackling health inequities through partnerships between primary care and third sector organisations (4) – Introduction*

*Here, the PSS was set up to act as a voluntary advisory/referral agency for patients whose problems were perceived to stem from underlying psychosocial issues. It was argued that these patients would beneﬁt from a support network that would help them to explore their problems, advise them and, where necessary, refer them to appropriate community-based services. Consequently, it was envisaged that a patient’s psychosocial state may be prevented from deteriorating by attending to and hopefully resolving the root cause(s) of their problems, e.g. relationship problems, ﬁnancial difﬁculties, housing problems. (21) - Methods*

*Recognising that an individual’s health is primarily determined by a range of social, economic and environmental factors, social prescribing strives to address the individual’s need in a holistic way. (22) – Introduction*

*Social prescribing can therefore strengthen the links between health care providers and community, voluntary and local authority services. In these services there are potential solutions to the wider determinants of mental health, for example, leisure, welfare,*

*education, culture, employment and the environment (Scottish Development Centre for Mental Health, 2003:5). (23) - Results*

*Objectives: Social prescribing has emerged as a useful tool for helping patients overcome some of the social and behavioural determinants of poor health (24) - Introduction*

*A common policy response to health inequalities in recent years has been the introduction of various social prescribing programs. Social prescribing aims to link patients to nonmedical sources of support within a community, thus expanding options and resources beyond those traditionally provided in primary health care (6) - Introduction*

*The Links Worker Programme (LWP) is a social prescribing initiative in areas of high deprivation in Glasgow, Scotland, that is designed to mitigate the negative impacts of the social determinants of health. […] Greater collaboration between the healthcare sector and community-based provision of health and social care, with involvement of community, voluntary, and third-sector organisations (hereafter referred to as community organisations), has been promoted to help mitigate the effects of the social determinants of health.10,11 One such model is social prescribing, which encompasses a range of approaches. […] These forms of social prescribing provide the potential for primary care teams to respond more effectively to the social determinants of health and to widen the support network accessible to people presenting at their GP practice.18 This approach could also potentially ameliorate some of the effects of the inverse care law operating in deprived areas.6,19,20 (15) - Introduction*

*A claim can be made that social prescribing, through addressing the wider determinants of health, represents a reorientation of health services, albeit one limited in scale and scope. (1) C2 – Results*

*Purpose – A community-centred approach to health called Community Wellbeing Practices (CWP) is being offered to patients at all 17 GP practices in Halton in order to respond more appropriately to patients’ social needs, which are often an underlying reason for their presentation at primary care services. The paper aims to discuss these issues. (2) – Background/purpose*

*In recognition of these wider determinants of wellbeing, social prescribing has become a core component of current and future National Health Service (NHS) policy and practice to deliver person-centred care [3] and reduce GP workload [4]. […] Consequently, the United Kingdom (UK) has been at the forefront of formalising the use of social prescribing alongside traditional medical treatment within primary care, to address the environmental, economic, social and psychological issues affecting people’s well-being (25) - Background*

*Social prescribing is underpinned by a social model of health, which recognises that common mental health problems are often psychological manifestations of social problems. Non-medical interventions are thought to offer social and individual solutions by increasing social contact and improving access to services (South et al. 2008). (26) – Introduction*

*Central to our proposed theory of change was a recognition that health problems are caused or exacerbated by social problems (Marmot, 2010), and so we felt it was important to offer social solutions to respond to such challenges that would complement medical treatments. In respect of this, we reviewed the available literature looking at community wellbeing interventions and chose to focus on three core offers: community navigation, social prescribing and social action (South, 2015). (2) – Methods*

*﻿In brief, the participatory arts have been widely used in the promotion of health for many years, and the social value of engagement with the arts has been endorsed by the World Health Organization,5 who call for partnerships between health and other sectors to address social and economic problems – Introduction (27)*

*﻿Social prescribing interventions have ranged from physical exercise (e.g. exercise referral, green gyms) to personal study (e.g. books on prescription, education on prescription) and creative activities (e.g. arts on prescription including dance, film, music and painting). Schemes that have sought to address the social determinants of health include information prescriptions (e.g. debt advice, housing, welfare); healthy living initiatives (e.g. smoking cessation, healthy eating, health checks); social enterprise ﻿schemes or social firms (e.g. community businesses, co-operatives, credit unions); and time banks, which are mutual volunteering schemes where people deposit time helping others and withdraw time when they need help. (28) - Introduction*

It runs from April 2012 to March 2014 as part of a wider GP-led Integrated Case Management Pilot and aims to increase the capacity of GP practices to meet the **non-clinical needs** of their patients with long term conditions (LTCs)(29)

**Code 1.3. Social vs medical types of demands (characterisation of general practice)**

*Primary care in the United Kingdom currently faces a number of key challenges including: (i) About 20% of people attend GP surgeries for problems that are primarily social rather than medical (Torjesen, 2016). (ii) A rising tide of long-term conditions which is set to grow by 5 million in the next 10 years (DoH, 2013). (iii) Growing health inequalities which result in long-term medical conditions disproportionately affecting people in deprived areas (Hutt and Gilmour, 2010; Marmot et al., 2010; Cawston, 2011).* (30) *- Introduction*

*It is estimated that around 20% of patients consult their health care professional (HCP) for what is primarily a social problem. A large proportion of health outcomes, estimated at 70%, are the result of social and economic determinants of health including employment, financial security, housing, diet and exercise, familial and social networks*(31) *- Introduction*

*Up to one in five cases seen by a general practitioner (GP) are for difficulties that could be classed as ‘non-medical’ (e.g. inadequate housing, financial issues, bereavement, loneliness) (25) - Introduction*

*Lifestyle and the physical, economic and social environment are among factors that determine individual health,4 and access to and use of healthcare services has less of an impact,5 accounting for approximately 10% of population health and well-being.6 UK GPs report that 20% of consultations are related to patient psychosocial needs. (8) – Introduction*

*Social prescribing interventions are often targeted at people in socioeconomically deprived areas, expanding options available to primary-care practitioners when patients present with needs related to wider social determinants of health [8]. Social, rather than health, problems place considerable burdens on primary care, with 20% of patients consulting their general practitioner (GP) for primarily social problems and 15% of patients visiting for welfare-benefits advice [9]. A common criticism of public health interventions is their tendency to focus on individual-level health behaviours and overlook the structural determinants of health [10]. (18) – Background*

*As 20% of patients may consult their general practitioners (GPs) for a social problem,2 social prescribing has attracted increasing interest among GPs, social scientists, allied health professionals, the voluntary sector and commissioning stakeholders. (24) – Background*

*As demonstrated by the Marmot Review on health inequalities, there is a close association between socio-economic factors and health outcomes. Other research has shown that in England, GPs spend nearly a fifth of their consultation time dealing with non-medical issues at a cost of £395 million per annum, equivalent to the salaries of 3,750 full-time GPs. Almost three-quarters of GPs state that the proportion of time they spend dealing with non-health issues as part of consultations has increased. (5) - Introduction*

**Code 1.4. Patients as facing mainly “social” problems (characterisation of SP users)**

*Examples of psychosocial problems are social isolation, loneliness, housing issues, bereavement and poor wellbeing. However, it is clear that the relationship between psychosocial factors and health is bidirectional. Evidence that people’s education, income, housing and other social issues have a major impact on their health and wellbeing is well established.3 Given this important relationship, there is growing international interest in the role of healthcare systems in addressing patients’ social (i.e. non-medical) needs.*(14) *– Introduction*

*Purpose – A community-centred approach to health called Community Wellbeing Practices (CWP) is being offered to patients at all 17 GP practices in Halton in order to respond more appropriately to patients’ social needs, which are often an underlying reason for their presentation at primary care services.* (2) *– Introduction*

*Here, the PSS was set up to act as a voluntary advisory/referral agency for patients whose problems were perceived to stem from underlying psychosocial issues. It was argued that these patients would beneﬁt from a support network that would help them to explore their problems, advise them and, where necessary, refer them to appropriate community-based services. Consequently, it was envisaged that a patient’s psychosocial state may be prevented from deteriorating by attending to and hopefully resolving the root cause(s) of their problems, e.g. relationship problems, ﬁnancial difﬁculties, housing problems.* (21) *- Methods*

*Recognising that an individual’s health is primarily determined by a range of social, economic and environmental factors, social prescribing strives to address the individual’s need in a holistic way.* (22) *– Introduction*

*Social prescribing interventions are often targeted at people in socioeconomically deprived areas, expanding options available to primary-care practitioners when patients present with needs related to wider social determinants of health [8]. Social, rather than health, problems place considerable burdens on primary care, with 20% of patients consulting their general practitioner (GP) for primarily social problems and 15% of patients visiting for welfare-benefits advice [9]. A common criticism of public health interventions is their tendency to focus on individual-level health behaviours and overlook the structural determinants of health [10].* (18) *– Background*

*As demonstrated by the Marmot Review on health inequalities,i there is a close association between socio-economic factors and health outcomes. Other research has shownii that in England, GPs spend nearly a fifth of their consultation time dealing with non-medical issues at a cost of £395 million per annum, equivalent to the salaries of 3,750 full-time GPs. Almost three-quarters of GPs state that the proportion of time they spend dealing with non-health issues as part of consultations has increased.* (5) *– Introduction*

**Code 1.5. Empirical references to the impact of SP on socio-economic domains**

*where possible, took practical action to maximise income, reduce debt and assist service users navigating the welfare benefits system: 'Whatever money I owed like electricity and TV licence was in my mind always eating me from inside. I sorted out that and it just changed so many things … It changed my attitude, it changed my behaviour and it changed my mood … I am not depressed like before … I feel better about everything … I go out almost every day … I play more, I write, I do jobs at home … I read more … yes, a big change.' (P30, female, 45–49 years) 'Because of my language barrier some things I could not sort things out so easily, but she could. Even though I didn’t know about Attendance Allowance she applied for that … I didn’t know which benefit I could get. She said, ‘I can try this one.’ She tried it and she was successful and it helped me a lot.' (P16, female, 65–69 years) For those of working age, health-related unemployment was a major problem. Steps to assist with finding paid or volunteer work, returning people to work or having reasonable adjustments to work settings were undertaken and highly appreciated: 'I felt 100% better after talking to [Link Worker], she was just excellent … She put us in contact with people [to] do a new CV and look for a new job, [and] found information out for us … which has given me the confidence to go back to my company … things were put in place at work, so that I could take my breaks when I needed them … It made a massive difference to me, personally … I was getting on board with [mental health condition], and now, I’m at work with renewed vigour.' (P9, male, 55–59* (32) *– results*

*To address wider economic and social issues, navigators provided advice and information, or made referrals into services to address debt, welfare, employment and housing issues.14,32,37 Numerous qualitative studies found that SP helped service users to find a job or to go back to work after illness or accident, through employment assistance (e.g. curriculum vitae writing), motivation, and support from navigators and referred services. […] Another important aspect of SP services was to help service users to understand their situation and to access a range of welfare benefits they are entitled to but often not aware of.14,16,37 Moreover, navigators took practical action to solve daily problems and worries of service users.16 Service users reported that due to SP they felt able to better cope with day-to-day activities* (14) *– results*

**Discourse 2. SP as supporting patients’ journey towards self-activation**

**Code 2.1. Rationale related to health care utilisation**

*The United Kingdom’s (UK’s) National Health Service (NHS) faces increasing pressure on its resources […] Consequently, voluntary organisations and charities as third sector organisations are increasing their role in providing an adjunct to primary care services (Coid, Williams, & Crombie, 2003; Secretary of State for Health, 2006)(4) – introduction*

*There are 15 million people with long-term conditions, and over two million with multiple long-term conditions (Department of Health 2012). As the population ages, this figure is likely to grow. This population accounts for 55 per cent of GPs appointments and 77 per cent of inpatient bed days (House of Commons Health Committee 2014).*(33) *C2– introduction*

*In the UK, an ageing population combined with a growing number of people living with long-term medical conditions is increasing demand and cost pressures on the acute, primary and social care services (Wanless et al., 2006; Licchetta and Stelmach, 2016). […] A key demand has been for services to become more integrated to better serve the complex needs of the older, frail population and to be more focused on encouraging supported self-management, as a means to reduce demand on primary and secondary care services, making them more sustainable (Dyson, 2014; NHS England, 2014, 2016a, 2016b).*(34) *– introduction*

*The pressures faced by General Practice are highlighted by the growing complexity of managing multiple long-term conditions33 and greater collaboration between health professionals is needed to improve outcomes for patients.* (35) *– discussion*

*“Now what’s happening is within the NHS, the stress is so much on ‘Don’t get people entrenched within the services, move them on, find them some mainstream opportunities which are part of the community rather than keep them within your day services, within the NHS’, so certainly, there is more scope for partnership working in the future, I would say.” (R9)* (36) *- results*

**Code 2.2. SP aimed at reducing health service utilisation**

*The objectives of the social prescribing pilot in City and Hackney included (City and Hackney CCG, 2013): ∙ Enable individuals to* ***feel more in control*** *and improve health and well-being ∙ Reduce social isolation ∙ Increase GP awareness of what is happening in the community and vice versa ∙ Reduce GP visits and A&E attendance* (30) *– introduction*

*The WBC endeavours to signpost and provide the person with the information and support they require in order* ***to help them to remain independent in their own homes for as long as possible and reduce their future reliance on health and social services****.*(37) *– introduction*

*[…] social prescribing may represent a way to* ***manage increasing demands on both the NHS and GPs’ time*** *(Buck, 2016).(11) – introduction*

*As many countries grapple with developing* ***cost-efficient methods*** *to manage LTCs and tackle the gap between the social and healthcare sector, SP offers a potential* ***solution*** *and ﻿our findings present some of the opportunities and challenges in its implementation and evaluation. (14) – discussion*

*It was assumed that SP would not only improve patients’ mental well-being and* ***‘activate’ them to better self-manage their health****, but would also lead to reduced demand on primary and acute health care and social care services.* (34) *– introduction*

*The primary hypothesis was that the intervention would improve health and social wellbeing, patient activation and frailty levels, and that this would lead to less use of primary, social and acute care services and reduced costs.* (34) *– introduction*

*The aims are to increase patients’ illness* ***self-management****, address their psychosocial and health needs, and through this to reduce primary healthcare usage.* (38) *– introduction*

*Surgeries may also beneﬁt through a reduction in GP attendance, the result of patients gaining support from alternative sources.(21) – introduction*

*These approaches, often labelled ‘social prescribing’, can range from financial advice to walking groups and enable healthcare providers to respond to a broad range of patient needs, as well as potentially reducing GP and emergency department service demand (Polley, Bertotti, Kimberlee, Pilkington, & Refsum, 2017).*(39) *– introduction*

*Socially orientated approaches delivered through GPVCS collaborations may broaden community capacity (Friedli et al., 2012) and empower patients to better manage their own health and make more appropriate use of health services.*(40) *– introduction*

*The Department of Health (HM Government, 2006) has advocated social prescriptions for almost ten years whilst more recently NHS England (2014) has promoted non-clinical interventions from the voluntary and community* ***sector as a way of making general practice more sustainable***(41) *- introduction*

*﻿Similarly, the welsh NHS Confederation found that the ‘range of ﻿social prescribing projects and initiatives have the potential to make real progress towards improving population health and well-being and reducing demand on NHS wales’ (p. 1). (28) – introduction*

**Code 2.3. SP aimed at enhancing self-care, activation, independence, control, motivation, personal responsibility**

*Strength and responsibility […] Even though the researchers did not bring them*

*up during the interviews, subjects such as the participant’s own strength and responsibility were frequently mentioned. The term ‘own strength’ refers to the power to ﬁnd one’s own solutions to problems.* (42) *– results*

*Evaluation of Living Well, Taking Control A community-based diabetes prevention and management programme (13) – title*

*‘a means of enabling GPs and other frontline healthcare professionals to refer patients to a link worker – to provide them with a face-to-face conversation during which they can learn about the possibilities and* ***design their own personalised solutions****, i.e. “co-produce” their “social prescription”– so that people with social, emotional or practical needs are* ***empowered to ﬁnd solutions*** *which will improve their health and wellbeing, often using services provided by the voluntary and community sector’*(30) *– introduction*

*Scaled-up versions of individual social prescribing initiatives could be used to counter the social determinants of health inequity, in offering* ***purposeful activities that build*** ***resilience*** *in the face of mental and physical ill health, encourage social interaction, self-esteem and* ***confidence****, and develop individual and community resources. (4) - background*

*Information derived from EA indicated that CWS was conceived as a programme to reduce health inequalities by improving* ***awareness*** *of factors influencing mental and physical health, increasing uptake of support services and (consequently) enhancing* ***self‐efficacy, coping skills, empowerment and*** ***confidence*** (43)  *– results*

*People’s health is determined primarily by a range of social, economic or environmental factors and social prescribing seeks to address people’s needs in a holistic way, allowing them to take greater control of their own health.* (44) *– introduction*

*It has emerged as a tool to help clinicians to persuade suitable patients to* ***engage*** *in a new and helpful change in behaviour.(24) - introduction*

*one-to-one signposting and individual support were expected to result in patients being more able to acquire and use available skills, information and support which was expected to lead, in turn, to patients better self-management of health conditions, better ability to navigate systems, avert crises and adapt to challenges, which was also expected to lead to improved relationships with professionals and a sense of being valued as a ‘whole person’. All of these together were expected to lead to improved wellbeing* (45) *- methods*

*Participants use this action strategy* ***to manage the challenges/barriers*** *that may obstruct success. When faced with problems such as low levels of confidence or environmental barriers, the action strategy may be to avoid and drop out of the scheme. The dimension of the strategy allows a gradient of responses. For example, the participant may only attend the minimum required of the scheme protocol when faced with a barrier. In practice, this could result in a participant attending the initial appointment, reassessments, and the minimum of exercise sessions. However, if the participant has* ***sufficient levels of confidence and motivation*** *they can respond with attendance to all that the scheme has to offer for them; Hanna: ‘‘I find this is my level now I can come to aqua twice a week I find I am comfortable with this’’ (FG4, 260). The consequences of the scheme are shown (Figure 1 and Table 1) as the ‘‘physical and psychological outcomes,’’ alongside ‘‘scheme development,’’ such as the expansion and refinement of the schemes protocols and objectives.* (46) *- results*

*shows that those likely to benefit from seeing a link worker are patients able to change their outlook on life, who can build and sustain their social capital. This may only happen when motivation and engagement are present.(25) - results*

*The objectives of the social prescribing pilot in City and Hackney included (City and Hackney CCG, 2013): ∙ Enable individuals to* ***feel more in control*** *and improve health and well-being ∙ Reduce social isolation ∙ Increase GP awareness of what is happening in the community and vice versa ∙ Reduce GP visits and A&E attendance* (30) *- introduction*

*However, it has subsequently become recognised that social prescribing could provide new opportunities and a chance for* ***patients to take responsibility*** *and be more creative (Branding and House, 2009b) whilst also increasing self–esteem and providing social support to both individuals and communities.* (47) *C 2.3. – Introduction*

*The PSS may also be seen as empowering patients to* ***take control*** *over their lives. For example, a recent factor analysis of empowerment in healthcare outlines components of care that are similar to those provided by the PSS [14], such as providing patients with information and* ***choices*** *concerning their future care. The concept of Empowerment has been empirically linked to* ***learned mastery*** *theory [15] whereby patients who are given control over their lives become increasingly* ***motivated****. (21) – discussion*

*The overall hypothesis underpinning the realist evaluation of social prescribing in City and Hackney is that social prescribing improves well-being outcomes for patients suffering from isolation, and mild mental health problems. It provides a mechanism of support that enables each individual participant to consider a set of actions they may be willing to take, and thus embark on the journey to socially re-activate themselves, change their behaviour and, ultimately, their health.* (30) *– results*

*From a more conceptual point of view, when the relationship between SPC and patient develops successfully, the patient develops a strong sense of self-efﬁcacy, feeling of control and a willingness to take on and persist with new and difﬁcult tasks (Coulter and Ellins, 2006). The motivation and support offered by SPCs creates the basis for behaviour change such as greater willingness to participate in chosen community activities.* (30) *– discussion*

*Creative Alternatives operates as part of a network of social prescribing programmes within Sefton that also includes Active Sefton (physical activity), Relax and Revive (physical activity, including yoga and tai chi), Active Reading (bibliotherapy) and the Citizens Advice Health Outreach scheme. These programmes recognise the importance of social, economic and cultural factors on well‐being and provide Sefton residents with a ‘menu’ of treatment options that encourage levels of self‐help, personal responsibility and social and community engagement. (9) – introduction*

*Social prescribing interventions benefit patients by* ***supporting them*** *to address the wider psychosocial determinants of health, enabling better health‐condition management and the adoption of healthier behaviours (Mossabir, Morris, Kennedy, Blickem, & Rogers, 2015) (17) – introduction*

*In the last few years there has been an emergence of interventions focusing on the social component of care, such as social prescribing, art on prescription, exercise/physical activity on prescription, walking groups and the introduction of health trainers, with some evidence for behaviour change [2–4].* ***These aim to help people manage their chronic condition****, prevent more serious health problems developing, and contribute to addressing health inequalities by building social support networks. (19) – background*

*Social prescribing programs can be broadly focussed, for example the Expert Patients Program, where participants attended self-management groups to improve self-efficacy, confidence, and QoL (Rogers et al. 2008). (10) – introduction*

*The NHS ‘Five year Forward View’ (NHS 2014) calls for the development of new and innovative approaches to help people living with long-term conditions* ***to independently manage their conditions****. One such approach is social prescription.*(33) *– introduction*

*In contrast to the ‘light-touch’ approach (typically reported in UK studies), which could increase dependency on primary care for addressing social problems and welfare needs (Cawston, 2011), the ‘holistic’ model aims to improve a* ***patient’s self-efficacy and capacity to maintain or improve their health and well-being over the longer term****.* (34) *– introduction*

*It was assumed that SP would not only improve patients’ mental well-being and* ***‘activate’ them to better self-manage their health****, but would also lead to reduced demand on primary and acute health care and social care services.* (34) *– introduction*

*The primary hypothesis was that the intervention would improve health and social wellbeing, patient activation and frailty levels, and that this would lead to less use of primary, social and acute care services and reduced costs.* (34) *– introduction*

*The Patient* ***Empowerment*** *Project (PEP) was an intervention developed by NHS Leeds West Clinical Commissioning Group (CCG) to address unmet patient needs by increasing signposting to, and knowledge about, local services and voluntary groups that could provide appropriate support. PEP was also particularly focussed on the improvement of wellbeing* ***and self-management*** *for individuals with one (or more) of four long-term health conditions; depression, diabetes, chronic obstructive pulmonary disease (COPD) or cardiovascular disease (CVD).* (48) *– introduction*

*Developed with the aim of encouraging self-care and behaviour change, ‘social prescribing’ interventions (sometimes called ‘community referral interventions’) allow health-care practitioners to refer patients with LTCs to non-clinical services, primarily in the community and voluntary sectors. (18) – introduction*

*Social prescribing has been defined as: “harness[ing] assets within the voluntary and community sectors to improve and encourage self-care and facilitate health-creating communities.”*(49) *– background*

*Socially orientated approaches delivered through GPVCS collaborations may broaden community capacity (Friedli et al., 2012) and empower patients to better manage their own health and make more appropriate use of health services.*(40) *– introduction*

*SP Holistic projects are adopting a holistic and preventive approach and aim to work with beneficiaries with long-term conditions. They encourage beneficiaries to play a central role in* ***managing their own care****. (23) – introduction*

*Social prescribing has been defined as: ‘….options that make available new life opportunities that can add meaning, form new relationships, or give the* ***patient a chance to take responsibility*** *or be creative. Usually these services need to be available locally and often within the voluntary, community, and social enterprise sector (‘third sector’)’. (p. 454)* (36) *- Introduction*

*From dependence to independence: emerging lessons from the Rotherham Social Prescribing Pilot* (29)  *- title*

**Code 2.4. Intervention characteristics: coaching, motivational strategies, health education/lifestyle-related, time-bound, led by coaches trained in motivational techniques**

*Given that the WBCs focus on wellbeing represents an asset-based approach, they perceive a key benefit of supporting clients and carers to be facilitating a* ***change in their attitude and raising expectations about what they can do*** *to remain well.* (37) *– results*

*The service comprises: (A) individual assessment, motivational interviewing and action planning; (B) completion of an initial ‘Well-being Star’ assessment and subsequent Well-being Star assessments every 6 months thereafter for the duration of the patient’s involvement; (C) help to access community services (eg, welfare rights advice, walking groups, physical activity classes, arts groups, continuing education); (D) promotion of volunteering opportunities, and; (E) promotion of improved self-care and sustained behaviour change related to healthier lifestyle choices.*(32) *– introduction*

*home or at the clinic to be conducted as a face-to-face encounter held in private. On completing the assessment, achievable goals were agreed in terms of any actions or changes in lifestyle that the client would undertake, specific support that the WBC intended to provide and a realistic timeframe* (37) *– methods*

*• Individually-tailored lifestyle advice, enhanced through peer support, and evidence-informed behaviour change techniques • Specialist nutrition, healthy eating, and physical activity advice • Support for promoting wellbeing and managing stress, anxiety and depression(13) – methods*

*Features of the Connect service: An ‘Asset Mapping’ exercise was undertaken to identify available services across third, public and private sectors, self-help, self-management resources, educational, leisure and recreational facilities and ﬁtness-, health- and exercise-related*(50) *– methods*

*The “Ways to Wellbeing” social prescribing service provides opportunities for patients to learn life skills-based cognitive behavioural approaches, mindfulness, self-care strategies, sleep hygiene and relaxation techniques alongside hobby and interest groups such as singing, dancing and knitting clubs. […] All the social prescriptions have a core educational component focussing on problem solving skills, goals setting and developing an awareness of the Five Ways to Wellbeing (Aked et al., 2008). The courses are delivered at community venues across the borough as a means of reconnecting patients with community assets that can bolster a sense of wellbeing. All patients complete a “moving forward” plan towards the end of their intervention in which they map out assets of people and place that can help them to stay engaged and active in the community so they are able to maintain their progress (see Box 6).(2) – methods*

*Participants were ‘linked’ to a wide range of community groups and services, including gyms, walking groups and exercise classes; weight-loss and healthy eating groups; and LTC management groups such as breathing exercises for people living with respiratory conditions. (18) – results*

*All participants attended a weekly arts and crafts group (2–3 h for 10 weeks); groups were led by a practicing artist/instructor and co-facilitated by a mental health social worker who maintained communication with participants throughout the program, and provided additional supports and adjustments pre- and post-activities (10) – methods*

*Wellbeing co-ordinators dealt with a range of needs from straightforward sign-posting to, what was in essence, a more intensive coaching-style intervention. Some of the most positive outcomes reported by patients resulted from experiencing sessions which allowed them the time to explore their situation more fully and work collaboratively to set realistic goals for the future. (19) – results*

*Thus, social prescribing seems to work for all those patients who need support and* ***motivation*** *to act upon improving their own health and well-being, particularly if their needs are non-clinical or have a non-clinical component.* (30) *– discussion*

*It often involved patient referral from primary care services to a link worker or ‘navigator’ who* ***helps empower them to make changes*** *to their lives through accessing community support interventions* (51) *– results*

*The level of intervention offered by the SPC may vary considerably from straightforward signposting, requiring a detailed knowledge of local organisations available to the patient, to a more intensive coaching-style intervention for those patients needing to overcome barriers before moving on to the next step.* (30) *– results*

*The coordinator uses motivational interviewing and coaching-based skills to identify issues and challenges, manage anxiety and help patients identify possible steps forward. Patients are then referred into appropriate services that can support their health and wellbeing. Some, where relevant, are offered further face-to-face sessions, up to six (level 3) to provide further coaching-based support to help identify goals and develop personal action plans.* (31) *– methods*

*The Co-ordinator then works with the individual for up to 12 weeks to enable them to take action to achieve their goals. This includes resilience-focused coaching and practical support and advocacy to navigate and access local health, social and economic services. This study focuses just on those participants receiving the more intensive ‘holistic’ intervention.* (34) *– methods*

*To ensure clients can make the most of onward referrals and sign-posting Social Prescribers use a range of motivational interviewing, goal-setting and coaching skills in their discussions with clients. (5) – methods*

*The well-being coach then contacts the patient and schedules an appointment for a one-on-one intake session lasting 1 h. The intake session takes place either at the participant’s house or in the community well-being centre. During the intake session, a well-being coach uses a strengths-based approach to evaluate the participant’s life in a holistic manner. The patient’s sources of positive energy and strength are systematically identiﬁed. Additionally, possible barriers to thriving are also explored so that they can be addressed throughout the process. The well-being coach uses a step-by-step approach that focusses on what the participant enjoys doing. For example, the coach may ask ‘What were you good at previously?’ The coach aims to reinforce the patient’s self-efﬁcacy and self-reliance through social activation.*(42) *– introduction*

***The use of cognitive‐behavioural, motivational and ‘persuasive’ techniques*** *by activity leaders was linked to Adherence, and participants’ relationship with the leader also acted as a motivating factor.* (39) *– results*

*Following a referral, navigators then contacted referred patients to arrange an initial appointment held in surgeries. The role of navigators involved an individual assessment to identify the non-medical needs of service users, motivational interviewing, continuous personalised support and to link service users with non-medical sources of support, to help improve their health and well-being.(16) – introduction*

*All Co-ordinators are non-health care staff (although some previously worked in the health service) and all received training in goal setting, use of tools and outcome measures, and in how to engage with users in a strengths-based way, co-produce a plan and manage risk. Key aspects of the role included: listening skills, emotional support, advice and practical assistance and coaching.*(34) *– methods*

*Following extensive consultation with patients and healthcare professionals over an 8-year period, Ways to Wellness provides a ‘hub’ model of social prescribing in which a Link Worker trained in behaviour change methods offers a holistic and personalised service.*(32) *– introduction*

*In the WtW model, patients are referred by a primary‐care practitioner to a link worker trained in behaviour change methods.(17) – introduction*

*All of the Social Prescribers are required to undertake the following training as part of their contract: • Motivational Interviewing • Making Every Contact Count • Information Governance • Basic Life Support • Safeguarding (5) – methods*

*There was an appreciation of the (often very basic) level of training they had received such as safeguarding and Mental Health First Aid and for those who had experienced it, training in behaviour change techniques such as Motivational Interviewing was valued.*(35) *– results*

*We offered brief interventions training to health professionals to help them to respond more effectively to patients’ social needs. The training provided insights into the Five Ways to Wellbeing (Aked et al., 2008), motivational interviewing and the BATHE technique (Stuart and Lieberman, 2008). Feedback from staff about this training showed that they found it useful in their consultations with patients.(2) – results*

*Link workers are trained in behaviour change methods, such as motivational interviewing techniques, that help service users identify which areas of their lives they wish to change and how. These techniques emphasis service users' choice and control over their decisions and behaviours. (18) – introduction*

*Building self-confidence, self-reliance and independence was another facet of the Link Workers’ approach, managed through ongoing support and persistence in finding the right motivational tools for the individual, while conveying the need for personal responsibility and resilience. This enabled service users to make changes to their lives, engage with other organisations and manage their long-term conditions.* (32) *– results*

*In some SP pathways, health coaches (HCs) receive these referrals and provide patients with practical and emotional support, as well as opportunities to better manage their own health.*(38) *– introduction*

*Once recruited onto the pathway, patients have an initial meeting and needs assessment with an HC, who either prescribes self- care management or refers to an LW, who in turn connects the patient with relevant third-sector groups.*(38) *– introduction*

*The best example of this approach could be found outside of the CCG area. It was developed by senior partner who had been a GP in his town for 26 years. Like other SP initiatives their intervention included the employment of a Health Facilitator based in the practice. This role developed out of an exercise on prescription scheme developed by the local surgeries and the local Council ten years before. The health facilitator sees referred patients. Using Life Check and other tools the facilitator provided advice on exercise, nutrition, diet etc. They promote self-care using an on line Thought Field Therapy programme (rather like CBT) and also signpost to voluntary organisations or self-help groups for specific disease areas* (52) *– results*

*Features of the Connect service An ‘Asset Mapping’ exercise was undertaken to identify available services across third, public and private sectors, self-help, self-management resources, educational, leisure and recreational facilities and ﬁtness-, health- and exercise-related*(50) *– introduction*

*The “Ways to Wellbeing” social prescribing service provides opportunities for patients to learn life skills-based cognitive behavioural approaches, mindfulness, self-care strategies, sleep hygiene and relaxation techniques alongside hobby and interest groups such as singing, dancing and knitting clubs.(2) – design/methodology/approach*

*In both phases of the study, link workers reflected that the intense support required by some clients meant that it was vital to set boundaries around expectations of the nature of support on offer. Perhaps the trickiest and most sensitive aspect of boundary‐setting was managing clients’ expectations around relationships. A strong, supportive link worker/client relationship is vital for successful social prescribing (Moffatt, Steer, Penn, & Lawson, 2017). Nevertheless, relationship boundaries were not always easy to set and required careful management, with link workers describing “a bit of a balancing act” between being a “friend but not a friend” (P13, Interview, Phase 1). A useful strategy for managing client dependency involved referring clients to specialist services and utilising the multi‐agency approach suggested in the link worker training. By follow‐up, link workers had established relationships with some clients over a period of months. Dependency continued to be identified as an issue, with link workers expressing concerns both over the risk of client dependency and of themselves becoming “too emotionally involved” with clients who “are not seeing you as their professional worker but as their friend” (P2, FG3, Phase 2). Additional strategies for maintaining appropriate boundaries had been developed over time, including regularly reminding clients of the limits of the link worker role, creating distance by doubling‐up, swapping link workers or running group activities and reasserting the importance of empowerment rather than dependency. (17) – results*

*To ensure* ***clients*** *can make the most of onward referrals and sign-posting Social Prescribers use a range of motivational interviewing, goal-setting and coaching skills in their discussions with clients. (5) – results*

*The client is then contacted within seven days to arrange an appointment. Although people are encouraged to come to their local general practice surgery, it is possible for home visits to be arranged. Up to three appointments of approximately up to 40 min each are held with clients to discuss their needs and to then identify an appropriate source of local support. The CHAT worker facilitates access to local organisations, predominately from the voluntary and community sector (1) – introduction*

*After assessment, service users can potentially access a range of community and voluntary sector support. To avoid dependency on the social prescribing service, individuals are encouraged to ‘exit’ the service or are referred to other health and social care providers after 6 sessions.*(49) *– background*

*The service* ***extended choice*** *for a wide range of patients, represented a viable alternative to CBT and medication and represented a suitable option for those experiencing isolation and frequent attenders.*(50) *– introduction*

**Code 2.5. Characterisation of SP users: at risk of being/becoming dependent, lacking control, lacking motivation, frequent attenders**

*Thus, social prescribing seems to work for all those patients who need support and* ***motivation*** *to act upon improving their own health and well-being, particularly if their needs are non-clinical or have a non-clinical component.* (30) *– discussion*

*Prior to the project participants expressed their inability to engage with people or have thoughts about the future, suggesting a lack of a* ***sense of coherence****. On completion of the project, the feelings that participants expressed about themselves and their surroundings put forward positive changes for themselves and a step towards creating meaning in their lives and connecting with other people.(22) – results*

*There is a danger of patients becoming dependent on a link worker as the source of support; this should be tempered if individuals create new and meaningful connections within the community, which may include reconnecting with friends and family because of a more positive outlook on life. Such an improved outlook may encourage those with existing health conditions to actively* ***engage in self-care.*** *(25) – results*

*In contrast to the ‘light-touch’ approach (typically reported in UK studies), which could increase dependency on primary care for addressing social problems and welfare needs (Cawston, 2011), the ‘holistic’ model aims to improve a* ***patient’s self-efficacy and capacity to maintain or improve their health and well-being over the longer term****.* (34) *– introduction*

*The CW service was available to anyone aged over 75 years and frequent attenders at the practice*(37) *– methods*

*The target group for Welzijn op Recept comprises patients who frequently visit their GP or other primary care provider about psychosocial problems for which no medical cause can be found (trouble sleeping, worrying a lot, feeling depressed, etc.).*(42) *– introduction*

*Several studies reported that social isolation and low mood due to life circumstances such as unemployment were the key reasons for referral to social prescribing (The Care Forum, 2012). Social prescribing was considered suitable for frequent attendees to health services or those with inexplicable symptoms (NESTA, 2013).*(53) *– results*

*As individuals feel more socially included and self-confident through joining groups and receiving helpful outside advice,* ***they become less reliant on their GP****. (25) C2.5. – results*

*While not all participants were asked about this issue, as it only arose during one discussion group, those who were asked felt very strongly that the service and support should be ongoing. This issue should be explored and the implications of offering the service on a continuing basis should be identified. While on the one hand the participants clearly felt that this was important, and that without it they might fall back into old habits, there is also a need to encourage people to take responsibility for themselves and their health, and not become too dependent on health staff or services. This issue has been previously raised by Hunt and Hillsdon [18]. Furthermore, if participants were able to continue seeing the AL advisor it would increase demand and time pressure on an already stretched service.* (54) *– discussion*

*The dependency that a lot of participants clearly have for health services and staff was apparent during discussions regarding other aspects of the service. Some participants suggested that having their measurements taken by the AL advisor at consultation, such as blood pressure and body weight, helped them to stay motivated, and that without this they would not have stuck to their goals* (54) *– results*

*There is a danger of patients becoming dependent on a link worker as the source of support; this should be tempered if individuals create new and meaningful connections within the community, which may include reconnecting with friends and family because of a more positive outlook on life. (25) – results*

*Reﬂecting on the CHAT experience, for some the initial appointment is all that is needed, as this can give the necessary space for reﬂection as individuals beneﬁt from the process of being listened to and starting to voice their aspirations. It is not suitable for those who require an intensive package of support* ***and the limit on number of appointments prevents dependency****. The evaluation found that the longer appointment time in comparison to clinical consultations was valued, as was the offer to accompany the client to a new group.(1) – results*

*In both phases of the study, link workers reflected that the intense support required by some clients meant that it was vital to set boundaries around expectations of the nature of support on offer. Perhaps the trickiest and most sensitive aspect of boundary‐setting was managing clients’ expectations around relationships. A strong, supportive link worker/client relationship is vital for successful social prescribing (Moffatt, Steer, Penn, & Lawson, 2017). Nevertheless, relationship boundaries were not always easy to set and required careful management, with link workers describing “a bit of a balancing act” between being a “friend but not a friend” (P13, Interview, Phase 1). A useful strategy for managing client dependency involved referring clients to specialist services and utilising the multi‐agency approach suggested in the link worker training. By follow‐up, link workers had established relationships with some clients over a period of months. Dependency continued to be identified as an issue, with link workers expressing concerns both over the risk of client dependency and of themselves becoming “too emotionally involved” with clients who “are not seeing you as their professional worker but as their friend” (P2, FG3, Phase 2). Additional strategies for maintaining appropriate boundaries had been developed over time, including regularly reminding clients of the limits of the link worker role, creating distance by doubling‐up, swapping link workers or running group activities and reasserting the importance of empowerment rather than dependency. (17) – results*

*After assessment, service users can potentially access a range of community and voluntary sector support. To avoid dependency on the social prescribing service, individuals are encouraged to ‘exit’ the service or are referred to other health and social care providers after 6 sessions.*(49) *– background*

*Specifically,* ***clients*** *identified a close client/link worker relationship and link worker continuity as important factors in service engagement and in making and maintaining lifestyle changes (Moffatt et al., 2017; Wildman et al., in press). In this study, we identify a risk of dependency arising from this close relationship, with link workers sharing a range of strategies developed over time to mitigate this risk. (17) – discussion*

**Code 2.6. Evaluation approaches in line with Discourse 2:**

- **Code 2.6.1. Theoretical frameworks**
- Salutogenesis/asset-based model. Used by (37), (22), (2), (55)
- Learned mastery theory. Used by (21)
- Social cognitive theory. Used by (30), (13)
- Self-determination theory. Used by (56), (57), (58), (59), (60), (13)
- The social cure theory. Used by (38)
- Patient Activation Theory. Used by (25)
- Control Theory. Used by (13)
- Theory of Planned Behaviour. Used by (13)

*The programme targets factors identified by various social cognitive models, for instance the* ***Theory of Planned Behaviour****, as being important in motivating people to change their behaviour (e.g., risk perceptions, pros and cons of behaviour change, self-efficacy). The programme also adopts motivational approaches from* ***Self-determination Theory*** *(Deci & Ryan, 2000), such as supporting intrinsic motivation, engaging social support/encouraging connectedness and building competence. The core intervention is based around use of behaviour change processes and techniques that derive from self-regulation theories, such as* ***the Social Cognitive Theory*** *(Bandura, 1985)* ***and Control Theory*** *(Carver et al., 2000). These include goal setting, action planning, self-monitoring, feedback on progress, problem solving, and reviewing goals, which are suggested to be important in translating motivations into action, and supporting longer-term maintenance of behaviour change (e.g. Michie & Johnson, 2012). The intervention providers have been trained to use patient-centred counselling approaches (e.g., motivational interviewing) in the delivery of these techniques, which appear to enhance weight loss in overweight and obese participants (Armstrong et al., 2011).(13) – methods*

*The Salutogenesis model represents a humanistic philosophy with a person- centred approach. Antonovsky’s focus is on resources, competences and promoting of the ‘healthy’ (Salutogenesis) contrary to an emphasis on diseases, malfunctions and risk factors (pathogenesis). In this way, Salutogenesis is a health practice that is oriented towards what already exists and what makes someone healthier and works from the perspective that people are always somewhere on a continuum between sick and healthy, and while, for example, at the more ‘sick’ end of the continuum, there will also be some ‘healthy’ dimensions in life.17 […] Meaningfulness.* ***Meaningfulness, which Antonovsky sees as the single most important factor to survive, adopt and overcome difficulties, is linked to how much an individual feels that life makes sense****, and if challenges are worthy of commitment.17 Some of the participants commented that prior to the project they had lacked a sense of meaning and purpose for getting up in the morning and engaging in the wider world.(22) – methods*

*We invested a considerable amount of time in the early stages developing and refining a theory of change that would describe the way that the CWP model would bring about improvements in health. Key concepts that informed our theory were: salutogenesis – developed by Aaron Antonovsky (1979), which focusses on the determinants of good health with a particular focus on people’s resources, capabilities and the mechanisms that create and sustain health. We also incorporated asset-based approaches to health (Foot and Hopkins, 2010; Rippon and Hopkins, 2015) and insights into how people are able to build a sense of control and autonomy in their lives (Sen, 1999; Whitehead et al., 2016).(2) – methods*

*The PSS may also be seen as empowering patients to* ***take control*** *over their lives. For example, a recent factor analysis of empowerment in healthcare outlines components of care that are similar to those provided by the PSS [14], such as providing patients with information and* ***choices*** *concerning their future care. The concept of Empowerment has been empirically linked to* ***learned mastery*** *theory [15] whereby patients who are given control over their lives become increasingly* ***motivated****. (21) – discussion*

*Social cognitive theory is one of the chosen conceptual models that underpins the process of behavioural change (Bandura, 1986). In social prescribing, behavioural change leads to improved mental and physical well-being in three key ways: ﬁrst, the combined effect of one-to-one interaction between the patient and SPC in the form of coaching, motivation and listening (Prochaska and Norcross, 2009); second, the social interaction between the patient and the group of people involved in running community activities; and third, the social interaction within other community activities. Through the support received from SPCs and social interaction in the community, patients move through different stages, ultimately ﬁnding themselves empowered to change their own circumstances (Hibbard and Gilburt, 2014) – methods*

*We have used* ***NPT*** *to guide the development of PLANS in a way in which incremental changes could be made on the bases of feedback at diﬀerent stages from patients, and with reference to the technological, primary care and community settings, the tool was orientated to operate within. Of particular, salience is patient normalization. That is, to be an optimal candidate for normalization, a new tool (such as the one proposed here) should seek a ‘ﬁt’ with the actual or realizable set of roles within patients’ division of labour and be capable of integration within existing or realizable patterns of self-management and service contact with professionals. It follows from this that the advantage to patients must be tangible and evident to their everyday illness work and contact with services is crucial to the evaluation of new interventions and practices.(20) – methods*

*Self-Determination Theory (SDT; Deci & Ryan, 1985, 2000) appears to hold considerable promise for elucidating the social psychological processes inﬂuencing exercise participation. SDT postulates that an autonomy supportive context will foster the satisfaction of three basic psychological needs (i.e., the needs for autonomy, relatedness and competence). When these needs are satisﬁed, it is assumed that self-determined forms of motivational regulation guide behaviour (i.e., intrinsic motivation and integrated and identiﬁed regulation) and adaptive behavioural (e.g., exercise engagement), cognitive (e.g., commitment) and well-being (e.g., vitality) outcomes are postulated to ensue. In contrast, diminished need satisfaction elicits less or non self-determined motivation (i.e., introjected and external regulation and amotivation), which in turn, results in maladaptive outcomes. […] The main objective of the current study was to examine whether overweight/obese individuals who adhered more to their exercise prescriptions reported greater levels of autonomy support, psychological need satisfaction and self-determined motivational regulations, versus those who adhered less. Moreover, given that motivational processes are expected to impact a multitude of cognitive, affective and behavioural outcomes (Vallerand, 1997, 2001), this study also examined whether those individuals that adhered more reported greater levels of exercise behaviour, exercise-related cognitions (i.e., self-efﬁcacy, commitment and behavioural intention) and general well-being (i.e., positive and negative affect, subjective vitality and satisfaction with life). We hypothesized that those individuals who adhered more would report higher levels of autonomy support, psychological need satisfaction, self-determined motivation and positive behavioural, cognitive and affective outcomes at 3-months, as well as a greater increase in these constructs over time, compared to those who adhered less. Secondly, we explored the motivational sequence embedded in SDT by testing autonomy support as a predictor of need satisfaction, autonomy support and need satisfaction as predictors of the motivational regulations, and autonomy support, need satisfaction and the motivational regulations as predictors of behavioural, cognitive and well-being outcomes. We hypothesized that, over time, perceived autonomy support would emerge as a positive predictor of psychological need satisfaction. Autonomy support and psychological need satisfaction were hypothesized to predict self-determined motivation. […] Further, autonomy support, need satisfaction and self-determined regulation were hypothesized to predict adaptive behavioural and cognitive exercise-related outcomes, as well as well-being, over the course of the 3-month exercise prescription. In this way, we aimed to garner a better understanding of how different facets of SDT impact upon different components of the exercise experience. Such information can be used by practitioners to more effectively facilitate each of these consequences in exercise settings.*(56) *– Introduction*

*Self-Determination Theory (SDT) is a psychological theory concerning human wellbeing, motivation and behaviour change,9,10 which has been applied to understanding how new behaviours can be achieved and sustained in people’s lives.11 SDT argues that this requires the satisfaction of three innate needs: autonomy (a sense of control over one’s own activities and behaviours), competence (a sense of ability to inﬂuence outcomes) and relatedness (a sense of connection to and interaction with others) (Figure 1). Proponents of SDT argue that satisfaction of all three needs is necessary for an individual’s actions to be ‘self-determined’, and that actions which are more self-determined are likely to be maintained and to lead to greater wellbeing.[…] We use SDT to explore participants’ experience of the Links Worker Programme. This study aims to investigate if SDT can be used to understand the change, or lack of change, resulting from patients’ involvement in the Links Worker Programme[…]* (57) *– introduction*

*Participants accounts covered a range of categories of regulation described within the SDT framework. Motivation relating to different aspects of life, and at different time points, was expressed in different terms. As such, regulation was not a ﬁxed state. There was variation within and between participants, regardless of the assessed impact of the intervention (Figure 2). However, in patients who described signiﬁcant change resulting from the Links Worker Programme, there was a contrast between their descriptions of regulation of behaviours before and after seeing the CLP. […] Circumstances before referral were often described in terms of amotivation or external regulation, whereas circumstances following the intervention often described identiﬁed or integrated regulation (Figure 3).* (57) *– results*

*Research applying SDT to health behaviours has shown that satisfaction of the psychological needs for relatedness, competence and autonomy is associated with more purposeful, consistent and sustained behaviour change.* (57) *– discussion*

*According to self-determination theory (SDT),4 intrinsic motivation represents the most self-determined form of motivation and, in addition to predicting exercise adherence, has been found to be related to positive self-evaluation.5 When people are motivated for extrinsic reasons (e.g. guilt, rewards), they are said to be less self-determined and are described as being extrinsically motivated.4 Finally, when people can no longer identify any reason for engaging in a given behaviour, they are said to be amotivated.4 Intrinsic motivation is unlikely to be prevalent at the beginning of an exercise programme6 and typically has to be fostered, as the enjoyment and stimulation from the activity itself may not be sufﬁcient to encourage adherence.7 Patients are often inactive when they are referred to these programmes, and so in order for them to accrue the necessary physical and mental health beneﬁts that may stem from their sustained involvement in exercise, it is ﬁrst important to understand the key mechanisms that may support their early involvement in such schemes. This requires an understanding of whether the ERS actually fosters self-determined motivation, and subsequent exercise behaviour*(58) *– introduction*

*Boosting autonomy, relatedness and competence are key components for increasing self-determined motivation.4 Specifically, by observing the (competent) exercise behaviours of an exercise partner (with a similar history of ill health), it was thought that patients’ beliefs that they too could succeed in the programme could be fostered, thus enhancing self-efﬁcacy.* (58) *– results*

*One such framework is Self-Determination Theory (SDT; Deci & Ryan, 1985) which considers behavioural regulation and factors affecting this. According to SDT, behavioural regulation can be amotivated, extrinsically or intrinsically motivated, differing in their degree of self-determination. Amotivation is the relative absence of motivation (Deci & Ryan, 2000). Extrinsic motivation refers to the motivation to engage in a behaviour because it leads to a separate consequence, such as a reward (Deci & Ryan, 2008), and is comprised of four increasingly self-determined regulations (Ryan & Deci, 2000). External regulation is when an individual’s behaviour is controlled by external rewards or the threat of external punishments which undermine self-determined motivation (Deci, Koestner, & Ryan, 1999). Introjected regulation occurs when an individual acts out of internal pressure or guilt or to satisfy self-worth. Identified regulation reflects the individual’s acceptance of the value of a behaviour, even if it is not enjoyable. Integrated regulation reflects a valued behaviour that is integrated into other life roles (Deci & Ryan, 2000). Intrinsic motivation is fully self-determined and represents the motivation to participate in inherently enjoyable behaviours without external rewards (Deci & Ryan, 2002. […] Cognitive Evaluation Theory (CET; Deci & Ryan, 1985, 2002), a sub-theory of SDT, deals with the factors that undermine or facilitate intrinsically motivated behaviour. It results from satisfying three innate psychological needs: the need for autonomy (feeling self-determined in one’s actions and not controlled by external factors; deCharms, 1968), competence (perceiving one has the ability to deal effectively with the environment and to effect outcomes) and relatedness (feeling connected to others and having supportive relationships; Reis, Sheldon, Gable, Roscoe, & Ryan, 2000). If social and/or environmental factors fail to satisfy any of these needs, diminished motivation and well-being are likely (Wilson, Rodgers, Blanchard, & Gessell, 2003).*(59) *– introduction*

*SDT (Deci & Ryan, 2000) is concerned with why we engage in specific behaviours and focuses on the degree to which people’s motivation towards engagement in activities, such as PA, are more or less self-determined or controlled by external or internal pressures. SDT proposes that when an activity is not intrinsically motivating, behaviour is guided by a variety of extrinsic regulations which are assumed to lie on a self-determination continuum (Ryan & Deci, 2002). External regulation is the least autonomous and indicates a behaviour that is conducted for tangible and intangible rewards, externally referenced reinforcement or as an outcome of pressure from external sources. As we progress along the continuum, introjected regulation represents the motive to perform a behaviour to avoid guilt and shame or attain feelings of contingent self-worth and social approval. Identified regulation reflects engagement due to an understanding, acceptance and valuing of the benefits associated with participating in the behaviour. The most self-determined regulation, intrinsic motivation, reflects an inherent interest in the activity in hand and does not necessitate any operationally separable consequences (Deci & Ryan, 2000). Deci and Ryan (2000) indicate that these regulations cluster to form autonomous (intrinsic and identified) versus controlled (introjected and external) regulations. SDT further proposes an amotivated state in which an individual lacks any intention or desire to conduct the behaviour.*(60) *– introduction*

*The pathway evaluation reported here is underpinned by an appropriate psychosocial framework: the social identity approach to health and well- being, aptly named ‘The Social Cure’ (SC44 45). This approach posits that our social group memberships (eg, family, community, volunteering group) are consequential for our social life, health and well- being, but only if we identify with them (ie, feel a subjective sense of group belonging46). Group identification is believed to enhance social life and well- being through numerous benefits, such as reduced loneliness, enhanced self- esteem and the belief that social support will be available during crisis.* (38) *– introduction*

*The term corrective emotional experience (Alexander & French, 1946) is introduced to designate learning or more precisely behavioral change during an intervention. Originally it describes the crucial process that takes place during psychotherapy, whereby the therapeutic setting is seen as a context in which patients are re-exposed in a secure environment – made up, for example, by the empathy and awareness of the therapist (Streeck, 2007; Streeck & Leichsenring, 2009) – to emotional situations that they could not handle in the past. In order to be helped, the patient must undergo a corrective emotional experience that can repair the traumatic inﬂuence of previous experiences. In this the therapist adopts a holding attitude (Winnicott, 1964), which makes it possible for the patient ﬁrst to undergo a therapeutic regression, then to have a corrective emotional experience, and afterwards to try out new behavior, initially in the secure environment and later in daily life. The idea of a possible correction of emotions by using certain experiences has been adapted to health science, for example to affect a moderation of dental anxiety among adults (Eli, Uziel, Blumensohn & Baht, 2004) or to conceptualize responsiveness of the nursing staff in health services (Rafferty, 2000). The corrective emotional experience in the context of an exercise program is understood as a re-education of emotions that is linked to physical activity. […] ﻿The first research aim question the role of existential aspects such as suffering and fear of death in order to understand personal motives for adopting an active and healthy lifestyle. The second research aim questions the role of a corrective emotional experience for the change of exercise behavior. The third research aim, questioning whether the experience of other relevant individuals, such as the group or the physiotherapist in an Exercise on prescription program, will support behavioral change, receives particular attention* (61) *– introduction*

*Patient activation is defined as people’s confidence, motivation and ability (skills/knowledge) to manage their health [27]. Patient activation brings into focus attitudes and beliefs as well as behaviours and knowledge [28]. The problem, according to Hibbard [29], is many providers just give patients information without understanding where they are in terms of believing they can control their health situation. This may be unsuccessful in assisting individuals with low activation levels, as they can feel overwhelmed by and have limited confidence in managing their health [27]. It is argued that by tailoring an intervention to someone’s activation levels, they are more likely to encounter small successes, which propels them forward rather than leaving them deterred due to a lack of achievement [27]. A patient activation measure (PAM) has been developed to gauge how motivated and able someone is to manage their health [30, 31]. People identified as activated on this measure appear more likely to adopt healthy behaviours (e.g. diet and exercise) and to have less hospital use [27]. Intervention components linked to increasing patient activation scores include those that help with skills development, problem-solving, peer support or engender change in beliefs and social norms [32]. Link workers can cover these components (e.g. encouraging patients to think of assets and solutions to their problems when co-producing an action plan, linking them to networks that can foster connections). Through feeling more activated, a patient may be motivated to invest in self-care, prompting them to visit a GP for advice.(25) – results*

*Williams et al. (2006b) conceptualised autonomy support as features of the social environment created by significant other(s), such as eliciting and acknowledging perspectives, supporting self-initiative, offering choice, providing relevant information and minimising pressure and control. For example, a health and fitness advisor who creates an autonomy supportive environment offers his/her client the opportunity to choose the activity that he/she will engage in (Pelletier et al., 2001b; Williams, Cox, Kouides, & Deci, 1999), acknowledges positive and negative feelings towards becoming physically active in an empathetic manner (Edmunds et al., 2007), understands the client’s perspective (Pelletier et al., 2001b; Reeve & Jang, 2006) and encourages ownership and self-initiative towards becoming physically active (Deci & Ryan, 2000). SDT proposes that when an autonomy supportive environment is created, the reasons for conducting a behaviour become more self-determined or autonomous over time. Previous study in the physical domain has provided evidence for this assumption (Edmunds et al., 2008; Hagger et al., 2009; Tessier et al., 2008).*(60) *– introduction*

*In particular, the relationship between patient and SPC deserves further attention as it appears to translate into practice some of the theories underpinning social cognitive theory, self-efﬁcacy (Coulter and Ellins, 2006), motivation (Hibbard and Gilburt, 2014) which have been discussed in the previous section.* (30) *– discussion*

*Future research in this area should concentrate on testing these theories by measuring quantitatively the impact of the SPC on patients, in addition to measuring changes in patients’ health outcomes across the pathway as a whole. A suitable comparison group with no access to SPC should be identiﬁed, possibly through randomised selection. This exercise would ﬁnally provide us with greater understanding of the role of SPCs in patients’ health and well-being and enable us to test social cognitive theory and develop a more precise framework for patient activation (Hibbard and Gilburt, 2014).* (30) *– discussion*

***﻿****The assets-based model brings together the three main strands of the South West Well-being programme. Personal well-being assets underpin these strands and emphasise the central role of psychological resources such as confidence, self esteem and autonomy. Similarly in this model, social well-being assets - such as community engagement, belonging, trust and social support – act as mediators for the promotion of physical activity, healthy eating and mental health.* (55)

- **Code 2.6.2. Outcomes related to self-concept, self-management, behavioural change, independence, activation**

Studies with outcome variables in line with code 2.6.2, such as:

- WEMWBS (methods/results): (7), (49), (62), (53), (4), (34), (63), (14), (64), (2), (37)**,** (31), (65), (3), (66), (67), (68), (44), (69), (48), (70)
- Other questionnaires/outcomes about self-confidence, self-efficacy, activation, motivation, independence (methods/results): (71), (34), (14), (72), (64), (73), (74)**,** (75), (76), (9), (56), (77), (59), (58), (78), (13), (60), (48), (10)

*With the support of new social networks, improvements in self-perception enabled clients to* ***become more confident, independent, and resilient despite*** *the challenges they continued to face in everyday life. Improvements in communication and social skills allowed clients to be* ***confident and assertive****, and practical, life-based skills contributed to increased resilience and Independence. […]* ***Improvements in confidence, self- esteem, independence, and motivation*** *enabled clients not only to set new goals, but also to actively pursue them. This was also facilitated by hope and optimism for the future, with regard to a client’s potential to succeed: ‘… since I’ve lost weight I feel a lot better in my health so I’ve got to carry on … I don’t want to be in that predicament again … I’ve got a lot of work to do, I’ve got to lose some weight and get healthier, and I’m hoping when we get swimming and things it’ll give me motivation.’ (Pamela)(79) - results*

*The overall hypothesis underpinning the realist evaluation of social prescribing in City and Hackney is that social prescribing improves well-being outcomes for patients suffering from isolation, and mild mental health problems. It provides a mechanism of support that enables each individual participant to consider a set of actions they may be willing to take, and thus embark on the journey to socially* ***re-activate themselves****, change their behaviour and, ultimately, their health. […] As a result of one or more consultations, the SPC helps the patient in accessing relevant activities available from statutory and community sectors which could contribute to the ‘activation’ of the patient. The mechanism underpinning this stage of the intervention is the interaction between SPC and patient.* (30) *– results*

*Thus, when patients are given agency and control over their time with non-imposing support from qualiﬁed SPCs who are empathetic and have a good knowledge of the social support infrastructure available locally, social prescribing is likely to have a beneﬁcial impact on service users, […] From a more conceptual point of view, when the relationship between SPC and patient develops successfully, the patient develops a strong sense of* ***self-efﬁcacy****,* ***feeling of control*** *and a willingness to take on and persist with new and difﬁcult tasks (Coulter and Ellins, 2006).* (30) *– results*

*As one interviewee put it, ‘it’s all about focusing on what you can still do, not what you can’t’. The obstacles people talked about and how they overcame them were less to do with money, or transport, or pills, much more to do with building the confidence* (33) *– results*

*Through these techniques, volunteers were able to generate a detailed picture of a patient’s circumstances and gauge the root cause(s) of their problems. This was vital in assessing the patient’s needs and in deciding upon the most appropriate community services to refer them to. Volunteers also felt that this increased patient self-awareness and* ***ownership of their problems****, an important step towards them ﬁnding a solution. […] The PSS may also be seen as empowering patients to* ***take control*** *over their lives. For example, a recent factor analysis of empowerment in healthcare outlines components of care that are similar to those provided by the PSS [14], such as providing patients with information and* ***choices*** *concerning their future care. The concept of Empowerment has been empirically linked to learned mastery theory [15] whereby patients who are given control over their lives become increasingly motivated [16]. (21) – results*

*The term self-concept refers to the mental representations that individuals use to reason about themselves. Self-concepts include self-beliefs, self-appraisal of abilities, behaviours and characteristics. In contrast to health, wellbeing and health-related behaviours, changes in perceived self-concepts and feelings were explored in qualitative and descriptive research exclusively. Multiple qualitative studies found that participating in SP improved the self-esteem, self-value, and hope of service users and made them feel useful and worthwhile.16,27,29–31,37 Another commonly reported outcome for service users is an improvement in self-confidence. […] Qualitative findings show that SP has the potential to develop and enhance self-confidence and independence of service users, so that they feel able to travel with public transport, leave their house, get involved in new activities in their community, take control over their health and to manage their conditions.(14) – results*

*Many participants felt confident they could continue with the* ***coping strategies and changes they had*** *made earlier in their engagement with the intervention, or at least were growing in confidence now that they were “better at putting myself right” (P18, female, age 65–69) (18) – results*

*‘‘Empowerment’’ integrates concepts relating to taking part; not only referring to attending the scheme but to the* ***patients’ engagement with*** *what the scheme has to offer. Empowerment involves connecting with both the scheme protocols and the people. This core category refers to both the desire and the ability to take part; ‘‘Kate ... I feel proud of myself and that to me is my success’’ (FG2, 94).* (46) *– results*

*Through these techniques, volunteers were able to generate a detailed picture of a patient’s circumstances and gauge the root cause(s) of their problems. This was vital in assessing the patient’s needs and in deciding upon the most appropriate community services to refer them to. Volunteers also felt that this increased patient self-awareness and ownership of their problems, an important step towards them ﬁnding a solution.(21) – results*

*From a more conceptual point of view, when the relationship between SPC and patient develops successfully, the patient develops a strong sense of self-efﬁcacy, feeling of control and a willingness to take on and persist with new and difﬁcult tasks (Coulter and Ellins, 2006). The motivation and support offered by SPCs creates the basis for behaviour change such as greater willingness to participate in chosen community activities.* (30) *– discussion*

*Prior to the project participants expressed their inability to engage with people or have thoughts about the future, suggesting a lack of a* ***sense of coherence****. On completion of the project, the feelings that participants expressed about themselves and their surroundings put forward positive changes for themselves and a step towards creating meaning in their lives and connecting with other people.(22) – results*

*The range of self-reported behaviour changes reported in the earlier study [20] were also reported at follow-up and comprised achieving and maintaining positive changes in diet, physical activity and smoking cessation; improvements in mental health and self-confidence; decreased social isolation; and increased engagement in community activities. (18) – results*

*Short to medium‐term outcomes included were improvements in social isolation,* ***resilience*** *and* ***self‐care*** *and reductions in use of services.*(43) *– results*

*Building self-confidence, self-reliance and independence was another facet of the Link Workers’ approach, managed through ongoing support and persistence in finding the right motivational tools for the individual, while conveying the need for personal responsibility and resilience. This enabled service users to make changes to their lives, engage with other organisations and manage their long-term conditions.* (32) *– results*

*The principle behind social prescribing is that as service users become ‘linked’ back into their communities, the intervention can be withdrawn. (18) – results*

*The sample group comprised 20 Pacific women aged 40 years old and over who had been members of a GRx programme and had been discharged as independently active. The length of time from being discharged from the programme to interviews ranged from three weeks to two years. Being discharged as ‘****independently active’*** *means that these women are also ‘graduates’ of the programme. This signifies that they have achieved, or are on their way to achieving, the goals that they had set when they started the GRx programme.(80) – methods*

*The promotion of* ***patient self-management and resilience*** *is crucial to SP. It endeavours to ensure that they have skills to look after themselves. (23) – results*

*One item assessed health self-efficacy: “I am confident in my ability to take action when my health status changes”, rated 1 (strongly disagree) to 5 (strongly agree) (Herdman et al. 2011). Hospital Admissions One item measured number of hospital admissions (for any reason) in the previous 6 months. Note that due to small sample sizes and low incidence, this measure is not reported in the finding(10) – methods*

*The best example of this approach could be found outside of the CCG area. It was developed by senior partner who had been a GP in his town for 26 years. Like other SP initiatives their intervention included the employment of a Health Facilitator based in the practice. This role developed out of an exercise on prescription scheme developed by the local surgeries and the local Council ten years before. The health facilitator sees referred patients. Using Life Check and other tools the facilitator provided advice on exercise, nutrition, diet etc. They promote self-care using an on line Thought Field Therapy programme (rather like CBT) and also signpost to voluntary organisations or self-help groups for specific disease areas* (52) *– results*

*There was an almost universal belief among participants that willpower was vital in maintaining changes over the long term. While a link worker could “encourage and support” (P5, female, age 65–69), long-term change was about “taking responsibility for yourself…nobody else is going to do it” (P18, female, age 65–69) (18) – results*

*﻿For each of the 10 participants, a lengthy attendance of Arts on Prescription had acted as a catalyst for positive change. Participants reported increased self-confidence, improved social and communication skills, and increased motivation and aspiration.(27) – abstract/results*

*Attendance at Arts on Prescription gives people the opportunity for engagement with meaningful activities. People may develop skills and improve their confidence* (36) *– results*

*People had the opportunity to re-build and re-establish themselves, and to re-evaluate past negative experiences. New skills were developed and these skills created confidence (81) – results*

*Participants described their sense of confidence as having been brought out through being part of a group, feeling a sense of belonging and acceptance as well as learning skills that are transferable to other areas of their lives. Such skills may be described as being a combination of personal, social and vocational, not only recognized by themselves but also by others. (81) - discussion*

- **Code 2.6.3. Outcomes related to social and healthcare service utilisation**

Studies with outcome variables in line with code 2.6.3, such as:

- Cost savings, cost-effectiveness (methods/results): (7), (62), (53), (4), (5), (34), (82), (74)**,** (65), (75), (68), (83), (23), (84)
- Health service utilisation(methods/results): (62), (24), (53), (4), (41), (5), (34), (82), (63), (19), (72), (74)**,** (11), (50), (44), (45), (84), (69), (85), (48), (86), (87), (10), (38)

*Participants demonstrated improved perceived physical health, QoL, health self-efficacy, and reduced healthcare utilisation and costs (Rogers et al. 2008). (10) – introduction*

*In terms of burden on the health system, social prescribing can decrease the number of hospital admissions, outpatient visits, mean length of hospital stay, number of GP visits, allied health appointments and prescription medication usage (Kimberlee et al. 2014; Loughren et al. 2014; Rogers et al. 2008; Windle et al. 2009). The financial impact of these savings is also demonstrated (Kimberlee et al. 2014; Windle et al. 2009). A meta-analysis of social prescribing studies by Knapp et al. (2012) concluded that relatively low cost investments in community capital-building initiatives can result in sizeable public savings. (10) – introduction*

*There is scope for providing new and innovative interventions to* ***promote the self-management of chronic conditions potentially reducing the need for physician led care****. The aim of the social prescribing service was to improve patient well-being* ***and increase personal self-efficacy shown by a reduction in primary health care resource use.***  *(19) – introduction*

*Short to medium‐term outcomes included were improvements in social isolation,* ***resilience*** *and* ***self‐care*** *and reductions in use of services.*(43) *– results*

*[…] we hypothesise that possessing group memberships will positively predict a psychological sense of community belonging, which in turn will be associated with lower levels of loneliness. In turn, we propose that this serial mediation pathway will then predict service usage which, if supported, would constitute a particularly strict test of our SC model. Based on previous SP literature and the social identity approach, the two variables we expected to change during the pathway were patients’ service use (decrease) and participants’ number of group memberships (increase).* (38) *– methods*

*one-to-one signposting and individual support were expected to result in patients being more able to* ***acquire and use available skills, information and support which was expected to lead, in turn, to patients better self-management of health conditions****, better ability to navigate systems* (45) *– methods*

*One item assessed health self-efficacy: “I am confident in my ability to take action when my health status changes”, rated 1 (strongly disagree) to 5 (strongly agree) (Herdman et al. 2011). Hospital Admissions One item measured number of hospital admissions (for any reason) in the previous 6 months. Note that due to small sample sizes and low incidence, this measure is not reported in the finding(10) – methods*

*The economic evaluation assessed costs and potential savings due to SP.(74) – results*

*Primary and secondary outcome measures: Primary outcomes of interest were any measures of health and well-being and/or usage of health services. […] The primary outcomes of interest were any measures of health and well-being, including self-reported measures (eg, levels of physical activity or depression scores) and/or measure of usage of health services. We also considered any other outcomes (eg, health service usage) reported in the included evaluations.* (62) *– abstract/ methods*

*The aim of the evaluation was twofold: i) to assess the effect of the service on mental wellbeing and primary health care resource use and ii) to assess the whether the service could be implemented as intended […] Primary health care resource use. Primary health care use data were collected electronically and anonymously from patient health care records. All GP referrals into the social prescribing scheme were flagged by a unique identification code. (19) – methods*

*Key outcomes of the reviewed studies revealed […] • Reduction in visits to general practitioners, referring health professionals and primary or secondary care services(4) – results*

*The economic evaluation method, analyses and models clients attendance at A&E and rate of emergency admissions to hospital.(71) – results*

*Data sources and variables. The analysis presented in this chapter is based on pseudonymised patient-level hospital episode data for Social Prescribing Service users provided by the NHS Data Management and Integration Centre (DMIC). Data linkage was made using the NHS numbers of Social Prescribing Service users provided by Voluntary Action Rotherham. Following exploratory analysis of all the data provided a series of outcome variables were created to provide the basis of the headline analysis presented in this report: The number of non-elective inpatient episodes (FCEs)10 in the 12 months before and after the first contact with Social Prescribing. The number of non-elective continuous inpatient spells 11 in the 12 months before and after the first contact with Social Prescribing. The number of bed days as a non-elective inpatient in the 12 months before and after the first contact with Social Prescribing. The number of Accident and Emergency attendances in the 12 months before and after the first contact with Social Prescribing. (84) – methods*

*Primary Care Utilisation. Information on the number of GP and other primary care consultations, number of consultations that were about psychosocial problems, number of prescriptions of psychotropic medication, and of mental health related referrals (i.e. to psychiatrists, psychologists, counsellors, community metal health teams) were obtained from patient medical records for the three month period prior to the date of referral to the Community Link service and for the three month period following the patient's first appointment with the GPCMHW (87) – methods*

*At the end of 2014, we performed a mixed-methods study to assess the impact of the Welzijn op Recept programme on healthcare costs, GP attendance rates, health outcomes and patient well-being.* (42) *– introduction*

*Bespoke self-efficacy items: A single item assessed all respondents’ confidence that they could manage their own health on a five point Likert scale where higher scores indicated higher confidence. A further five self-efficacy items were designed to assess respondents who had indicated that they had one or more of the four target long term conditions ability to manage their long term condition(s). These were also assessed on a five point Likert scale where higher scores were indicative of greater levels of confidence.* (48) *– methods*

*Changes in health service use. Health professionals in qualitative interviews reported that social prescribing reduced demand on primary care services (Involve North East, 2013). Other studies found that 82 per cent of patients decreased their number of health professional consultations (Popay et al., 2007) and there was a reduction in medical prescriptions (Age UK, 2012). However, Grant et al. (2000) reported little decease in primary care use. Cost-effectiveness. Social prescribing is promoted as potentially cost saving (NHS Tayside, 2011). However, the only cost-effectiveness analysis found social prescribing to be on average £20 more expensive per patient compared with usual care over a four month period (Grant et al., 2000)* (53) *– results*

*Importantly the SP project is also able to report on GP attendance and prescription data at one referring GP practice. Although the analysis of the prescription data is still on-going, preliminary results reveal a clear drop in GP attendance for most SP beneficiaries.(23) – results*

*The impact on the use of GP health care was measured in the 12 weeks prior to the social prescribing activity and for the 12 weeks of the activity. Where a patient failed to start the social prescribing activity, the analyses were dichotomised into the 12 weeks before and 12 weeks after the social prescribing referral date. Measured use ofGP health care included: patient visits to GP surgery, home visits by a GP and telephone calls to the GP surgery. The number of new repeat medications was recorded for the same 12-week periods. (24) – methods*

*Outcome measures included the number of GP appointments, prescriptions of psychotropic medications and the number of SCR. The ﬁnancial and environmental impacts were calculated for each outcome using national averages or accepted conversion factors (see Table 1). The exception to this was the ﬁnancial cost of medications, which was obtained from the British National Formulary (www.bnf.org). The cheapest cost for each medication was taken. Data regarding how frequently the patient was reviewed following an SCR were not available; therefore, it was assumed that the patient was seen the minimum number of times per referral – that is, once.* (50) *– methods*

*Primary: contact with primary health including measures such as frequency of GP consultations (either face-to-face or telephone) Secondary: changes in physical and/or mental health*(44) *– methods*

*Table 1 outlines the aims of the programmes described in the empirical studies. The stated aims were those listed in the individual studies, while the core aims were derived by grouping together similar aims across programmes. The core aims were then grouped in relation to the level at which the intervention was aimed: individual or system. The core individual aims identified included improved mental well-being, improved physical well-being, and improved social well-being. The core system-level aims included optimised health service use and decreased health service cost. Only nine studies stated a single aim. The majority of studies thus stated multiple aims: 16 stated two, 10 stated three, four stated four and one study stated five aims. Nineteen studies focused on both individual-level and system-level outcomes (see online supplementary appendix 2 for full details). Improved mental well-being was the most common core aim, with 25 of 41 studies. Physical wellbeing, social well-being, and optimised service use were also frequently cited with 16, 21 and 23 studies, respectively. Six studies addressed the least common core aim of cost savings(7) – results*

*It appears that Health Buddy* ***clients*** *interviewed for this evaluation gained the most benefit and anticipate needing to see their GPs less in the future. (68) – executive summary/results*

**Code 2.7. Lifestyle drift – references to “choice”, “empowerment”**

*The CHAT scheme illustrates how social prescribing can offer the opportunity to address social needs through individual consultations.(1) – results*

*Social prescribing interventions benefit patients by* ***supporting them*** *to address the wider psychosocial determinants of health, enabling better health‐condition management and the adoption of healthier behaviours (Mossabir, Morris, Kennedy, Blickem, & Rogers, 2015) (17) – introduction*

*In the last few years there has been an emergence of interventions focusing on the social component of care, such as social prescribing, art on prescription, exercise/physical activity on prescription, walking groups and the introduction of health trainers, with some evidence for behaviour change [2–4].* ***These aim to help people manage their chronic condition****, prevent more serious health problems developing, and contribute to addressing health inequalities by building social support networks. (19) – background*

*[…] a major element of social prescribing remains individual behaviour change through ‘empowerment’ of service users to make better choices [11]. (18) – introduction*

*social isolation, but it does mitigate the impact on individual health and enable people with social needs to make positive* ***choices****. (1) – results*

*For these patients, the PSS not only provided information, both verbally and through leaﬂets, but also offered advice about which services patients would ﬁnd the most useful. Wherever possible, attempts were made to present patients with referral* ***choices*** *[…] (21) – results*

*The PSS may also be seen as empowering patients to* ***take control*** *over their lives. For example, a recent factor analysis of empowerment in healthcare outlines components of care that are similar to those provided by the PSS [14], such as providing patients with information and* ***choices*** *concerning their future care. The concept of Empowerment has been empirically linked to learned mastery theory [15] whereby patients who are given control over their lives become increasingly motivated [16]. (21) – discussion*

*These programmes recognise the importance of social, economic and cultural factors on well‐being and provide Sefton residents with* ***a ‘menu’ of treatment options*** *that encourage levels of* ***self‐help****, personal responsibility and social and community engagement. (9) – introduction*

*A major focus of the work was to empower the patient to* ***make their own choices*** *and even if there was no further progression onto support in the community, having the space and time to explore the context* (35) *– results*

*The service* ***extended choice*** *for a wide range of patients, represented a viable alternative to CBT and medication and represented a suitable option for those experiencing isolation and frequent attenders.*(50) *– introduction*

*So link workers are skilled case workers with a great deal of local knowledge. Social prescribing* ***schemes have “menus” of support*** *activities often including specifically funded activities that are bought in to support patients. […] Secondly it brings the whole set of support mechanisms available in the wider community - and especially in voluntary and community sector - to the patient. Thirdly social prescribing schemes refine and improve the care* ***and support “market”.*** *As social prescribing schemes get bigger more referral is made to the more effective and popular ways of supporting people. So, over time,* ***the market for support and care*** *can thus be more streamlined to the needs and preferences of patients(86) – introduction*

*Link workers are trained in behaviour change methods, such as motivational interviewing techniques, that help service users identify which areas of their lives they wish to change and how. These techniques emphasis service users' choice and control over their decisions and behaviours [17]. (18) – introduction*

**Discourse 3. SP as enhancing personalised care in general practice**

**Code 3.1. Impersonal, rushed, stretched general practice (characterisation of general practice)**

*Service users reported that they engaged with further navigator appointments because they felt listened to and valued. In addition, service users reported that appointments with navigators felt less rushed and, unlike with GPs, they felt able to discuss their non-clinical needs without being pointed to a medical solution to deal with the consequences of the non-medical problems: “Social needs, mental needs, you know, just general back-up in life in general. I never felt that I had that before this [. . .] I was able to open up to [navigator]. [. . .] If you go to the GP and you say ‘I am having trouble at home’, they say ‘oh take this tablet or take this pill, oh you will be fine in a couple of weeks’, or you know, ‘see what it is like and come back in a week time if it is not better’. I am on enough tablets, the last thing I want is more tablets!” (Service user 2)(88) – results*

*“There’s a huge difference [between a link worker and a nurse or doctor]. The practice nurse just wants to stick the jab in your arm, and let them get on with it, and that’s it. Doesn’t ever really have time to do the in-depth analysis of where you’re at and what you’re doing. The ‘Ways to Wellness’ person has that concern. I don’t want people going home and having sleepless nights over me, but it’s nice to think that they do care, and I really feel that they do.” (P18, female, age 65–69) (18) – results*

*“I am stuck in this wheel chair and have a lot of problems. I knew that my GP just wanted to get rid of me out of the door. I knew she didn’t want to open up the can of worms that were in my head and forcing me to talk to the Samaritans.”* *(69) – results*

*The Link Worker role is pivotal to the success of this bridge and their role needs to be properly*

*resourced and understood. Failure to provide Link Workers with adequate training*

*and support could endanger both the Link Workers themselves and their patients. At its best, social prescribing can offer a uniquely tailored, truly person-centred intervention in an over-stretched health service, where patients or clients are given the space and time needed to explore their options and make sustainable behavioural changes in their lives.* (35) *c 3.2. and 3.1. - discussion*

***﻿****Primary care staff may feel overwhelmed and not equipped to handle the psychosocial problems that primary care patients present with [2, 3]. The commonly available options for patients presenting psychosocial problems are medication, psychotherapy (cognitive behavioural therapy), and counselling [4]. (89)- Introduction*

**Code 3.2. SP as able to deliver empathetic, person-cantered care (characterisation of SP)**

*“One of the main things about talking to them is they feel confident, and that gives you confidence. It makes you feel that all is not lost, that you will be fine and that you can do things. And every little problem gets ironed out. And it is very comforting to know that you are not by yourself, that you can ring someone” (CHA interview 1, and reiterated in her daily diary).*(37) *– results*

*Patients spoke positively of being given the time and space to discuss their needs, feeling heard, and the proactive focus of the intervention(74) – results*

*Clients highlighted the personal qualities that they valued “very genuine, very caring”, the closeness of the bond that had developed, the understanding and support that had been provided and how enjoyable the contact had been(71) – results*

*“[Link Worker] epitomises the word, wellbeing, because that’s what she’s got in mind for you, your wellbeing. Nothing else, she gives the impression that you’re the only one that she’s looking after, at that time, in that moment in time, you’re the only one that matters to her.” (P9, male, 55–59 years)* (32) *– results*

*Service users reported that they engaged with further navigator appointments because they felt listened to and valued. In addition, service users reported that appointments with navigators felt less rushed and, unlike with GPs, they felt able to discuss their non-clinical needs without being pointed to a medical solution to deal with the consequences of the non-medical problems: “Social needs, mental needs, you know, just general back-up in life in general. I never felt that I had that before this [. . .] I was able to open up to [navigator]. [. . .] If you go to the GP and you say ‘I am having trouble at home’, they say ‘oh take this tablet or take this pill, oh you will be fine in a couple of weeks’, or you know, ‘see what it is like and come back in a week time if it is not better’. I am on enough tablets, the last thing I want is more tablets!” (Service user 2)(88) C3.2. and 3.1. – results*

*“There’s a huge difference [between a link worker and a nurse or doctor]. The practice nurse just wants to stick the jab in your arm, and let them get on with it, and that’s it. Doesn’t ever really have time to do the in-depth analysis of where you’re at and what you’re doing. The ‘Ways to Wellness’ person has that concern. I don’t want people going home and having sleepless nights over me, but it’s nice to think that they do care, and I really feel that they do.” (P18, female, age 65–69) (18) – results*

*“ She gives you, ‘… give it a go,’ and she’ll explain… and if you don’t go she doesn’t get disappointed or anything like that, she just says, ‘Oh, right, well, we’ll sort something else out for you****.”*** *(P11, male, 45–49 years).* (32) *– results*

*One CLP reported being pleased at being able to help a particularly challenging patient group, in this case older men with addiction to alcohol, for whom few local services were available. The CLP reported that some of these men had started to engage with support after a long history of disengagement. When prompted about the likely cause for the reengagement: “I think it was the time given to them, it was the listening to them, empathising with their situation and being openly honest with them, but also being persistent and letting them know ‘no, I’m not going away, I’m going to be here to support you’.” CLP 4.* (45) *– results*

*The Link Worker role is pivotal to the success of this bridge and their role needs to be properly*

*resourced and understood. Failure to provide Link Workers with adequate training*

*and support could endanger both the Link Workers themselves and their patients. At its best, social prescribing can offer a uniquely tailored, truly person-centred intervention in an over-stretched health service, where patients or clients are given the space and time needed to explore their options and make sustainable behavioural changes in their lives.* (35) *- discussion*

*However, whilst the Arts on Prescription programme engages people with arts activities, it is the quality of the human relationships and the atmosphere that is created by the service providers that was of most significance to the participants. (81)- discussion*

**Code 3.3. Impact of empathetic, supportive, person-cantered care on adherence, behavioural change, satisfaction**

*In cases where patients had agreed to seek help from a community-based service, volunteers would seek their permission to make an appointment on their behalf. Five of the volunteers indicated that if appointments were not made during the consultation, there would be a strong doubt as to whether patients would make appointments on their own.(21) – results*

*The original rationale for the Programme was that if individuals feel supported in their lives, they will be more likely to respond to information on ways to improve their health and to live well.*(45) *– methods*

*Link workers paced the level of support they offered, particularly in the initial stages: “I just expected the Link Worker to introduce me to the gym, and that would have been it. And I think, if it had just been [that] I would have turned round, and I would have gone the opposite direction. But because of the way it was so gradually and really professionally linked in to different things, I just felt as though I’d floated into it, rather than getting shoved from behind. I just felt as though I was gradually moved into it.” (P2, female, 70–74 years)*(32) *– results*

*“I actually got first health referral and I didn’t go … ya know when you don’t talk to people and that you can’t make yourself go anywhere on your own. So anyway this time … [health trainer] met me, took me to [sports centre], took me to the gym, took me to meet the [swimming instructors] and … now they’ve given me that confidence, he didn’t have to take me every day, he only had to take me that once and I’m getting round more and more … like I say I’m meeting people in the street now what go to the social café and ‘How do! All right!’ and who knows I can spend ten, fifteen minutes chatting to them …”(79) – results*

*Service users identified the meetings with navigators and the establishment of a supportive and trust relationship as major enablers to behaviour change.(14) – results*

*The supportive function of the feedback on structured exercising was particularly highlighted by participants. Five of the eight male participants were thrilled by the possibility of being able to compete with the rowing machine. The ‘‘objective’’ information given by the machine, reporting distance, time or just burned kilojoules, had the effect of an external feedback. The physiotherapist was given the role of a strict (‘‘tough – but caring’’) parent, while the group members became encouraging siblings. ‘‘Most of us don’t have the backbone to exercise on our own and we will stay home if it is raining. But knowing that four idiots will give you a telling-off if you didn’t show up – well, that’s enough to make me show up’’ (male, 61 years).*(61) *– results*

*By follow‐up, link workers’ experiences supported the contention that simply signposting to activities (the principle underlying ‘light’ social prescribing (Kimberlee, 2015)) would be ineffective in engaging clients and much more intensive support was required: “The work that we do is quite in‐depth with the client … Some people say, “Well we should just be signposting and that's it.” But actually we know that our clients, if we did that, they're not going to engage … So really we are quite intense. (P2, FG1, Phase 2)” – results*

*However, over the period of engagement, some participants expressed negative views primarily due to personnel changes among link workers that resulted in lost continuity. These accounts highlighted the importance of the link worker/service user relationship and how changes to this highly-valued and often therapeutic relationship could be upsetting and lead to disengagement: “Well, I don't get as much support now. My first worker left, I used to see her a lot. I was put onto another one, who I've only seen about two or three times. Now she’s left and they’ve put me onto somebody else who I've never seen or been contacted by. I feel a bit let down because my first one was brilliant. She was on the phone, talking, we used to meet up and it was great. I just feel as though I've been let down now…I just feel as though I've been pushed to one side. I don't know what’s going on with the leaving and stuff like that. I just can't understand why the new one hasn't phoned up to introduce herself to me.” (P4, female, 55-59). (18) – results*

*Some participants suggested that having their measurements taken by the AL advisor at consultation, such as blood pressure and body weight, helped them to stay motivated, and that without this they would not have stuck to their goals: "It makes you feel more positive about it, when you've got somebody there. If you could see someone regularly you know you're going on the scales, because if you try to diet for yourself you just cheat and you're only cheating yourself."*(54) *– results*

*Underpinning the above was a belief in the importance of networks to facilitate and increase the likelihood of a successful social prescription, with the assumption that the converse would be true: patients who are simply given information about an opportunity will not necessarily take it up without some hand‐holding’ (Brandling & House, 2009). Thus, ‘having someone to encourage or support’ (ERS Research & Consultancy, 2013) was considered central to successful referrals.*(39) *– results*

*Service users emphasised that feeling supported from service providers was a key factor determining their adherence to onward referrals. When service users haven’t received a response from service providers, they didn’t engage with referred services(88) – results*

*The lack of a follow-up session could be a reason to drop out, as shown in the following example (this woman did not return to the activity): “I missed the so-called ‘big stick’. It was hard for me to attend the activity the ﬁrst time. I went, but people did not say much to me and that made it harder for me to go back the next time. That’s a problem for me. The wellbeing coach called me to ask how I felt, but in the meantime, my problems got worse. The wellbeing coach told me on the phone to look on the internet for other options. But going there alone is difﬁcult. That is not funny to say. I know I can look it up and that they can make an appointment for me. They suggested asking my neighbour to accompany me to the activity, but … to ask something like that … it’s difﬁcult. I feel like I cannot do that by myself.” (Interview 2)*(42) *– results*

*“Yes, so I knew someone was waiting for me [. . .] Yes, I would just sit and waited at the table, and she picked me up.” (Service user 1) Ongoing support and motivation, especially during physical activities, were identified as another factor promoting service user adherence to the SP programme.(88) – results*

*One CLP reported being pleased at being able to help a particularly challenging patient group, in this case older men with addiction to alcohol, for whom few local services were available. The CLP reported that some of these men had started to engage with support after a long history of disengagement. When prompted about the likely cause for the reengagement: “I think it was the time given to them, it was the listening to them, empathising with their situation and being openly honest with them, but also being persistent and letting them know ‘no, I’m not going away, I’m going to be here to support you’.” CLP 4,* (45) *– results*

*Although current PSS policy suggests that patients should not be followed up, there seem to be many advantages to this strategy. For example, follow up appointments would: (a) aid the identiﬁcation of patients whose initial referrals have collapsed; (b) provide feedback to volunteers as to the appropriateness of a referral for a given patient problem thus assisting them in managing similar cases in the future; (c) support the PSS in dealing with patients with multiple problems without feeling pressured to manage these in a single session; and (d) patients would not be required to make a GP or nurse appointment simply to be re-referred to the PSS, thus reducing the workload on these resources.(21) – discussion*

**Code 3.4. Patients as individuals with enduring and complex health issues in need on ongoing care (characterisation of SP users)**

*“I found it very interesting that people with long-term health issues didn’t feel they needed or wanted a huge solution – no one asked for a cure! What they did want was smaller and more supportive outcomes. It’s the small things that mean the most”* (33) *– results*

*All clients feel that it would help to have a longer period of interaction with the scheme.(68) – executive summary/results*

*Unlike counsellors, where there is often a limit to how many times a patient can see them, this patient felt that the CLP was there if support was needed again. […] There was something about the un-conditionality and continuity of support from CLPs that was valued by patients.* (45) *– results*

*Participants appreciated the flexibility and ‘open door’ nature of Ways to Wellness, although this could be limited for those who were working. Of particular value was the potential to be engaged with the service for up to 2 years. Due to the long-term and complex nature of conditions which often fluctuated, participants recognised that a shorter-term approach would be inadequate, particularly when accounting for wider life events such as job loss or bereavement: “Well, I think you can dip in and dip out. It’s the kind of thing if you need them, you phone them and they’ll get straight back to you. They’re there, I know they’re there … if something happens to me now.” (P13, male, 60–64 years)*(32) *– results*

*Following this bridging, however, participants found it valuable to know that support was still there, in the background, for when it was needed(79) – results*

*While some participants felt they would be content with two years’ contact, others facing particular challenges (for example, poor mental health or homelessness) wished to continue long-term with the social prescribing programme, feeling “I will always need somebody to help me” (P16, female age 65–69). The fluctuating and chronic nature of LTCs resulted in an almost universal feeling that the opportunity to re-contact their link worker and, if needed, re-enter the programme “for a second bite of the cherry” (P18, female, age 65–69) would be desirable: “I mean they can extend it. I mean it depends what people’s needs are at the time I suppose. I mean with me, I’d still want to be in contact somewhere along the line, which I think they will do. If something happened to me, if I had an angina attack or something I think I would need them full time all the time then. I know if I have a bad angina attack I’m not going to recover that well.” (P12, male, age 55–59) (18) – results*

*If necessary, and acceptable to the client, long-term follow-up support was arranged and a personal wellbeing plan completed.*(37) *– methods*

*In cases where patients had agreed to seek help from a community-based service, volunteers would seek their permission to make an appointment on their behalf. Five of the volunteers indicated that if appointments were not made during the consultation, there would be a strong doubt as to whether patients would make appointments on their own.(21) – results*

*The level of support that some service users required in order to engage with services, particularly those involving physical activity, appeared to be considerable. – results*

*“[We are]* ***support workers more than link workers*** *… I think you find when you go in with a client and they've got massive problems, like they've got no money for food, you can't just say, ‘Do you fancy going to the gym?’ We have to look at the problem that's affecting them at the moment. (P2, FG4, Phase 2)”(17) – results*

*Whilst initial expectations for some Link Workers were of the work being a predominantly signposting role, it soon became clear that for many patients a more intensive approach was necessary. […] “the service was very much in development as we were delivering, you know, so we didn’t have a really, really clear model at the beginning so we were ﬁnding our feet a bit and maybe at the beginning it was more containable you know to be about this is about signposting, but as we started to work with people you realise that actually they need more time and more input really. (LW1, Scheme 2)*(35) *– results*

*One CLP reported being pleased at being able to help a particularly challenging patient group, in this case older men with addiction to alcohol, for whom few local services were available. The CLP reported that some of these men had started to engage with support after a long history of disengagement. When prompted about the likely cause for the reengagement: “I think it was the time given to them, it was the listening to them, empathising with their situation and being openly honest with them, but also being persistent and letting them know ‘no, I’m not going away, I’m going to be here to support you’.” CLP 4,* (45) *– results*

*“And also the complex nature of… Or the complex issues that the people face cannot be solved overnight. And sometimes you need kind of more intensive work. You need to work with them for over…” CLP 5,* (45) *– results*

*Although another CLP suggested that it takes time to develop relationships because of* ***people’s complex problems*** *which did not reduce attendance but is worthwhile: “it took time, you know, to build up that relationship with the individual, but you can see just the difference it's made, you know, he knows I'm there and you know I guess it's like chiselling away, each time that I see him, you know, he'll tell me something else” CLP 8,* (45) *– results*

*“The time wasn’t really enough. I wish the time was a bit extended. I requested more time. It’s not enough time to sort out everything that a person would actually want to do. What I wanted we couldn’t really sort out everything.”* (49) *– results*

*A number of interviewees, however, stated that it would be useful to have a greater number of one to one sessions should they need to: “I think it probably could have been longer. I think it should be more like help until they think they are done. Cos when I first met her I was really down, but towards the end I was much better but I still could have done with one or two more.” (Male client: interview 4, aged under 50 years. Referred to the social prescribing service by GP)* (49) *– results*

*One interesting finding from the discussion was that many of the participants felt that the service should be ongoing, and not limited to a certain number of consultations or a specific time period. While very few had needed six consultations, they were not in favour of the service being limited and being unable to see the AL advisor for 'check-ups': "But after the sessions I wouldn't like to think that it is finished full stop, and that you're in the filing cabinet. I would like to think that you could go at least twice a year ... as you would to a doctor for a check-up. To go back to 'Sarah' just to see whether you had lapsed in anything, if you have forgotten anything, or if there is anything new on the market so to speak." […]*(54) *– results*

*While not all participants were asked about this issue, as it only arose during one discussion group, those who were asked felt very strongly that the service and support should be ongoing. This issue should be explored and the implications of offering the service on a continuing basis should be identified. While on the one hand the participants clearly felt that this was important, and that without it they might fall back into old habits, there is also a need to encourage people to take responsibility for themselves and their health, and not become too dependent on health staff or services. This issue has been previously raised by Hunt and Hillsdon [18]. Furthermore, if participants were able to continue seeing the AL advisor it would increase demand and time pressure on an already stretched service.* (54) *– results/discussion*

*The evidence examined here indicates the level of complexity necessary for robust implementation, and so services need to better understand what it is that patients need in terms of complex care. Signposting at the point of presentation for individuals with mental health needs, for example, is not likely to be sufficient.* (39) *– discussion*

**References:**

1. South J, Higgins TJ, Woodall J, White SM, James; W. Can social prescribing provide the missing link? Prim Heal Care Res Dev [Internet]. 2008 Oct 4 [cited 2018 Jun 17];9(4):310–8. Available from: http://www.journals.cambridge.org/abstract_S146342360800087X

2. Swift M. People powered primary care: Learning from Halton. J Integr Care [Internet]. 2017;25(3):162–73. Available from: https://www.scopus.com/inward/record.uri?eid=2-s2.0-85021350070&doi=10.1108%2FJICA-12-2016-0050&partnerID=40&md5=d8743df21cf5af5746b0ed962c9ee9e1

3. Bungay H, Clift S. Arts on Prescription: A review of practice in the UK. Perspect Public Health [Internet]. 2010;130(6):277–81. Available from: file:///Users/sarakalde/Library/Application Support/Mendeley Desktop/Downloaded/Bungay, Clift - 2010 - Arts on Prescription A review of practice in the UK.pdf

4. Chatterjee HJ, Camic PM, Lockyer B, Thomson LJM. Non-clinical community interventions: a systematised review of social prescribing schemes. Arts Health [Internet]. 2018 May 4 [cited 2019 Jun 20];10(2):97–123. Available from: https://www.tandfonline.com/doi/full/10.1080/17533015.2017.1334002

5. Ferguson K, Hogarth S. Social prescribing in Tower Hamlets: evaluation of borough-wide roll-out [Internet]. London, UK: University Collegue London; 2018. Available from: file:///Users/sarakalde/Library/Application Support/Mendeley Desktop/Downloaded/Ferguson, Hogarth - 2018 - Social prescribing in Tower Hamlets evaluation of borough-wide roll-out.pdf

6. Mercer SW, Fitzpatrick B, Grant L, Chng NR, McConnachie A, Bakhshi A, et al. Effectiveness of Community-Links Practitioners in Areas of High Socioeconomic Deprivation. Ann Fam Med [Internet]. 2019 Nov 11;17(6):518–25. Available from: http://www.annfammed.org/lookup/doi/10.1370/afm.2429

7. Rempel ES, Wilson EN, Durrant H, Barnett J. Preparing the prescription: a review of the aim and measurement of social referral programmes. BMJ Open [Internet]. 2017 Oct 12 [cited 2018 Jun 12];7(10):e017734. Available from: file:///Users/sarakalde/Library/Application Support/Mendeley Desktop/Downloaded/Rempel et al. - 2017 - Preparing the prescription a review of the aim and measurement of social referral programmes(2).pdf

8. Wallace C, Elliott M, Thomas S, Davies-McIntosh E, Beese S, Roberts G, et al. Using consensus methods to develop a Social Prescribing Learning Needs Framework for practitioners in Wales. Perspect Public Health [Internet]. 2020 Jan 28;175791391989794. Available from: http://journals.sagepub.com/doi/10.1177/1757913919897946

9. Creative Alternatives. Arts on prescription in Sefton. Programme Report December 2009 [Internet]. [Netherton]: Creative Alternatives; 2009. Available from: http://www.artsforhealth.org/resources/CA Report 2009.pdf

10. Aggar C, Thomas T, Gordon C, Bloomfield J, Baker J. Social Prescribing for Individuals Living with Mental Illness in an Australian Community Setting: A Pilot Study. Community Ment Health J [Internet]. 2020 May 13; Available from: http://link.springer.com/10.1007/s10597-020-00631-6

11. Bragg R, Leck C. Good practice in social prescribing for mental health: the role of nature-based interventions. [Internet]. Natural England Commissioned Report. [York]: Natural England; 2017. Available from: http://publications.naturalengland.org.uk/publication/5134438692814848

12. Whitelaw S, Thirlwall C, Morrison A, Osborne J, Tattum L, Walker S. Developing and implementing a social prescribing initiative in primary care: insights into the possibility of normalisation and sustainability from a UK case study. Prim Heal Care Res Dev [Internet]. 2017 Mar 8 [cited 2019 Jun 21];18(2):112–21. Available from: http://dx.doi.org/10.1017/S1463423616000219

13. Kok M, Solomon-Moore E, Greaves C, Smith J, Kimberlee R, Jones M. Evaluation of Living Well, Taking Control: a community-based diabetes prevention and management programme [Internet]. [Bristol]: University of The West of England; 2016. Available from: http://eprints.uwe.ac.uk/30234/13/LWTC Evaluation Report_finalISBN.pdf

14. Pescheny J V, Randhawa G, Pappas Y. The impact of social prescribing services on service users: a systematic review of the evidence. Eur J Public Health [Internet]. 2020 Aug 1;30(4):664–73. Available from: http://dx.doi.org/10.1093/eurpub/ckz078

15. Skivington K, Smith M, Chng NR, Mackenzie M, Wyke S, Mercer SW. Delivering a primary care-based social prescribing initiative: a qualitative study of the benefits and challenges. Br J Gen Pr [Internet]. 2018 Jul 1 [cited 2019 Feb 2];68(672):e487–94. Available from: http://dx.doi.org/10.3399/bjgp18X696617

16. Pescheny JV, Gunn LH, Randhawa G, Pappas Y. The impact of the Luton social prescribing programme on energy expenditure: a quantitative before-and-after study. BMJ Open [Internet]. 2019;9(6):e026862–e026862. Available from: http://dx.doi.org/10.1136/bmjopen-2018-026862

17. Wildman JM, Moffatt S, Penn L, O’Brien N, Steer M, Hill C. Link workers’ perspectives on factors enabling and preventing client engagement with social prescribing. Health Soc Care Community [Internet]. 2019 Jul 14;27(4):991–8. Available from: http://dx.doi.org/10.1111/hsc.12716

18. Wildman JM, Moffatt S, Steer M, Laing K, Penn L, O’Brien N. Service-users’ perspectives of link worker social prescribing: a qualitative follow-up study. BMC Public Health [Internet]. 2019 Dec 22 [cited 2019 Jun 21];19(1):98. Available from: https://doi.org/10.1186/s12889-018-6349-x

19. Carnes D, Sohanpal R, Frostick C, Hull S, Mathur R, Netuveli G, et al. The impact of a social prescribing service on patients in primary care: a mixed methods evaluation. BMC Heal Serv Res [Internet]. 2017 Dec 19 [cited 2018 Jun 4];17(1):835. Available from: http://dx.doi.org/10.1186/s12913-017-2778-y

20. Blickem C, Kennedy A, Vassilev I, Morris R, Brooks H, Jariwala P, et al. Linking people with long-term health conditions to healthy community activities: development of Patient-Led Assessment for Network Support (PLANS). Heal Expect [Internet]. 2013;16(3):e48-59. Available from: http://dx.doi.org/10.1111/hex.12088

21. Faulkner M. Supporting the psychosocial needs of patients in general practice: the role of a voluntary referral service. Patient Educ Couns [Internet]. 2004 Jan 1 [cited 2019 Jun 21];52(1):41–6. Available from: https://www.sciencedirect.com/science/article/pii/S0738399102002471?via%3Dihub

22. Jensen A. Culture Vitamins – an Arts on Prescription project in Denmark. Perspect Public Health [Internet]. 2019 May 8;139(3):131–6. Available from: http://journals.sagepub.com/doi/10.1177/1757913919836145

23. Kimberlee RH. Developing a Social Prescribing approach for Bristol [Internet]. Bristol: University of the West of England; 2013. Available from: http://eprints.uwe.ac.uk/23221/1/Social Prescribing Report-final.pdf

24. Loftus AM, McCauley F, McCarron MO. Impact of social prescribing on general practice workload and polypharmacy. Public Health [Internet]. 2017 Jul 1;148:96–101. Available from: file:///Users/sarakalde/Library/Application Support/Mendeley Desktop/Downloaded/Loftus, McCauley, McCarron - 2017 - Impact of social prescribing on general practice workload and polypharmacy.pdf

25. Tierney S, Wong G, Roberts N, Boylan A-M, Park S, Abrams R, et al. Supporting social prescribing in primary care by linking people to local assets: a realist review. BMC Med [Internet]. 2020 Dec 13;18(1):49. Available from: https://bmcmedicine.biomedcentral.com/articles/10.1186/s12916-020-1510-7

26. White JM, Cornish F, Kerr S. Front-line perspectives on ‘joined-up’ working relationships: a qualitative study of social prescribing in the west of Scotland. Health Soc Care Community [Internet]. 2017 Jan;25(1):194–203. Available from: http://dx.doi.org/10.1111/hsc.12290

27. Stickley T, Eades M. Arts on prescription: a qualitative outcomes study. Public Health [Internet]. 2013;127(8):727–34. Available from: http://dx.doi.org/10.1016/j.puhe.2013.05.001

28. Thomson LJ, Lockyer B, Camic PM, Chatterjee HJ. Effects of a museum-based social prescription intervention on quantitative measures of psychological wellbeing in older adults. Perspect Public Heal [Internet]. 2018;138(1):28–38. Available from: http://dx.doi.org/10.1177/1757913917737563

29. Dayson C, Bashir N, Pearson S. From dependence to independence: emerging lessons from the Rotherham Social Prescribing Pilot [Internet]. Sheffield: Centre for Regional Economic and Social Research. Sheffield Hallam University; 2013 [cited 2018 Jun 8]. Available from: http://www.instituteofhealthequity.org/file-manager/PDFs/from-dependence-to-independence-emerging-lessons-from-the-rotherham-social-prescribing-pilot-summary-report.pdf

30. Bertotti M, Frostick C, Hutt P, Sohanpal R, Carnes D. A realist evaluation of social prescribing: an exploration into the context and mechanisms underpinning a pathway linking primary care with the voluntary sector. Prim Heal Care Res Dev [Internet]. 2018 [cited 2018 Jun 26];19(3):232–45. Available from: http://dx.doi.org/10.1017/S1463423617000706

31. Coan J. Social prescribing at the Bromley by Bow Centre: annual report. April 2015 - March 2016. [Internet]. London: Bromley by Bow Centre,; 2016. Available from: file:///Users/sarakalde/Library/Application Support/Mendeley Desktop/Downloaded/Coan - 2016 - Social prescribing at the Bromley by Bow Centre annual report April 2015 - March 2016.pdf

32. Moffatt S, Steer M, Lawson S, Penn L, O’Brien N. Link Worker social prescribing to improve health and well-being for people with long-term conditions: qualitative study of service user perceptions. BMJ Open [Internet]. 2017 Jul 16 [cited 2018 Jun 4];7(7):e015203. Available from: http://dx.doi.org/10.1136/bmjopen-2016-015203

33. Chesterman D, Bray M. Report on some action research in the implementation of social prescription in Crawley. Paths to greater wellbeing: ‘sometimes you have to be in it to get it.’ Action Learn Res Pract [Internet]. 2018;15(2):168–81. Available from: https://www.scopus.com/inward/record.uri?eid=2-s2.0-85047840904&doi=10.1080%2F14767333.2018.1467302&partnerID=40&md5=40379f0bdae94c79daa1775e5d8aa141

34. Elston J, Gradinger F, Asthana S, Lilley-Woolnough C, Wroe S, Harman H, et al. Does a social prescribing ‘holistic’ link-worker for older people with complex, multimorbidity improve well-being and frailty and reduce health and social care use and costs? A 12-month before-and-after evaluation. Prim Health Care Res Dev [Internet]. 2019 Sep 24;20:e135. Available from: https://www.cambridge.org/core/product/identifier/S1463423619000598/type/journal_article

35. Frostick C, Bertotti M. The frontline of social prescribing – How do we ensure Link Workers can work safely and effectively within primary care? Chronic Illn [Internet]. 2019 Oct 17;174239531988206. Available from: http://journals.sagepub.com/doi/10.1177/1742395319882068

36. Stickley T, Hui A. Social prescribing through arts on prescription in a UK city: Referrers’ perspectives (part 2). Public Health [Internet]. 2012 Jul;126(7):580–6. Available from: https://linkinghub.elsevier.com/retrieve/pii/S0033350612001370

37. Beech R, Ong BN, Jones S, Edwards V. Delivering person-centred holistic care for older people. Qual Ageing Older Adults [Internet]. 2017;18(2):157–67. Available from: file:///Users/sarakalde/Library/Application Support/Mendeley Desktop/Downloaded/Beech et al. - 2017 - Delivering person-centred holistic care for older people.pdf

38. Kellezi B, Wakefield JRH, Stevenson C, McNamara N, Mair E, Bowe M, et al. The social cure of social prescribing: a mixed-methods study on the benefits of social connectedness on quality and effectiveness of care provision. BMJ Open [Internet]. 2019 Nov 14;9(11):e033137. Available from: http://bmjopen.bmj.com/lookup/doi/10.1136/bmjopen-2019-033137

39. Husk K, Blockley K, Lovell R, Bethel A, Lang I, Byng R, et al. What approaches to social prescribing work, for whom, and in what circumstances? A realist review. Health Soc Care Community [Internet]. 2020 Mar 9;28(2):309–24. Available from: https://onlinelibrary.wiley.com/doi/abs/10.1111/hsc.12839

40. Southby K, Gamsu M. Factors affecting general practice collaboration with voluntary and community sector organisations. Heal Soc Care Community [Internet]. 2018;26(3):e360–9. Available from: http://dx.doi.org/10.1111/hsc.12538

41. Dayson C. Evaluating social innovations and their contribution to social value: The benefits of a “blended value” approach. Policy Polit [Internet]. 2017;45(3):395–411. Available from: https://www.scopus.com/inward/record.uri?eid=2-s2.0-85021826832&doi=10.1332%2F030557316X14564838832035&partnerID=40&md5=184467db08d9bcd4ea330c220d3760f9

42. Heijnders ML, Meijs JJ. “Welzijn op Recept” (Social Prescribing): A helping hand in re-establishing social contacts-an explorative qualitative study. Prim Heal Care Res Dev. 2018;19(3).

43. Hamilton-West K, Gadsby E, Zaremba N, Jaswal S. Evaluability assessments as an approach to examining social prescribing. Heal Soc Care Community [Internet]. 2019;27(4):1085–94. Available from: http://dx.doi.org/10.1111/hsc.12726

44. Public Health England. Effectiveness of social prescribing - An evidence synthesis [Internet]. [London]: Public Health England; 2019 [cited 2019 Sep 29]. Available from: www.facebook.com/PublicHealthEngland

45. Mercer S. Evaluation of the Glasgow “Deep End” Links Worker Programme [Internet]. [Edinburgh]: NHS Health Scotland; 2017. Available from: http://www.healthscotland.com/uploads/documents/29438-1. Evaluation of the Glasgow “Deep End” Links Worker Programme - May 2017 - English.pdf

46. Mills H, Crone D, James DVB, Johnston LH. Exploring the Perceptions of Success in an Exercise Referral Scheme: a mixed method investigation. Eval Rev [Internet]. 2012 Dec 2;36(6):407–29. Available from: http://journals.sagepub.com/doi/10.1177/0193841X12474452

47. Redmond M, Sumner RC, Crone DM, Hughes S. Light in dark places: exploring qualitative data from a longitudinal study using creative arts as a form of social prescribing. Arts Heal [Internet]. 2018;1–14. Available from: http://dx.doi.org/10.1080/17533015.2018.1490786

48. NHS Leeds West CCG. Patient Empowerment Project Final Evaluation Report [Internet]. [Leeds]: NHS Leeds West Clinical Commissioning Group; 2016. Available from: https://www.leedsccg.nhs.uk/content/uploads/2018/05/Patient-Empowerment-Project-Leeds-West-Final-Report.pdf

49. Woodall J, Trigwell J, Bunyan A-MM, Raine G, Eaton V, Davis J, et al. Understanding the effectiveness and mechanisms of a social prescribing service: a mixed method analysis. BMC Heal Serv Res [Internet]. 2018;18(1):604. Available from: http://dx.doi.org/10.1186/s12913-018-3437-7

50. Maughan DL, Patel A, Parveen T, Braithwaite I, Cook J, Lillywhite R, et al. Primary-care-based social prescribing for mental health: an analysis of financial and environmental sustainability. Prim Heal Care Res Dev [Internet]. 2016;17(2):114–21. Available from: http://dx.doi.org/10.1017/S1463423615000328

51. Beardmore A. Working in social prescribing services: a qualitative study. J Health Organ Manag [Internet]. 2019 Nov 4;34(1):40–52. Available from: https://www.emerald.com/insight/content/doi/10.1108/JHOM-02-2019-0050/full/html

52. Kimberlee R. What is social prescribing? Adv Soc Sci Res J [Internet]. 2015 Jan 25 [cited 2018 Jun 10];2(1). Available from: http://scholarpublishing.org/index.php/assrj/article/view/808

53. Kilgarriff-Foster A, O’Cathain A. Exploring the components and impact of social prescribing. J Public Ment Health [Internet]. 2015 Sep 21 [cited 2018 Jun 10];14(3):127–34. Available from: http://eprints.whiterose.ac.uk/96464/

54. Wormald H, Waters H, Sleap M, Ingle L. Participants’ perceptions of a lifestyle approach to promoting physical activity: targeting deprived communities in Kingston-Upon-Hull. BMC Public Health [Internet]. 2006 Dec 4;6(1):202. Available from: https://bmcpublichealth.biomedcentral.com/articles/10.1186/1471-2458-6-202

55. Jones M, Kimberlee R, Deave T. South West Well-being Programme: Final Evaluation Report. [Internet]. Bristol; 2009. Available from: http://hsc.uwe.ac.uk/net/research/Data/Sites/1/UWE-SWWB-Report-WebVersion.pdf

56. Edmunds J, Ntoumanis N, Duda JL. Adherence and well-being in overweight and obese patients referred to an exercise on prescription scheme: A self-determination theory perspective. Psychol Sport Exerc [Internet]. 2007 Sep;8(5):722–40. Available from: https://linkinghub.elsevier.com/retrieve/pii/S146902920600077X

57. Hanlon P, Gray CM, Chng NR, Mercer SW. Does Self-Determination Theory help explain the impact of social prescribing? A qualitative analysis of patients’ experiences of the Glasgow ‘Deep-End’ Community Links Worker Intervention. Chronic Illn [Internet]. 2019 May 3;17(3):174239531984542. Available from: http://dx.doi.org/10.1177/1742395319845427

58. Morton KL, Biddle SJH, Beauchamp MR. Changes in self-determination during an exercise referral scheme. Public Health [Internet]. 2008 Nov;122(11):1257–60. Available from: https://linkinghub.elsevier.com/retrieve/pii/S003335060700371X

59. Rahman RJ, Thogersen-Ntoumani C, Thatcher J, Doust J. Changes in need satisfaction and motivation orientation as predictors of psychological and behavioural outcomes in exercise referral. Psychol Health [Internet]. 2011 Nov;26(11):1521–39. Available from: http://www.tandfonline.com/doi/abs/10.1080/08870446.2010.538849

60. Rouse PC, Ntoumanis N, Duda JL, Jolly K, Williams GC. In the beginning: Role of autonomy support on the motivation, mental health and intentions of participants entering an exercise referral scheme. Psychol Health [Internet]. 2011 Jun;26(6):729–49. Available from: http://www.tandfonline.com/doi/abs/10.1080/08870446.2010.492454

61. Roessler KK. A corrective emotional experience - or just a bit of exercise? The relevance of interpersonal learning in Exercise on prescription. Scand J Psychol [Internet]. 2011 Aug;52(4):354–60. Available from: http://doi.wiley.com/10.1111/j.

62. Bickerdike L, Booth A, Wilson PM, Farley K, Wright K, Wilson PM. Social prescribing: less rhetoric and more reality. A systematic review of the evidence. BMJ Open [Internet]. 2017 [cited 2018 Jun 2];7(4):e013384. Available from: file:///Users/sarakalde/Library/Application Support/Mendeley Desktop/Downloaded/Bickerdike et al. - 2017 - Social prescribing less rhetoric and more reality. A systematic review of the evidence(2).pdf

63. Dayson C, Hogarth S. Evaluation of HALE Community Connectors Social Prescribing Service [Internet]. Sheffield : Sheffield Hallam University; 2018. Available from: file:///Users/sarakalde/Library/Application Support/Mendeley Desktop/Downloaded/Dayson, Hogarth - 2018 - Evaluation of HALE Community Connectors Social Prescribing Service 2017.pdf

64. Wigfield A, Alden S, Erika K. Age UK’s fit for the future ‘Social Prescribing’ extension project: evaluation report. [Internet]. Leeds: University of Leeds; 2015. Available from: file:///Users/sarakalde/Library/Application Support/Mendeley Desktop/Downloaded/Wigfield, Alden, Erika - 2015 - Age UK’s fit for the future ‘Social Prescribing’ extension project evaluation report.pdf

65. Centre for Reviews and Dissemination. Evidence to inform the commissioning of social prescribing [Internet]. Evidence briefing. York: University of York; 2015 [cited 2018 Jun 2]. Available from: file:///Users/sarakalde/Library/Application Support/Mendeley Desktop/Downloaded/Unknown - 2015 - Evidence to inform the commissioning of social prescribing.pdf

66. Age UK. Social prescribing: A model for partnership working between primary care and the voluntary sector. York: Age UK; 2018.

67. Sumner RC, Crone DM, Baker C, Hughes S, Loughren EA, James DVB. Factors associated with attendance, engagement and wellbeing change in an arts on prescription intervention. J public Heal [Internet]. 2019 Apr 8; Available from: http://dx.doi.org/10.1093/pubmed/fdz032

68. Baines A. Rugby Social Prescribing Project ConnectWELL. Harnessing community capacity to improve health and wellbeing. Vol. 151. Coventry: Roundberry Projects; 2015.

69. Kimberlee RH. Gloucestershire Clinical Commissioning Group’s Social Prescribing Service: Evaluation Report [Internet]. Bristol: University of the West of England; 2016. Available from: http://www.periphery.co.uk/joyn

70. ERS Research and Consultancy. Newcastle Social Prescribing Project. Final Report. [Newcastle upon Tyre]: ERS Research and Consultancy; 2013.

71. Darnton P, Liles A, Sladen J, Benson T, Lawford N. Independent evaluation of the North East Hampshire and Farnham Vanguard. Making Connections service [Internet]. North East Hampshire and Farnham Vanguard evaluation reports. Southampton: Wessex AHSN; 2018. Available from: http://www.northeasthampshireandfarnhamccg.nhs.uk/about-the-ccg/happy-healthy-at-home/achievements

72. Thomson LJ, Camic PM, Chatterjee HJ. Social prescribing: a review of community referral schemes [Internet]. London: UCL; 2015. Available from: file:///Users/sarakalde/Library/Application Support/Mendeley Desktop/Downloaded/Thomson, Camic, Chatterjee - 2015 - Social prescribing a review of community referral schemes.pdf

73. R Outcomes. Social prescribing in Wessex: understanding its impact and supporting spread [Internet]. Southampton: Wessex Academic Health Science Network; 2017. Available from: http://wessexahsn.org.uk/projects/222/summary-evaluation-reports-and-papers

74. Bertotti M et al, City and Hackney Clinical Commissioning Group. Shine 2014 final report. Social Prescribing. Integrating GP and community assets for Health. [Internet]. London: The Health Foundation; 2015 [cited 2018 Jun 8]. Available from: www.health.org.uk

75. Grant C. A randomised controlled trial and economic evaluation of a referrals facilitator between primary care and the voluntary sector. BMJ [Internet]. 2000 Feb 12;320(7232):419–23. Available from: http://www.bmj.com/cgi/doi/10.1136/bmj.320.7232.419

76. Jensen A, Stickley T, Torrissen W, Stigmar K. Arts on prescription in Scandinavia: a review of current practice and future possibilities. Perspect Public Health [Internet]. 2017;137(5):268–74. Available from: file:///Volumes/TOSHIBA EXT/BIBLIO MENDELEY/BIBLIO MENDELAY/Jensen et al. - Perspectives in public health - 2017.pdf

77. Jones F, Harris P, Waller H, Coggins A. Adherence to an exercise prescription scheme: The role of expectations, self-efficacy, stage of change and psychological well-being. Br J Health Psychol [Internet]. 2005 Sep;10(3):359–78. Available from: http://doi.wiley.com/10.1348/135910704X24798

78. Duda JL, Williams GC, Ntoumanis N, Daley A, Eves FF, Mutrie N, et al. Effects of a standard provision versus an autonomy supportive exercise referral programme on physical activity, quality of life and well-being indicators: a cluster randomised controlled trial. Int J Behav Nutr Phys Act [Internet]. 2014;11(1):10. Available from: http://ijbnpa.biomedcentral.com/articles/10.1186/1479-5868-11-10

79. Payne K, Walton E, Burton C. Steps to benefit from social prescription: a qualitative interview study. Br J Gen Pract [Internet]. 2020 Jan;70(690):e36–44. Available from: http://bjgp.org/lookup/doi/10.3399/bjgp19X706865

80. Tava’E N, Nosa V. The green prescription programme and the experiences of Pacific women in Auckland. J Prim Health Care. 2012;4(4):313–9.

81. Stickley T, Hui A. Social prescribing through arts on prescription in a UK city: Participants’ perspectives (Part 1). Public Health [Internet]. 2012 Jul;126(7):580–6. Available from: http://dx.doi.org/10.1016/j.puhe.2012.04.001

82. Polley M, Bertotti M, Kimberlee R, Pilkington K, Refsum C, Carpenter A. A review of the evidence assessing impact of social prescribing on healthcare demand and cost implications [Internet]. London: University of Westminster; 2017 [cited 2018 Jun 10]. Available from: file:///Users/sarakalde/Library/Application Support/Mendeley Desktop/Downloaded/Polley et al. - 2017 - A review of the evidence assessing impact of social prescribing on healthcare demand and cost implications.pdf

83. Murphy SM, Edwards RT, Williams N, Raisanen L, Moore G, Linck P, et al. An evaluation of the effectiveness and cost effectiveness of the National Exercise Referral Scheme in Wales, UK: a randomised controlled trial of a public health policy initiative. J Epidemiol Community Health [Internet]. 2012 Aug;66(8):745–53. Available from: http://jech.bmj.com/lookup/doi/10.1136/jech-2011-200689

84. Dayson C, Bashir N, Bennett E, Sanderson E. The Rotherham Social Prescribing Service for People with Long-Term Health Conditions: Annual Evaluation Report. Sheffield: Sheffield Hallam University. Centre for Regional Economic and Social Research; 2016.

85. Brandling J, House W. Investigation into the feasibility of a social prescribing service in primary care: a pilot project [Internet]. Bath: University of Bath; 2007 [cited 2018 Jun 2]. Available from: http://opus.bath.ac.uk/22487/1/Brandling_SocialPrescribingFeasabilityReport.pdf

86. Mistry B, Phillips L, Simpson J. Prescription Plus Crawley. The Case for Project Expansion. Crawley; 2017.

87. Grayer J, Cape J, Orpwood L, Leibowitz J, Buszewicz M. Facilitating access to voluntary and community services for patients with psychosocial problems: a before-after evaluation. BMC Fam Pract [Internet]. 2008 Dec 7;9(1):27. Available from: http://bmcfampract.biomedcentral.com/articles/10.1186/1471-2296-9-27

88. Pescheny J, Randhawa G, Pappas Y. Patient uptake and adherence to social prescribing: a qualitative study. BJGP Open [Internet]. 2018 Oct 7;2(3):bjgpopen18X101598. Available from: https://bjgpopen.org/content/bjgpoa/2/3/bjgpopen18X101598.full.pdf

89. Pescheny JV, Pappas Y, Randhawa G. Facilitators and barriers of implementing and delivering social prescribing services: a systematic review. BMC Health Serv Res [Internet]. 2018 [cited 2018 Jun 12];18(1):86. Available from: https://www.ncbi.nlm.nih.gov/pmc/articles/PMC5803993/pdf/12913_2018_Article_2893.pdf
