# Supplementary material for: What does the literature mean by social prescribing? A critical review using discourse analysis
Source: Sociol Health Illn. 2022 Apr 11;44(4-5):848–68. doi: 10.1111/1467-9566.13468 (PMC9321825; doi:10.1111/1467-9566.13468)
Supplement: Supplementary file 4 — Supporting Information 4 [file SHIL-44-848-s002.docx]

**Appendix 4. Characteristics of studies included for analysis**

| **Authors (year)** | **Country** | **Type of paper** | **Study Design** | **Sample/Setting** | **Intervention** | **Objective** | **Outcome measures** |
| --- | --- | --- | --- | --- | --- | --- | --- |
| Rempel ES, et al. (2017) (1) | UK | Research (published paper) | Literature Review | 41 studies | ﻿A referral programme linking patients in health services with community-based activities | To identify the aims of social referral initiatives and identify the measures used to evaluate | - Cost savings - Resource reallocation - Mental, physical and social well-being (Warwick-Edinburgh Mental Well-being Scale - WEMWBS) |
| Woodall J, et al. (2018) (2) | UK | Research (published paper) | Mixed Methods Study | Patients (N ﻿342) referred to a SP programme operating in an area within a large city | Referral to a ‘Well-being Coordinator’ (link worker) who offered support and advice on local community-based groups and services (e.g. counselling, physical fitness classes, ﻿finance/debt advice) | ﻿To understand the outcomes of a SP service and the processes which supported SP delivery | - Quant: Mental wellbeing (WEMWBS), EQ-5D (which covers mobility, self-care, usual activities, pain/discomfort and anxiety/depression), social networks (Campaign to End Loneliness Measurement Tool), use of GP services - Qual: SP staff and users’ perspectives on the service |
| Darnton P, et al. (2018) (3) | UK | Research (web-based report) | Mixed Methods and Economic Evaluation | ﻿Patients (N 595) referred to a SP service provided by a voluntary sector partnership across five localities | Referral to a ‘Making Connection Coordinator’ (link worker) who undertook a guided conversation and provided advice on community-based resources over 3 months (e.g. finance/debt advice, ﻿befriending services, physical activity) (*Making Connection SP*) | To evaluate health- and cost-related outcomes of a SP service | - Quant: Questionnaires measuring health status, health confidence, personal wellbeing, experience. - Qual: strength of relationships, practical assistance, match between client and service, service infrastructure |
| Pescheny J, et al. (2018) (4) | UK | Research (published paper) | Qualitative Study | ﻿Patients (N 10), GPs (N 3) and navigators (N 2) involved in a SP programme delivered across 4 general practices | ﻿Referral to a navigator (link worker), who contacted primary care patients to arrange an initial appointment held in surgeries. Navigators could refer service users onwards to a maximum of 12 free community sessions. | To explore stakeholders’ views on factors influencing uptake and adherence to SP | - Factors affecting uptake and adherence: patients’ trust in GPs and navigators, service accessibility, service support, patients’ expectations, etc. |
| Bickerdike L, et al. (2017) (5) | UK | Research (published paper) | Literature review | ﻿15 studies | ﻿Programmes linking patient in primary care setting with a link worker or facilitator of SP | To assess evidence for SP effectiveness | - Uptake and attendance - Health and well-being (WEMWBS, HADS, GAD-7, PHQ-9, CORE-OM, WSAS, GHQ-12, COOP/ WONCA) - Healthcare usage outcomes - Patients’, referrers’ experience - Costs |
| Loftus AM, et al. (2017) (6) | UK | Research (published paper) | Before-and-after study | Patients (N 68) over 65 with a chronic condition who attended their GP frequently or had multiple medications, referred to a SP programme delivered in an urban practice | Referral to a SP coordinator (link worker) for assessment and selection of a 12-week community-based programme (e.g social clubs, counselling, exercises classes, etc.) | To evaluate the impact of SP on general practice workload and polypharmacy | - Use of GP Health Care (participants' contacts with GPs) - Number of repeat prescriptions per patient |
| Kilgarriff-Foster A, et al. (2015) (7) | UK | Research (published paper) | Literature review | 24 studies | ﻿Short-term intermediary services facilitating the engagement of patients with psychosocial needs with community-based non-clinical support services | ﻿To explore the key components and potential impact of SP programmes | - Health and well-being (WEMWBS, HAS, GHQ) - Health service use - Cost-effectiveness - Feasibility, acceptability |
| Chatterjee HJ, et al. (2018) (8) | UK | Research (published paper) | Literature review | 86 studies | Schemes linking patients in primary care with community non-clinical interventions (including arts, education, exercise) | To evaluate the effectiveness of UK SP schemes | - Mental well-being (GAD-7, GHQ, PHQ-9, WEBWMS, SWEBWMS, HADS) - Health and well-being (CO-OP/WONCA, SF-36, DTFS) - Social support - Cost effectiveness - Health service use |
| Chesterman D, et al. (2018) (9), linked to Mistry B, et al. | UK | Research (published paper) | Qualitative Action Research Study | Users and practitioners involved in commissioning or providing a SP pilot available in 4 urban general practices | Assessment by a ﻿Community Support Coordinator (link worker) and referral to community-based services (e.g. ﻿physical activities, counselling, advice around debt, housing, etc.) (Prescription Plus) | To evaluate and strengthen a local SP initiative, taking a ‘learning through doing’ approach | - Participants’ concerns and experiences - Co-operation between stakeholders - Service sustainability |
| Dayson C (2017) (10) | UK | Research (published paper) | Mixed Methods Case Study | Users (N ﻿108), commissioners (N 7) and providers (N 20) involved in a SP pilot covering a local authority area | Referral to an ‘Advisor’ (link worker) for assessment of patients’ support needs and onwards referral to pump-primed or wider voluntary and statutory services | To shed light on the ﻿epistemological and methodological challenges of social innovations, such as SP | - Quant: use of hospital resources (inpatient admissions, A&E Attendance, Outpatient appointments) - Qual: patient well-being and independence, sustainability of the VCS, collaboration |
| Ferguson K, et al. (2018) (11) | UK | Research (web-based report) | Mixed Methods Evaluation Study | Stakeholders involved in a borough-wide SP service covering 37 general practices | Initial assessment by a Social Prescriber (link worker) (﻿telephone or in face-to-face) and onwards referral to community-based sources of support (e.g exercise, weight management, learning and employment) | To assess the impact of a SP roll-out in service users, general practices and community organisations | - Quant: health and wellbeing (MYCaW), service-level data (number of referrals, etc.), users’ profile, costs data - Qual: service awareness, coordination, users’ wellbeing, service sustainability |
| Blickem C, et al. (2013) (12) | UK | Research (published paper) | Qualitative interview and focus group study | People with long term conditions attending health-related support groups and community centres in a local authority area | ﻿A community referral tool (PLANS) ﻿to tailor community-based resources to referred patients with long term conditions | ﻿To assist the development of a SP intervention designed to promote engagement and improve access to health-relevant resources | - Experiences and priorities of people with long term conditions (isolation, safety, linking to support, potential roles of the VCS, access to health education, practical support, lifestyle concerns, etc.) |
| Elston J, et al. (2019) (13) | UK | Research (published paper) | Before-and-after study  ﻿ | ﻿Older patients (>50) (N ﻿86) with multiple long-term conditions referred to a SP programme operating across 3 localities | Referral to a Well-being Co-ordinator (‘holistic’ link-worker) for a 12-week support intervention, including resilience-focused coaching and practical support to navigate and access local services. | To evaluate the impact of link-workers on service users’ well-being, activation and frailty, as well as service utilisation and associated costs. | - Health and wellbeing (Well-being Star, WEMWBS, RCFS) - Self-management (Patient Activation Measure) - Health and social care service use - Costs data |
| Whitelaw S, et al. (2017) (14) | UK | Research (published paper) | Qualitative Case Study | The steering group (N ﻿12), wider primary care team (N 10) and members of various community groups (N 8) involved in a SP scheme delivered across 2 general practices | ﻿Referral to a link worker for patients’ assessment and onwards referral to available community resources | ﻿To conduct a process-based evaluation of the inception and early implementation of a SP initiative | - Primary care perspectives: information governance, coordination between stakeholders, resource sufficiency, etc. - VCS perspectives: service quality, accessibility, training needs, etc. |
| White JM, et al. (2017) (15) | UK | Research (published paper) | Qualitative Interview Study | ﻿18 health professionals and 15 representatives of third sector organisations in socio-economically diverse, and ﻿mix of rural and urban locations | SP schemes linking patients in primary care with different community-based services (e.g. a ﻿‘lifestyle referral’ scheme, support for carers, etc.) | ﻿To analyse the quality of the relationships between primary healthcare professionals and VCS practitioners drawing on social capital theory | - Prescribers’ views: service awareness, professional roles, accountability - Providers’ views: barriers and facilitators to collaborative working |
| Polley M, et al (2017) (16) | UK | Research (web-based report) | Literature review | 14 studies | ﻿Referral pathways connecting patients in primary care with a ‘link worker’ and relevant non-medical services in the third sector | ﻿To appraise the current evidence as to whether SP reduces the demand for health services and is cost effective | - Health service use - Social and economic impact of SP (value for money assessments, SROI calculations) |
| Dayson C, et al. (2018) (17) | UK | Research (web-based report) | Before-and-after study | ﻿Patients referred by their GP to a SP service delivered in an urban area | Referral to a ‘community connector’ (link worker) for assessment of patients’ needs and interests, and identification of relevant local services and activities (*Community Connectors SP*) | ﻿To evaluate the early stages of a SP service measuring its impact on patients’ health and demand for primary and secondary care services | - Health and mental wellbeing (EQ-VAS, EQ-5D, SWEMWBS) - Social connectedness - Self-care - Primary and secondary care service use |
| Husk K, et al. (2019) (18) | UK | Research (published paper) | Realist Review | ﻿109 studies in the first phase, 34 studies in the second phase | ﻿Any referral pathway linking patients in primary care with activities undertaken in the community | ﻿To explore whether different methods of SP referral and supported uptake do (or do not) ‘work’ | - Barriers and facilitators to Enrolment, Engagement and Adherence |
| Pescheny JV, et al. (2019) (19) | UK | Research (published paper) | Before-and-after study | ﻿Patients in primary care referred to a SP programme (12 sessions) provided by third sector organisations | ﻿Referral to a link worker for initial assessment, identification of non-medical needs, motivational interviewing, continuous personalised support and referral to community-based activities (12 sessions, free of charge) | ﻿To assess the change in energy expenditure levels of service users after participating in a SP programme | - Changes in energy expenditure levels using the International Physical Activity Questionnaire (IPAQ) |
| Pescheny JV, et al. (2019) (20) | UK | Research (published paper) | Systematic Review | 16 studies | ﻿Services in primary care linking patients with non-medical needs to sources of support provided by the community and voluntary sector | ﻿To assess the evidence base for SP programmes based on primary care and involving navigators | - Health and wellbeing (WEMWBS, SWEMWBS, HADS, PHQ9, GAD7, MYMOP, GHQ-12, COOP/ WONCA) - Health-related behaviours - Self-concepts - Social contacts (Friendship Scale score, Duke-UNC FSSS) - Day-to-day functioning |
| Carnes D, et al. (2017) (21) | UK | Research (published paper) | Mixed Methods Study | ﻿Socially isolated patients referred to a SP programme delivered across 22 primary care general practices in a ﻿mixed socio-economic, multi-ethnic area | ﻿Referral to a SP coordinator (‘link worker’) for assessment, mutual agreement of a well-being plan over a maximum of 6 sessions and linkage into community organisations and services. | ﻿To investigate whether a SP service could be implemented in a general practice setting and evaluate its effect on patients’ well-being and primary care service utilisation | - Quant: mental wellbeing, primary health care resource use - Qual: patients’ satisfaction with the service, appropriateness and timing of referrals, barriers and facilitators to engagement, relationship between link workers and patients. |
| Thomson LJ, et al. (2015) (22) | UK | Research (web-based report) | Literature review | 35 studies | ﻿Any referral mechanism linking patients with non-medical sources of support within the community | ﻿To provide definitions, models and notable examples of SP schemes and to assess whether and how they have been evaluated | - Self-esteem, confidence, motivation - Mental wellbeing - Lifestyle - Primary care service use - Sociability, communication skills |
| Moffatt S, et al. (2017) (23) | UK | Research (published paper) | Qualitative Interview Study | Adults with long-term conditions referred to a SP prescribing programme delivered in a socio-economically deprived area across ﻿17 general practices. | ﻿Link Worker SP programme comprising personalised support to identify meaningful health and wellness goals, ongoing support to achieve agreed objectives and linkage into appropriate community services ﻿(e.g. welfare rights advice, walking groups, physical activity classes, arts) (*Ways to Wellness*) | ﻿To describe the experiences of patients with long-term conditions referred to a SP programme and identify its impact on patients’ health and well-being | - Self-confidence, self-reliance, resilience, personal responsibility - Health related behaviours (weight management, exercise, diet) - Mental wellbeing - Long term condition management |
| Wigfield A, et al. (2015) (24) | UK | Research  (web-based report) | Mixed Methods Study | Referrers from primary care and older patients (N 247) referred to a SP programme delivered across 3 localities | A SP scheme linking older patients in primary care with community activities delivered by third sector organisations (*Fit For the Future SP*) | ﻿To evaluate the potential impact of a SP initiative in the outcomes of older people | - Quant: mental wellbeing (WEMWBS), lifestyle (physical activity, diet, alcohol, cigarette), clinical data (BMI, waist circumference), social networks, satisfaction with life, self-management. - Qual: health care professionals’ expectations of SP, suggestions for improvement, lessons learned. |
| Hamilton-West K, et al. (2019) (25) | UK | Research  (published paper) | Evaluability Assessment Study | Stakeholders involved in two SP programmes. One of them delivered from primary care and the other from secondary care settings. | Two SP services linking patients from health services with community-based resources (﻿*Community Wellbeing Service, ﻿Encompass SP Service*) | ﻿To inform the design and evaluation of SP schemes through an Evaluability Assessment approach. | - Recommendations to allow for future service evaluation (e.g., related to data monitoring systems, information governance, etc.) |
| Wildman JM, et al. (2019) (26) | UK | Research  (published paper) | Qualitative Follow-up Study | Users (N 24) of a link-worker SP service delivered in a socio-economically deprived area across ﻿17 general practices | Link Worker SP programme comprising personalised support to identify meaningful health and wellness goals, ongoing support to achieve agreed objectives and linkage into appropriate community services ﻿(e.g. welfare rights advice, walking groups, physical activity classes, arts) (*Ways to Wellness*) | ﻿To explore experiences of SP among people with long-term conditions one to two years after their initial engagement with the service. | - Service users’ relationships with link workers - Factors involved in making progress in behaviour change and long term condition management - Setbacks and barriers to maintaining change - Fluctuating levels of engagement |
| Hanlon P, et al. (2019) (27) | UK | Research (published paper) | Qualitative Interview Study | ﻿Patients (N 12) referred to a Links Worker SP Programme delivered across ﻿general practices in areas of high socioeconomic deprivation | Referral to a community link worker for one-to-one assessment, support and linkage into relevant community resources (﻿*‘Deep-End’ Links Worker Programme*) | ﻿To explore the utility of Self-Determination Theory in understanding the impact of a ﻿Links Worker SP Programme in patients’ wellbeing | - Overall perceived improvement in daily life - Barriers and facilitators to improvement (related to relatedness, competence, autonomy, beneﬁcence and regulation of behaviour) |
| Wildman JM, et al. (2019) (28) | UK | Research  (published paper) | Qualitative Interview and Focus Groups Study | ﻿Link workers (N 41) involved a social prescribing scheme operating in a socioeconomically deprived area across ﻿17 general practices | Link Worker SP programme comprising personalised support to identify meaningful health and wellness goals, ongoing support to achieve agreed objectives and linkage into appropriate community services ﻿(e.g. welfare rights advice, walking groups, physical activity classes, arts) (*Ways to Wellness*) | ﻿To explore link workers’ own definitions of their role in SP and the skills and qualities identified by themselves as necessary for effective patient linkage | - Realities and complexities of the link worker role - Barriers to performing the role (referral challenges, onward referral challenges, boundary setting). |
| Swift M (2017) (29) | UK | Research (published paper) | Mixed Methods Study | Stakeholders involved in a ﻿community-centred model of health delivered across 17 GP practices ﻿in two neighbouring towns with great health inequalities | ﻿A co-created ﻿community-centred model of health, including a pathway to link patients with community-based activities and services (Community Wellbeing Practices model SP) | To describe and reflect on the co-design and implementation of a ﻿community-centred model of health (including SP) | - Quant: mental wellbeing (SWEMWBS, PHQ9, GAD7), health status (EQ5D) - Qual: health-related goals, satisfaction levels, social connectedness, etc. |
| Wessex Academic Health Science Network (2017) (30) | UK | Research (web-based report) | Mixed Methods Study | Stakeholders involved in 8 different SP programmes covering a population of ﻿1 million people | 8 SP schemes linking patients in primary care with community-based resources and services | ﻿To evaluate the impact of a SP programme in patients’ wellbeing and service utilisation, and support its development and spread | - Quant: patient reported outcomes (health status, health confidence, wellbeing, experience of service), staff reported outcomes (work wellbeing, job satisfaction), health service use, costs. - Qual: social connectedness, continuity of care, satisfaction levels, etc. |
| Beech R, et al. (2017) (31) | UK | Research  (published paper) | Realist Evaluation (mixed methods design) | SP recipients, carers, link workers and service providers involved in a SP initiative delivered in ﻿one large city GP practice and a specialist centre based in a community hospital | ﻿Assessment by a link worker at the GP surgery or hospital. Onward referral to community services and provision with relevant information and support (*The Wellbeing Coordinator service*) | To assess the outcomes of the SP service in terms of benefits experienced by recipients and carers and to understand how care processes are perceived by link workers and other service providers | - Quant: mental wellbeing (SWEMBS, ONS) - Qual: users’, carers’, service providers’ and health workers’ perspectives on the service (satisfaction, perceived strengths and limitations, expectations, etc.) |
| Coan J (2016) (32) | UK | Research (web-based report) | Mixed Methods Research | Users, link workers, primary care workers and community providers involved in a SP programme delivered across 6 general practices | Referral to a SP coordinator (link worker) for patient assessment and provision of onwards supported referral to relevant community-based services (e.g. lifestyle support, social groups, social welfare) | To describe a SP programme and explore stakeholders’ perception on its potential impacts | - Quant: satisfaction level (end-of-service questionnaire to patients and healthcare workers’), mental wellbeing (SWEMWBS) - Qual: feedback from patients and community service providers (satisfaction, perceived strengths and limitations, expectations, etc.) |
| Bertotti M, et al. (2015) (33) | UK | Research  (web-based report) | Mixed Methods Study (including an Economic Evaluation) | ﻿Stakeholders of a SP programme delivered across 23 general practices and 85 statutory and voluntary groups in a socio-economically diverse urban area | Referral to a trained SP coordinator (link worker) for assessment and supported, personalised linkage into community-based resources | To evaluate the impact of a SP programme in patients’ wellbeing, health service utilisation and associated costs | - Quant: general health and wellbeing, mental wellbeing (anxiety and depression), active engagement in life, A&E visits, costs. - Qual: users’ experience with the service (satisfaction, expectations, etc.) |
| Skivington K, et al. (2018) (34) | UK | Research (published paper) | Qualitative Interview Study | ﻿VCS representatives (N 30) and link workers (N 6) involved in a SP programme delivered in socio-economically deprived urban areas | ﻿﻿Referral to a trained community link worker to support patients in accessing community organisations (*Links Worker Programme*) | ﻿To investigate potential factors associated with successful implementation of a SP programme | - The role and capacity of link workers - Contextual factors that affect SP implementation - Benefits and challenges to collaborative working |
| Southby K, et al. (2018) (35) | UK | Research (published paper) | Qualitative Case Study | ﻿Primary care workers and representatives of voluntary and community sector organisations engaged in collaborative programmes in socio-economically deprived urban areas | 4 SP schemes linking patients in primary care with activities provided by the voluntary and community sector (e.g. ﻿weight management, diet, exercise, employability) | ﻿To add to the knowledge base around collaborative practice between general practices and voluntary and community sector organisations by examining the factors that aid or inhibit such collaboration | - Modes and outcomes of GP- VCS collaboration - Facilitators to GP-VCS collaboration (equitable relationships, communication stability, etc.) - Barriers to GP-VCS collaboration (policy contingencies, misconceptions, etc) |
| Heijnders ML, et al. (2018) (36) | The Netherlands | Research (published paper) | Qualitative Interview Study | ﻿Patients (N 10) with psychosocial problems referred from 4 primary care centres to a social well-being organisation | ﻿﻿Referral to social well-being organisation, followed by an ‘holistic’, personalised assessment by a well-being coach based on social activation theory. Onwards linkage into community-based activities (e.g. cooking classes) | ﻿To determine what happens in the chain of the SP and what changes the participant experiences in terms of social participation. | - Participants’ life events - Insights on the referral and intake process - Personal strength and responsibility - Self-reliance - Social activation/participation |
| Centre for Reviews and Dissemination. ﻿University of York (2015) (37) | UK | Research  (web-based report) | Rapid Review | 22 studies | ﻿SP schemes linking patients in primary care with sources of support within the community | To review the effectiveness and cost effectiveness of SP programme | - Mental and physical wellbeing (WWQ, PHQ9, GAD7, IPAQ, HAD, COOP chart, WEMWBS, WSAS, Dynamic Observation scale) - SROI analysis - Cost-effectiveness |
| Pescheny JV, et al. (2018) (38) | UK | Research (published paper) | Literature Review | 8 studies | ﻿Referral schemes that link patients in primary care with local services and activities provided by the third sector (community, voluntary, and social enterprise sector) | ﻿To identify factors that facilitate and hinder the implementation and delivery of SP services based in general practice and involving navigators | - Facilitating factors: implementation approach, organisation and management, attitudes, support and supervision, communication, organisational readiness, staff engagement, infrastructure - Barriers: leadership, implementation approach, economic climate and funding, shared understanding, staff engagement and turnover, patient engagement, infrastructure, quality appraisal |
| South et al. (2008) (39) | UK | Research (published paper) | Qualitative Case Study | Primary care workers (N 8) and patients (N 10) referred to SP delivered across 2 general practices located in disadvantaged urban areas | ﻿Up to 3 appointments with a link worker to discuss needs and identify appropriate sources of local support (e.g. ﻿volunteering, debt advice, luncheon clubs, etc.) | ﻿To explore the concept of SP and discuss its value as a public health initiative embedded within general practice | - The potential of SP on   (1) Extending primary care,  (2) Addressing public health issues,  (3) Building health alliances |
| Bertotti M, et al. (2018) (40) | UK | Research (published paper) | Realist Evaluation (Mixed Methods Study) | ﻿Users, commissioners, and primary care workers involved in a SP scheme delivered across 23 practices in a socio-economically diverse urban area | Referral to a trained SP coordinator (link worker) for assessment and supported, personalised linkage into community-based resources | ﻿To evaluate a SP pilot, by unpacking the contextual factors and mechanisms that might influence its implementation and development | - Relevant contextual factors and mechanisms related to   (1) GP referral process,  (2) Consultation with the link worker,  (3) Interaction with VCS organisations |
| Bragg R, et al. (2017) (41) | UK | Research  (web-based report) | Evidence Synthesis | 12 England-based SP services | SP services that are well established, have a good track record in terms of numbers of patients involved, have been evaluated, are operating at scale (e.g. with at least one CCG) and show the diversity of SP models. | To develop an understanding of the use of nature-based-interventions within social prescribing services and provide suggestions for good practice | - Characteristics of SP services (including referral and funding mechanisms) - Characteristics of service users - Evidence of effectiveness and cost effectiveness (general wellbeing, health service use, SROI) |
| Bungay H, et al (2010) (42) | UK | Research (published paper) | Evidence Synthesis | Grey literature on UK-based Arts on Prescription (AoP) | SP schemes linking patients in health services with community-based art initiatives facilitated by artists (*AoP*) | To review current AoP initiatives in the UK and reflect on the challenges of providing evidence for their effectiveness | - Policy context for AoP - Evidence on effectiveness: mental well-being (WEMWBS, HADS), quality of life, social inclusion - Existing challenges for AoP |
| Grant C, et al. (2000) (43) | UK | Research (published paper) | Randomised Controlled Trial | 161 patients identified by their GP as having psychosocial problems in 26 general practices with varied socioeconomic characteristics | Referral to a liaison organisation for assessment of patients’ needs and linkage into voluntary organisations | To compare wellbeing and resource utilisation among patients referred to a SP scheme, with patients receiving routine general practitioner care. | - Primary outcomes: psychological wellbeing (HAD scale), social support (Duke-UNC FSS questionnaire) - Secondary outcomes: quality of life (COOP/WONCA, delighted-terrible faces scale), costs (service use, prescribing, referrals) |
| Jensen A, et al. (2019) (44) | Denmark | Research (published paper) | Qualitative Study | Patients (N 7) with mild-moderate mental health problems referred to an AoP programme delivered across a local authority area | Referral from a health service to a 10-week project offering a variety of arts and cultural activities averaging 2 hour sessions 2.5 times a week (Culture Vitamins – AoP) | To evaluate and explore the impact of an AoP programme in participants’ mental health wellbeing | - Mental wellbeing (sense of coherence, sense of meaning) - Capacity to overcoming challenges (motivation, self-esteem, resilience, ability to socialize) - Moving from self-critical to self-caring |
| Jensen A, et al. (2017) (45) | Sweden, Norway, Denmark, UK | Research | Rapid Review | 34 studies | Referral from a health service to community-based arts activities (Arts on Prescription – AoP) | To provide an overview of how AoP is delivered in Scandinavian countries and the UK | - Institutional context: political support and recognition, existence of specific research centres - Evidence for AoP effectiveness: quality of life, work ability, self-confidence, motivation, social and communication skills |
| Maughan DL, et al (2016) (46) | UK | Research (published paper) | Observational Study | Patients from an urban primary care centre, diagnosed with common mental health conditions and referred to a SP service | Referral to a ‘link worker service’ aimed at connecting people with community organisations. Patients were discharged when they were engaged in the community (maximum of 20 appointments) (Connect Project) | To assess the effects of a SP service on healthcare use and the subsequent economic and environmental cost | - Number of GP appointments - Prescriptions of psychotropic medications - Number of secondary-care referrals |
| Redmond M, et al. (2018) (47) | UK | Research | Qualitative Follow-up Study | Individuals (N 1297) referred to an art-related SP programme from primary care settings | ﻿Referral to an 8- or (formerly) 10-week course of creative activities, led by a local artist. | ﻿To explore the ﻿impact of an arts referral programme in service users’ well-being | - Social connectedness - Physical and mental well-being - Self-management |
| Age Concern Support Services (2011) (48) | UK | Research (web-based report) | Mixed methods (Qualitative case studies, before-and after survey) | Older people who had mild-moderate depression or were socially isolated referred to a SP programme delivered across 12 GP practices | In-depth assessment of the older person’s social, emotional and practical support needs, and onwards referral to Age UK services (including befriending, social groups, benefit checks and Fit as a Fiddle classes) | To assess the effectiveness of social prescribing for older people with mild to moderate depression or who are lonely and socially isolated | - Quant: mental well-being (WEMWBS) - Qual: users’ experience with the service (satisfaction, expectations, etc.) |
| Stickley T, et al. (2013) (49) | UK | Research | Qualitative follow-up study | 10 SP users | Referral to a 8- or (formerly) 10-week course of creative activities, which are led by a local artist in community locations. There are usually no more than 10 participants per group. (AoP) | To explore the long-term effects of an ‘Arts on Prescription’ SP service | - Self-conﬁdence, feelings of self-worth, self-perception - Social and communication skills - Motivational and aspirational changes |
| Stickley T, et al. (2012a)(50) | UK | Research | Qualitative Interview Study | ﻿10 referrers from ﻿primary care, secondary mental health care and the voluntary sector who had referred more than 1 person to SP | Referral to a 10-week blocks of art sessions led by professional artists in community locations. Usually with no more than 10 participants per group (AoP) | ﻿To investigate the views of referrers to AoP regarding the quality and effectiveness of the service | - Perceived impact of AoP on service users (personal benefits, social benefits) - Contextual views (policy environment, practical concerns) |
| Stickley T, et al. (2012b)(51) | UK | Research | Qualitative Interview Study | ﻿Patients with mental health issues referred to a SP service led by professional artists in community locations | ﻿Referral to a the 10-week blocks of art sessions led by professional artists in community locations. There are usually no more than 10 participants per group (AoP) | ﻿To explore the experiences of people who have engaged with AoP programmes | - Social: sense of social belonging, peer support, etc. - Psychological: self-awareness, self-discovery, etc. - Occupational: meaningful occupation and vocation |
| Sumner RC, et al. (2019) (52) | UK | Research | Observational Study | Patients (N 1297) referred to an Arts on Prescription SP programme by primary care professionals | Referral to an 8- or (formerly) 10-week course of creative activities (ranging from painting, to ceramics, playwriting, and mosaics), led by a local artist. ﻿ | ﻿To identify potential factors associated with attendance, engagement and wellbeing change of patients referred to AoP. | - Wellbeing (WEMWBS) - Programme attendance and engagement |
| Thomson L, et al. (2018) (53) | UK | Research (published paper) | Mixed-Methods Study | Participants (N 115) aged 65–94 at risk of loneliness and social isolation referred to museum-based programmes by health and social care, museum facilitators and volunteers. | A museum-based intervention consisting of 10-week programmes of engaging, creative and socially interactive sessions, of around 2 hours each, comprising curator talks, behind-the-scenes tours, object handling and discussion, and arts activities inspired by the exhibits. | To assess psychological wellbeing in a novel SP intervention for older adults called Museums on Prescription and to explore the extent of change over time | - Quant: Psychological wellbeing (Museum wellbeing Measure for Older Adults - MwM-OA) - Qual: perceptions and experiences of the participants and their carers where present, museum facilitators and volunteers. |
| Creative Alternatives (2009) (54) | UK | Research (web-based report) | Before-and-after Study | Patients (N 187) with mild to moderate depression, stress or anxiety referred to an AoP programme delivered in an urban area | A free programme of creative activities over six months, comprising weekly core workshops on expressive work in the visual arts, creative writing, storytelling, photography, pottery, etc. (*Creative Alternatives - AoP*) | To assess the effectiveness of an AoP scheme in participants’ health and wellbeing | - Mental wellbeing (HAD Scale, The Dartmouth COOP Chart) - Lifestyle modification (The Creative Alternatives Lifestyle Questionnaire) |
| Dayson C et al. (2013) (55) | UK | Research (web-based report) | Mixed Methods Study | Patients with long term conditions and their carers (N 808) referred to a SP programme delivered in an urban area across 28 GP practices | Referral to a link worker (advisor) for assessment of support needs before referring to funded VCS services (e.g advice and information, leisure-social activities, exercise, etc.) (*Rotherham* *SP Pilot*) | To evaluate the effectiveness and cost-effectiveness of a SP pilot project aimed at patients with long term conditions and their carers | - Quant: number of referrals into SP and the VCS, hospital episodes data, social outcomes - Qual: perceptions and experiences of public sector stakeholders, the VCS and project staff |
| Faulkner M (2004) (56) | UK | Research (published paper) | Qualitative Pilot Study | Patients with psychosocial issues (N 11) and volunteer advisors (N 8) involved in a SP scheme delivered in an urban busy general practice | ﻿Appointments with 2 volunteer link workers responsible for leading discussions and arranging appointments ﻿with community-based services (﻿over 150 organisations) | To describe and analyse the key features of a SP scheme, its perceived effectiveness, and any barriers to effective service provision. | - Key features of the SP scheme - Perceived effectiveness (social connectedness, distress levels, primary care service use) - Barriers to effective service provision |
| Roessler K (2011) (57) | Denmark | Research (published paper) | Qualitative Follow-up Study | ﻿Patients referred from a primary care centre to a community-based exercise programme delivered in an urban area | ﻿4 months of twice weekly supervised community-based physical training in groups (*Exercise on Prescription - EoP*) | To examine psychological aspects of intra- and interpersonal learning for patients with diabetes, hypertension, dyslipidaemia referred to EoP | - Motives and barriers in exercise participation - Characteristics of the ‘non-completers’ - Characteristics of the ‘completers’ |
| Baines A (2015) (58) | UK | Research (web-based report) | Mixed-Methods Evaluation Study | ﻿Primary care referrers, link workers and patients referred to a SP programme delivered across 4 mixed urban and rural practices | Referral to volunteer link workers (‘Navigators’, ‘Health Buddies’) for assessment adn supported referral to community-based services (e.g. dancing, volunteering, housing/homelessness, yoga, etc.) (﻿*ConnectWELL SP Programme*) | To evaluate the impact and cost-effectiveness of a SP programme and identify potential gaps and barriers | - Quant: mental well-being (WEMWBS), costs - Qual: SP users’, link workers’, primary care workers’, VCS representatives’ experience with the service (satisfaction, expectations, etc.) |
| Edmunds et al. (2007) (59) | UK | Research (published paper) | Before-and-after Study | ﻿Participants ranged in age from 16 to 73 years and diagnosed as overweight/obese, referred by their GP to an EoP programme delivered in an urban area | ﻿Referral to an advisor (i.e., a health and fitness instructor with specialized training to deliver exercise prescriptions) for a 3-month exercise routine suited to each patient’s condition (EoP) | ﻿To examine perceived autonomy support, psychological need satisfaction, self-determined motivation, exercise behaviour, exercise-related cognitions and general well-being amongst overweight/obese individuals referred to EoP | - Perceived autonomy support - Psychological need satisfaction - Motivational regulations for exercise (BREQ-2) - Exercise behaviour (GLTEQ) - Self-efﬁcacy - Commitment - Behavioural intention to exercise - Subjective vitality - Satisfaction with life |
| Jones et al. (2005) (60) | UK | Research (published paper) | Before-and-after Study | ﻿Participants (N 119) ﻿suffering high blood pressure, weight or stress related problems referred by a primary care worker to an EoP scheme delivered in 7 ﻿sports centres across different localities | 24 exercise sessions spread over 12 weeks. The exercise sessions consisted of gym-based, structured physical activity. Programme specification regarding equipment, intensity, duration was designed for each individual on the basis of an initial fitness assessment (*EoP*) | ﻿To investigate the role of participant expectations, self-efficacy, stage of change and psychological well-being in adherence to EoP, and assess the impact of failure to adhere on self-efficacy and psychological well-being | - Physical assessment (weight, BP) - Psychological measures (present level of activity, assessment of stage of change, exercise self-efﬁcacy, expectations and achievement of change, psychological well-being) |
| Murphy et al. (2012) (61) | UK | Research (published paper) | ﻿Pragmatic randomised controlled trial with nested economic evaluation | ﻿Inactive participants (N 1080) with coronary heart disease risk, mild to moderate depression, anxiety and/or stress referred by a primary care worker to an EoP scheme ﻿operating in 12 local health board areas | ﻿16-week tailored exercise programme supervised by a qualified exercise professional and ﻿delivered at leisure centres. The scheme included motivational interviewing, goal setting and relapse prevention (*National Exercise Referral Scheme - NERS*) | To assess ﻿the effectiveness and cost effectiveness of an EoP programme in increasing physical activity and reducing anxiety and depression | - Physical activity (7D-PAR, Baecke) - Health-related quality of life (EQ-5D) - Mental well-being (hospital anxiety and depression scale – HADS). - Costs |
| Rahman R, et al. (2011) (62) | UK | Research (published paper) | Before-and-after Study | ﻿Patients (N 293) identified by their GP as inactive and referred to an EoP programme operating in six council owned leisure centres | ﻿An induction followed by twice weekly exercise classes supervised by an exercise leader. ﻿Exercise schemes were individually tailored to suit referral conditions and delivered free of charge (*EoP*) | ﻿To examine psychological need satisfaction and motivational regulations as predictors of psychological and behavioural outcomes in EoP programmes | - Motivation (BREQ-2) - Mental wellbeing (HADS, Satisfaction with Life Scale) - Health-related quality of life (SF-36v2) - Physical activity level (Baecke’s Questionnaire of Habitual Physical Activity) - Adherence |
| Morton K, et al (2008) (63) | UK | Research (published paper) | Before-and-after Study | ﻿Patients (N 30) referred from primary care to an EoP scheme delivered in a local leisure centre | ﻿Twice weekly exercise sessions over 6 weeks (*EoP*) | ﻿To examine whether self-determined motivation is fostered through an exercise referral scheme, and the extent to which patient motives are related to their exercise adherence | - Motivation (BREQ-2, self-determination score) |
| Kimberlee RH (2013) (64) | UK | Research (web-based report) | Mixed Methods Study, including a Literature Review | ﻿Service users, practitioners and commissioners involved in SP programmes delivered across 7 general practices in an urban area | Different referral pathways to community resources (SP as signposting, SP light, SP medium, SP holistic) | ﻿To provide an outline of different SP models and assess their impact, effectiveness and cost-effectiveness | - Characterisation of existing SP models - Quant: SP effectiveness (Inventory for Brokerage Service Outcomes Star (IBSO)) - Qual: SP users’, link workers’, primary care workers’, VCS representatives’ experience with the service (satisfaction, expectations, etc.) |
| Public Health England (2019) (65) | UK | Research (web-based report) | Evidence Synthesis (rapid review) | 8 studies | ﻿Referral from healthcare professionals in primary care settings to a link worker or SP facilitator | ﻿To investigate the effectiveness of SP in the UK | - Contact with primary health care services - Changes in physical and/or mental health (WEMWBS, HADS, HRQL EQ-5D5L, ICECAP-A) |
| Duda J, et al. (2014) (66) | UK | Research (published paper) | ﻿Cluster randomised controlled trial | ﻿Individuals (N 347) referred from 13 primary care centres to an EoP scheme delivered across ﻿13 leisure centres in a large city | ﻿Exercise referral intervention grounded in Self Determination Theory over 3 months, including motivational interviewing, goal setting and ﻿a self- management exercise promotion booklet (*EoP*) | ﻿To test the feasibility and impact of a Self Determination Theory-based (SDT) exercise referral consultation | - Self-reported physical activity (7-day PAR) - Physical health outcomes (BMI, BP) - Health status (Dartmouth CO-OP Chart Scales) - Mental wellbeing (HADS, SVS) - Motivation-related processes of change measures (HCCQ, PNSES, BREQ-2) |
| Kok M, et al. (2016) (67) | UK | Research (web-based report) | Before-and-after Study | ﻿Patients (N 811) at risk of or recently diagnosed with type 2 diabetes referred from primary care to a community-based diabetes prevention programme delivered across 2 urban and rural localities | ﻿A 12-month programme that starts with a GP surgery invitation letter and eligibility check; followed by a 4-week group course with trained facilitators, follow-on one-to-one contacts, and referral to selected healthy lifestyle activities (﻿*Living Well, Taking Control*) | ﻿To evaluate the effectiveness and cost-effectiveness of a community-based type 2 diabetes prevention programme | - Biometric measures (weight, HbA1c, BMI, waist circumference, BP) - Questionnaire on quality of life (EQ-5D), overall life satisfaction (LSS), mental wellbeing (SWEMBS, CESD-7), physical activity (IPAQ), diet (FFQ), motivation to behavioural change |
| Mercer S, et al. (2017) (68) | UK | Research (web-based report) | ﻿Mixed-methods study | ﻿All practice staff (such as GPs, practice nurses, receptionists and health care assistants) of 7 GP practices involved in the SP scheme and adult patients (N ﻿288) referred to the Community Link Worker | The provision of ﻿a Community Links Practitioner and a ﻿practice development fund to support patients’ referrals to community services (﻿*‘Deep-End’ Links Worker Programme*) | ﻿To assess the implementation and impact of a Links Worker SP Programme at patient, practice and community levels | - Quant: quality of life (EQ-5D-5L), mental wellbeing (ICECAP-A, HADS), work-social functioning, life-style behaviours (alcohol, smoking, exercise), healthcare utilisation. Practice level: team climate, job satisfaction, morale, burnout - Qual: barriers and facilitators to program implementation |
| Mills H, et al. (2012) (69) | UK | Research (published paper) | Mixed Methods Study | ﻿Patients (N ﻿1,315), exercise providers and referring primary care health professionals involved in an EoP programme delivered in 5 urban local leisure centres | ﻿Referral to a patient-centred physical activity program over 26 weeks, including individual and group exercise sessions ﻿in gyms, exercise studios and swimming pools (*EoP*) | ﻿To explore and reveal the constituents of ‘‘success’’ in exercise referral schemes through comparison, contradiction, and integration of qualitative and quantitative research findings | - Qual: the perception of success according to participants, referrers, and exercise providers - Quant: programme completion, weight loss, mean arterial pressure reduction |
| Dayson C, et al. (2016) (70) | UK | Research (web-based report) | Mixed Methods Study and Economic Evaluation | Staff members (N 7), volunteers (N 2) and patients (﻿N 939) referred from primary care to a SP scheme delivered across an entire metropolitan borough | Referral to a link worker (advisor) for assessment of support needs before referring to appropriate VCS services (e.g advice and information, leisure-social activities, exercise, etc.) (*Rotherham* *SP*) | ﻿To provide an updated assessment of the social and economic impact of a SP programme | - Quant: Demand for urgent hospital care, well-being and positive functioning, economic and social cost-benefit - Qual: staff members’, volunteers’ and users’ experiences |
| Kimberlee RH (2016) (71) | UK | Research (web-based report) | Mixed Methods Study and Economic Evaluation | Referring GPs, link workers, members of community organisations and patients (N ﻿2047) referred to a SP programme operating across a county | Referral to a link worker for assessment of support needs before referring to VCS services (e.g. welfare services, exercise activities, arts classes) (*Gloucestershire SP*) | ﻿To evaluate the impact of an expanded SP scheme in patients’ well-being and service use | - Quant: mental wellbeing (WEMWBS), hospital and primary care attendance - Qual: stakeholders’ views and experiences |
| Rouse O, et al. (2011) (72) | UK | Research (published paper) | Cross-Sectional Study | ﻿Patients (N 347) referred from primary care to an exercise referral scheme in a large city | Referral to an EoP scheme (*EoP*) | To explore the ﻿role of autonomy support on the motivation, mental health and intentions of EoP participants | - Emotional well-being: subjective vitality (SVS) and depressive symptoms (HADS-D) - Physical activity intentions |
| Brandling J, et al. (2007) (73) | UK | Research  (web-based report) | Mixed Methods Pilot Study | Community stakeholders, primary care workers and patients defined as ‘high resource users’ linked to 3 GP surgeries | ﻿A SP service, to be designed based upon the finding of the study | ﻿To explore the feasibility of developing a SP service to reach a significant proportion of primary care high resource users | - Quant: health resource use - Qual: opinions on potential SP users, benefits and drawbacks of SP, barriers to accessing the service |
| Wormald H, et al (2006) (74) | UK | Research (published paper) | Qualitative Focus Group Study | 16 participants referred to an EoP programme delivered in ﻿the most socio-economically deprived areas of a city | Referral to an advisor for ongoing support in the form of up to 6 monthly progress consultations and optional ongoing referred to VCS activities (﻿*Active Lifestyles – EoP*) | ﻿To explore participants' perceptions of the operation and effectiveness of a community-based physical activity service | - Participants’ views on the referral process, operational aspects of the service, and perceived benefits |
| Kier Business Services Limited (2016) (75) | UK | Research  (web-based report) | Mixed Methods Evaluation | Referring primary care workers, patients, link workers and community stakeholders involved in a SP programme operating across ﻿37 practices | ﻿Referral to a link worker ﻿for assessment of patients’ needs and linkage into third sector, statutory services or community-based activities  (*Patient Empowerment Project*) | To evaluate effectiveness and cost-effectiveness of a SP programme focused on individuals with long-term conditions | - Quant: patients’ health and wellbeing (clinical data, SWEMWBS, EQ-5D-5L), self-efficacy and management, (ONS items), healthcare utilisation - Qual: stakeholders’ perceptions about the service |
| Mistry B, et al. (2017) (76) | UK | Research  (web-based report) | Mixed Methods Study | Patients (N 28) referred from primary care to a SP programme delivered across 4 urban general practices | Assessment by a ﻿Community Support Coordinator (link worker) and referral to community-based services (e.g. ﻿physical activities, counselling, advice around debt, housing, etc.) (*Prescription Plus*) | To assess the impact of a SP scheme in patients’ wellbeing and service utilisation | - Quant: patients’ wellbeing, health services use - Qual: stakeholders’ experiences and perceptions about the service |
| ﻿Jones T, et al. (2009) (77) | UK | Research  (web-based report) | Mixed Method Study | Project staff (N 28) and service users (N 40) involved in a SP scheme operating across an entire region | SP programmes offering physical exercise, healthy eating, mental well-being and general well-being activities (﻿*South West Well-being*) | To evaluate a SP ﻿programme in the first year of delivery and identify emerging themes across the programme. | - Quant: general health, physical activity, diet related behaviour, mental and social well-being - Qual: service staff and users’ experiences on the service |
| Tava'e N, et al. (2011) (78) | ﻿New Zealand | Research (published paper) | Qualitative Study | ﻿Pacific women (N 20) aged 40 years and older referred from primary care to an EoP programme and discharged as independently active | Referral to a supported, tailored exercise programme comprising education workshops covering topics such as nutrition, healthy lifestyle, etc. (EoP) | ﻿To investigate the experience of Pacific women referred to a Green Prescription programme | - Past history of physical activity - Views and experiences about the programme - Influences on programme adherence or dropout rates - Health perception |
| Kimberlee R, et al (2015) (79) | UK | Research (published paper) | Qualitative Study | ﻿SP practitioners, local council/Public Health employees, GPs and patients participating in SP programmes operating in different localities | Different referral pathways to community resources (SP as signposting, SP light, SP medium, SP holistic) | To explore the meaning and definition of SP, and describe different programme types | - Different SP models based on their community embeddedness |
| **﻿**ERS Research and Consultancy (2013) (80) | UK | Research  (web-based report) | Mixed Methods Study | ﻿Service users, healthcare practitioners, members of the steering group involved in a SP scheme operating in a socio-economically diverse city | ﻿Referral to one of the 5 collaborating Linkwork Organisations. Initial assessment and onward referral to community-based activities | To provide an assessment of the impact and achievements of a SP scheme, and document lessons learned to inform future practice. | - Quant: number for referrals, reason for referral, primary goals set, mental wellbeing (SWEMWB, mean confidence scores) - Qual: stakeholders’ views and experiences around SP |
| Grayer J, et al. (2008) (81) | UK | Research (published paper) | Before-and-after Study | Patients with psychosocial problems referred by primary care practitioners to a SP scheme being delivered across 13 urban general practices | Initial assessment by a graduate primary care mental health worker and onwards referral to community resources with different, personalised levels of support | To evaluate the acceptability and effectiveness of a SP programme facilitating access of patients with psychosocial problems in primary care to VCS services | - Mental wellbeing (GHQ-12, COREOM) - Social outcomes (WSAS) - Patient satisfaction (CSQ, community link evaluation) - Use of primary care resources |
| Aggar C, et al (2020) (82) | Australia | Research (published paper) | Before-and-after Study | 13 patients with mental health problems referred from primary care to a SP scheme delivered in an urban setting | Referral to a link worker for assessment and discussion on available community-based resources, and provision of weekly arts and crafts group sessions (2-3h for 10 weeks) | To evaluate whether a SP pilot program can improve participants’ quality of life, and social and economic participation. | - Quality of life (WHOQoL) - Welfare needs and support (CANSAS) - Health Status and self-efficacy (EQ5D) - Psychosocial distress (Scale K10) - Loneliness and social participation (UCLA) - Economic participation - Hospital admission |
| Beardmore A (2020) (83) | UK | Research (published paper) | Qualitative Study | 8 primary care and VCS workers involved in a SP scheme delivered in an urban and suburban area | ‘Medium’ and ‘Holistic’ SP schemes, as specified by Kimberlee (79) | To explore who works in SP and how they experience their role | - Narrative accounts of those working in SP |
| Tierney S, et al (2020) (84) | UK | Research (published paper) | Realist review | 118 studies | Primary care-based SP schemes focusing in adults (+18) | To understand how SP might work, for whom, in what circumstances and how to optimise delivery within primary care | - A refined programme theory on how connector roles, especially link workers, work in practice |
| Kellezi B, et al (2019) (85) | UK | Research (published paper) | Mixed methods | GPs, SP providers and patients experiencing loneliness referred to a SP scheme delivered in the English East Midlands. | Initial meeting and needs assessment by a health coach. Onwards prescription of self- care management or referral to a link worker for linkage into VCS groups | To determine social factors central to the understanding of SP and how SP is experienced among stakeholders. To evaluate the effects of SP on patients’ health- service use. | - QuaL: stakeholders’ perceptions of social (dis)connection, and the potential of SP to address it. - QuanT: service use, number of group memberships, community belonging, loneliness (ULS-8). |
| Wallace C, et al (2020) (86) | UK | Research (published paper) | Mixed Methods Study | ﻿A group of (n=18) geographically spread link workers across Wales and 85 conference participants with SP components in their work role | ﻿SP schemes involving assessment and support by a link worker | ﻿To develop an education and training needs conceptual framework for SP in Wales | - ﻿QuanT: Sociodemographic characteristics, statements in response to agreed focus prompts - QuaL: prioritisation of learning needs, identification of appropriate timelines of training delivery |
| Frostick C, et al (2019) (87) | UK | Research (published paper) | Qualitative Study | Link Workers (n=13) actively working in one of three London-based SP schemes | Three SP schemes involving assessment and support by link workers | To identify the training, skills and experience that link workers working with patients with long-term conditions require to carry out their role | - Link workers’ prior expectations, training and experience, as well as perceived challenges |
| Mercer SW et al (2019) (88) | UK | Research (published paper) | Quasi-experimental cluster-randomized controlled trial | Adult patients (n=288) referred to SP in 7 intervention practices, compared with a random sample of adult patients (n= 612) from 8 comparison practices | Referral to a link worker for assessment of patient’s most pressing problems, and ongoing supported referral to  local community resources | To assess the effect of a primary care–based community links practitioner intervention on patients’ quality of life and well-being | - Primary outcome: health-related quality of life (EQ-5D-5L) - Secondary outcomes: well-being (ICECAP-A), depression (HADS-D), anxiety (HADS-A), and self-reported exercise. |
| Payne K, et al (2020) (89) | UK | Research (published paper) | Qualitative Study | Adults (n=17) involved in socially prescribed activities delivered in an urban locality | Initial assessment by a link worker and onwards referral to relevant community groups or in-house support services | To explore the ways by  which SP may be beneficial to individuals undertaking socially prescribed activities | - Participants’ recalled and narrated accounts of their experience participating in socially prescribed activities |

REFERENCES

1. Rempel ES, Wilson EN, Durrant H, Barnett J. Preparing the prescription: a review of the aim and measurement of social referral programmes. BMJ Open [Internet]. 2017 Oct 12 [cited 2018 Jun 12];7(10):e017734. Available from: file:///Users/sarakalde/Library/Application Support/Mendeley Desktop/Downloaded/Rempel et al. - 2017 - Preparing the prescription a review of the aim and measurement of social referral programmes(2).pdf

2. Woodall J, Trigwell J, Bunyan A-MM, Raine G, Eaton V, Davis J, et al. Understanding the effectiveness and mechanisms of a social prescribing service: a mixed method analysis. BMC Heal Serv Res [Internet]. 2018;18(1):604. Available from: http://dx.doi.org/10.1186/s12913-018-3437-7

3. Darnton P, Liles A, Sladen J, Benson T, Lawford N. Independent evaluation of the North East Hampshire and Farnham Vanguard. Making Connections service [Internet]. North East Hampshire and Farnham Vanguard evaluation reports. Southampton: Wessex AHSN; 2018. Available from: http://www.northeasthampshireandfarnhamccg.nhs.uk/about-the-ccg/happy-healthy-at-home/achievements

4. Pescheny J, Randhawa G, Pappas Y. Patient uptake and adherence to social prescribing: a qualitative study. BJGP Open [Internet]. 2018 Oct 7;2(3):bjgpopen18X101598. Available from: https://bjgpopen.org/content/bjgpoa/2/3/bjgpopen18X101598.full.pdf

5. Bickerdike L, Booth A, Wilson PM, Farley K, Wright K, Wilson PM. Social prescribing: less rhetoric and more reality. A systematic review of the evidence. BMJ Open [Internet]. 2017 [cited 2018 Jun 2];7(4):e013384. Available from: file:///Users/sarakalde/Library/Application Support/Mendeley Desktop/Downloaded/Bickerdike et al. - 2017 - Social prescribing less rhetoric and more reality. A systematic review of the evidence(2).pdf

6. Loftus AM, McCauley F, McCarron MO. Impact of social prescribing on general practice workload and polypharmacy. Public Health [Internet]. 2017 Jul 1;148:96–101. Available from: file:///Users/sarakalde/Library/Application Support/Mendeley Desktop/Downloaded/Loftus, McCauley, McCarron - 2017 - Impact of social prescribing on general practice workload and polypharmacy.pdf

7. Kilgarriff-Foster A, O’Cathain A. Exploring the components and impact of social prescribing. J Public Ment Health [Internet]. 2015 Sep 21 [cited 2018 Jun 10];14(3):127–34. Available from: http://eprints.whiterose.ac.uk/96464/

8. Chatterjee HJ, Camic PM, Lockyer B, Thomson LJM. Non-clinical community interventions: a systematised review of social prescribing schemes. Arts Health [Internet]. 2018 May 4 [cited 2019 Jun 20];10(2):97–123. Available from: https://www.tandfonline.com/doi/full/10.1080/17533015.2017.1334002

9. Chesterman D, Bray M. Report on some action research in the implementation of social prescription in Crawley. Paths to greater wellbeing: ‘sometimes you have to be in it to get it.’ Action Learn Res Pract [Internet]. 2018;15(2):168–81. Available from: https://www.scopus.com/inward/record.uri?eid=2-s2.0-85047840904&doi=10.1080%2F14767333.2018.1467302&partnerID=40&md5=40379f0bdae94c79daa1775e5d8aa141

10. Dayson C. Evaluating social innovations and their contribution to social value: The benefits of a “blended value” approach. Policy Polit [Internet]. 2017;45(3):395–411. Available from: https://www.scopus.com/inward/record.uri?eid=2-s2.0-85021826832&doi=10.1332%2F030557316X14564838832035&partnerID=40&md5=184467db08d9bcd4ea330c220d3760f9

11. Ferguson K, Hogarth S. Social prescribing in Tower Hamlets: evaluation of borough-wide roll-out [Internet]. London, UK: University Collegue London; 2018. Available from: file:///Users/sarakalde/Library/Application Support/Mendeley Desktop/Downloaded/Ferguson, Hogarth - 2018 - Social prescribing in Tower Hamlets evaluation of borough-wide roll-out.pdf

12. Blickem C, Kennedy A, Vassilev I, Morris R, Brooks H, Jariwala P, et al. Linking people with long-term health conditions to healthy community activities: development of Patient-Led Assessment for Network Support (PLANS). Heal Expect [Internet]. 2013;16(3):e48-59. Available from: http://dx.doi.org/10.1111/hex.12088

13. Elston J, Gradinger F, Asthana S, Lilley-Woolnough C, Wroe S, Harman H, et al. Does a social prescribing ‘holistic’ link-worker for older people with complex, multimorbidity improve well-being and frailty and reduce health and social care use and costs? A 12-month before-and-after evaluation. Prim Health Care Res Dev [Internet]. 2019 Sep 24;20:e135. Available from: https://www.cambridge.org/core/product/identifier/S1463423619000598/type/journal_article

14. Whitelaw S, Thirlwall C, Morrison A, Osborne J, Tattum L, Walker S. Developing and implementing a social prescribing initiative in primary care: insights into the possibility of normalisation and sustainability from a UK case study. Prim Heal Care Res Dev [Internet]. 2017 Mar 8 [cited 2019 Jun 21];18(2):112–21. Available from: http://dx.doi.org/10.1017/S1463423616000219

15. White JM, Cornish F, Kerr S. Front-line perspectives on ‘joined-up’ working relationships: a qualitative study of social prescribing in the west of Scotland. Health Soc Care Community [Internet]. 2017 Jan;25(1):194–203. Available from: http://dx.doi.org/10.1111/hsc.12290

16. Polley M, Bertotti M, Kimberlee R, Pilkington K, Refsum C, Carpenter A. A review of the evidence assessing impact of social prescribing on healthcare demand and cost implications [Internet]. London: University of Westminster; 2017 [cited 2018 Jun 10]. Available from: file:///Users/sarakalde/Library/Application Support/Mendeley Desktop/Downloaded/Polley et al. - 2017 - A review of the evidence assessing impact of social prescribing on healthcare demand and cost implications.pdf

17. Dayson C, Hogarth S. Evaluation of HALE Community Connectors Social Prescribing Service [Internet]. Sheffield : Sheffield Hallam University; 2018. Available from: file:///Users/sarakalde/Library/Application Support/Mendeley Desktop/Downloaded/Dayson, Hogarth - 2018 - Evaluation of HALE Community Connectors Social Prescribing Service 2017.pdf

18. Husk K, Blockley K, Lovell R, Bethel A, Lang I, Byng R, et al. What approaches to social prescribing work, for whom, and in what circumstances? A realist review. Health Soc Care Community [Internet]. 2020 Mar 9;28(2):309–24. Available from: https://onlinelibrary.wiley.com/doi/abs/10.1111/hsc.12839

19. Pescheny JV, Gunn LH, Randhawa G, Pappas Y. The impact of the Luton social prescribing programme on energy expenditure: a quantitative before-and-after study. BMJ Open [Internet]. 2019;9(6):e026862–e026862. Available from: http://dx.doi.org/10.1136/bmjopen-2018-026862

20. Pescheny J V, Randhawa G, Pappas Y. The impact of social prescribing services on service users: a systematic review of the evidence. Eur J Public Health [Internet]. 2020 Aug 1;30(4):664–73. Available from: http://dx.doi.org/10.1093/eurpub/ckz078

21. Carnes D, Sohanpal R, Frostick C, Hull S, Mathur R, Netuveli G, et al. The impact of a social prescribing service on patients in primary care: a mixed methods evaluation. BMC Heal Serv Res [Internet]. 2017 Dec 19 [cited 2018 Jun 4];17(1):835. Available from: http://dx.doi.org/10.1186/s12913-017-2778-y

22. Thomson LJ, Camic PM, Chatterjee HJ. Social prescribing: a review of community referral schemes [Internet]. London: UCL; 2015. Available from: file:///Users/sarakalde/Library/Application Support/Mendeley Desktop/Downloaded/Thomson, Camic, Chatterjee - 2015 - Social prescribing a review of community referral schemes.pdf

23. Moffatt S, Steer M, Lawson S, Penn L, O’Brien N. Link Worker social prescribing to improve health and well-being for people with long-term conditions: qualitative study of service user perceptions. BMJ Open [Internet]. 2017 Jul 16 [cited 2018 Jun 4];7(7):e015203. Available from: http://dx.doi.org/10.1136/bmjopen-2016-015203

24. Wigfield A, Alden S, Erika K. Age UK’s fit for the future ‘Social Prescribing’ extension project: evaluation report. [Internet]. Leeds: University of Leeds; 2015. Available from: file:///Users/sarakalde/Library/Application Support/Mendeley Desktop/Downloaded/Wigfield, Alden, Erika - 2015 - Age UK’s fit for the future ‘Social Prescribing’ extension project evaluation report.pdf

25. Hamilton-West K, Gadsby E, Zaremba N, Jaswal S. Evaluability assessments as an approach to examining social prescribing. Heal Soc Care Community [Internet]. 2019;27(4):1085–94. Available from: http://dx.doi.org/10.1111/hsc.12726

26. Wildman JM, Moffatt S, Steer M, Laing K, Penn L, O’Brien N. Service-users’ perspectives of link worker social prescribing: a qualitative follow-up study. BMC Public Health [Internet]. 2019 Dec 22 [cited 2019 Jun 21];19(1):98. Available from: https://doi.org/10.1186/s12889-018-6349-x

27. Hanlon P, Gray CM, Chng NR, Mercer SW. Does Self-Determination Theory help explain the impact of social prescribing? A qualitative analysis of patients&#039; experiences of the Glasgow &#039;Deep-End&#039; Community Links Worker Intervention. Chronic Illn. 2019;1742395319845427–1742395319845427.

28. Wildman JM, Moffatt S, Penn L, O’Brien N, Steer M, Hill C. Link workers’ perspectives on factors enabling and preventing client engagement with social prescribing. Health Soc Care Community [Internet]. 2019 Jul 14;27(4):991–8. Available from: http://dx.doi.org/10.1111/hsc.12716

29. Swift M. People powered primary care: Learning from Halton. J Integr Care [Internet]. 2017;25(3):162–73. Available from: https://www.scopus.com/inward/record.uri?eid=2-s2.0-85021350070&doi=10.1108%2FJICA-12-2016-0050&partnerID=40&md5=d8743df21cf5af5746b0ed962c9ee9e1

30. R Outcomes. Social prescribing in Wessex: understanding its impact and supporting spread [Internet]. Southampton: Wessex Academic Health Science Network; 2017. Available from: http://wessexahsn.org.uk/projects/222/summary-evaluation-reports-and-papers

31. Beech R, Ong BN, Jones S, Edwards V. Delivering person-centred holistic care for older people. Qual Ageing Older Adults [Internet]. 2017;18(2):157–67. Available from: file:///Users/sarakalde/Library/Application Support/Mendeley Desktop/Downloaded/Beech et al. - 2017 - Delivering person-centred holistic care for older people.pdf

32. Coan J. Social prescribing at the Bromley by Bow Centre: annual report. April 2015 - March 2016. [Internet]. London: Bromley by Bow Centre,; 2016. Available from: file:///Users/sarakalde/Library/Application Support/Mendeley Desktop/Downloaded/Coan - 2016 - Social prescribing at the Bromley by Bow Centre annual report April 2015 - March 2016.pdf

33. Bertotti M et al, City and Hackney Clinical Commissioning Group. Shine 2014 final report. Social Prescribing. Integrating GP and community assets for Health. [Internet]. London: The Health Foundation; 2015 [cited 2018 Jun 8]. Available from: www.health.org.uk

34. Skivington K, Smith M, Chng NR, Mackenzie M, Wyke S, Mercer SW. Delivering a primary care-based social prescribing initiative: a qualitative study of the benefits and challenges. Br J Gen Pr [Internet]. 2018 Jul 1 [cited 2019 Feb 2];68(672):e487–94. Available from: http://dx.doi.org/10.3399/bjgp18X696617

35. Southby K, Gamsu M. Factors affecting general practice collaboration with voluntary and community sector organisations. Heal Soc Care Community [Internet]. 2018;26(3):e360–9. Available from: http://dx.doi.org/10.1111/hsc.12538

36. Heijnders ML, Meijs JJ. “Welzijn op Recept” (Social Prescribing): A helping hand in re-establishing social contacts-an explorative qualitative study. Prim Heal Care Res Dev. 2018;19(3).

37. Centre for Reviews and Dissemination. Evidence to inform the commissioning of social prescribing [Internet]. Evidence briefing. York: University of York; 2015 [cited 2018 Jun 2]. Available from: file:///Users/sarakalde/Library/Application Support/Mendeley Desktop/Downloaded/Unknown - 2015 - Evidence to inform the commissioning of social prescribing.pdf

38. Pescheny JV, Pappas Y, Randhawa G. Facilitators and barriers of implementing and delivering social prescribing services: a systematic review. BMC Health Serv Res [Internet]. 2018 [cited 2018 Jun 12];18(1):86. Available from: https://www.ncbi.nlm.nih.gov/pmc/articles/PMC5803993/pdf/12913_2018_Article_2893.pdf

39. South J, Higgins TJ, Woodall J, White SM, James; W. Can social prescribing provide the missing link? Prim Heal Care Res Dev [Internet]. 2008 Oct 4 [cited 2018 Jun 17];9(4):310–8. Available from: http://www.journals.cambridge.org/abstract_S146342360800087X

40. Bertotti M, Frostick C, Hutt P, Sohanpal R, Carnes D. A realist evaluation of social prescribing: an exploration into the context and mechanisms underpinning a pathway linking primary care with the voluntary sector. Prim Heal Care Res Dev [Internet]. 2018 [cited 2018 Jun 26];19(3):232–45. Available from: http://dx.doi.org/10.1017/S1463423617000706

41. Bragg R, Leck C. Good practice in social prescribing for mental health: the role of nature-based interventions. [Internet]. Natural England Commissioned Report. [York]: Natural England; 2017. Available from: http://publications.naturalengland.org.uk/publication/5134438692814848

42. Bungay H, Clift S. Arts on Prescription: A review of practice in the UK. Perspect Public Health [Internet]. 2010;130(6):277–81. Available from: file:///Users/sarakalde/Library/Application Support/Mendeley Desktop/Downloaded/Bungay, Clift - 2010 - Arts on Prescription A review of practice in the UK.pdf

43. Grant C. A randomised controlled trial and economic evaluation of a referrals facilitator between primary care and the voluntary sector. BMJ [Internet]. 2000 Feb 12;320(7232):419–23. Available from: http://www.bmj.com/cgi/doi/10.1136/bmj.320.7232.419

44. Jensen A. Culture Vitamins – an Arts on Prescription project in Denmark. Perspect Public Health [Internet]. 2019 May 8;139(3):131–6. Available from: http://journals.sagepub.com/doi/10.1177/1757913919836145

45. Jensen A, Stickley T, Torrissen W, Stigmar K. Arts on prescription in Scandinavia: a review of current practice and future possibilities. Perspect Public Health [Internet]. 2017;137(5):268–74. Available from: file:///Volumes/TOSHIBA EXT/BIBLIO MENDELEY/BIBLIO MENDELAY/Jensen et al. - Perspectives in public health - 2017.pdf

46. Maughan DL, Patel A, Parveen T, Braithwaite I, Cook J, Lillywhite R, et al. Primary-care-based social prescribing for mental health: an analysis of financial and environmental sustainability. Prim Heal Care Res Dev [Internet]. 2016;17(2):114–21. Available from: http://dx.doi.org/10.1017/S1463423615000328

47. Redmond M, Sumner RC, Crone DM, Hughes S. Light in dark places: exploring qualitative data from a longitudinal study using creative arts as a form of social prescribing. Arts Heal [Internet]. 2018;1–14. Available from: http://dx.doi.org/10.1080/17533015.2018.1490786

48. Age UK. Social prescribing: A model for partnership working between primary care and the voluntary sector. York: Age UK; 2018.

49. Stickley T, Eades M. Arts on prescription: a qualitative outcomes study. Public Health [Internet]. 2013;127(8):727–34. Available from: http://dx.doi.org/10.1016/j.puhe.2013.05.001

50. Stickley T, Hui A. Social prescribing through arts on prescription in a UK city: Referrers’ perspectives (part 2). Public Health [Internet]. 2012 Jul;126(7):580–6. Available from: https://linkinghub.elsevier.com/retrieve/pii/S0033350612001370

51. Stickley T, Hui A. Social prescribing through arts on prescription in a UK city: Participants’ perspectives (Part 1). Public Health [Internet]. 2012 Jul;126(7):580–6. Available from: http://dx.doi.org/10.1016/j.puhe.2012.04.001

52. Sumner RC, Crone DM, Baker C, Hughes S, Loughren EA, James DVB. Factors associated with attendance, engagement and wellbeing change in an arts on prescription intervention. J public Heal [Internet]. 2019 Apr 8; Available from: http://dx.doi.org/10.1093/pubmed/fdz032

53. Thomson LJ, Lockyer B, Camic PM, Chatterjee HJ. Effects of a museum-based social prescription intervention on quantitative measures of psychological wellbeing in older adults. Perspect Public Heal [Internet]. 2018;138(1):28–38. Available from: http://dx.doi.org/10.1177/1757913917737563

54. Creative Alternatives. Arts on prescription in Sefton. Programme Report December 2009 [Internet]. [Netherton]: Creative Alternatives; 2009. Available from: http://www.artsforhealth.org/resources/CA Report 2009.pdf

55. Dayson C, Bashir N, Pearson S. From dependence to independence: emerging lessons from the Rotherham Social Prescribing Pilot [Internet]. Sheffield: Centre for Regional Economic and Social Research. Sheffield Hallam University; 2013 [cited 2018 Jun 8]. Available from: http://www.instituteofhealthequity.org/file-manager/PDFs/from-dependence-to-independence-emerging-lessons-from-the-rotherham-social-prescribing-pilot-summary-report.pdf

56. Faulkner M. Supporting the psychosocial needs of patients in general practice: the role of a voluntary referral service. Patient Educ Couns [Internet]. 2004 Jan 1 [cited 2019 Jun 21];52(1):41–6. Available from: https://www.sciencedirect.com/science/article/pii/S0738399102002471?via%3Dihub

57. Roessler KK. A corrective emotional experience - or just a bit of exercise? The relevance of interpersonal learning in Exercise on prescription. Scand J Psychol [Internet]. 2011 Aug;52(4):354–60. Available from: http://doi.wiley.com/10.1111/j.

58. Baines A. Rugby Social Prescribing Project ConnectWELL. Harnessing community capacity to improve health and wellbeing. Vol. 151. Coventry: Roundberry Projects; 2015.

59. Edmunds J, Ntoumanis N, Duda JL. Adherence and well-being in overweight and obese patients referred to an exercise on prescription scheme: A self-determination theory perspective. Psychol Sport Exerc [Internet]. 2007 Sep;8(5):722–40. Available from: https://linkinghub.elsevier.com/retrieve/pii/S146902920600077X

60. Jones F, Harris P, Waller H, Coggins A. Adherence to an exercise prescription scheme: The role of expectations, self-efficacy, stage of change and psychological well-being. Br J Health Psychol [Internet]. 2005 Sep;10(3):359–78. Available from: http://doi.wiley.com/10.1348/135910704X24798

61. Murphy SM, Edwards RT, Williams N, Raisanen L, Moore G, Linck P, et al. An evaluation of the effectiveness and cost effectiveness of the National Exercise Referral Scheme in Wales, UK: a randomised controlled trial of a public health policy initiative. J Epidemiol Community Health [Internet]. 2012 Aug;66(8):745–53. Available from: http://jech.bmj.com/lookup/doi/10.1136/jech-2011-200689

62. Rahman RJ, Thogersen-Ntoumani C, Thatcher J, Doust J. Changes in need satisfaction and motivation orientation as predictors of psychological and behavioural outcomes in exercise referral. Psychol Health [Internet]. 2011 Nov;26(11):1521–39. Available from: http://www.tandfonline.com/doi/abs/10.1080/08870446.2010.538849

63. Morton KL, Biddle SJH, Beauchamp MR. Changes in self-determination during an exercise referral scheme. Public Health [Internet]. 2008 Nov;122(11):1257–60. Available from: https://linkinghub.elsevier.com/retrieve/pii/S003335060700371X

64. Kimberlee RH. Developing a Social Prescribing approach for Bristol [Internet]. Bristol: University of the West of England; 2013. Available from: http://eprints.uwe.ac.uk/23221/1/Social Prescribing Report-final.pdf

65. Public Health England. Effectiveness of social prescribing - An evidence synthesis [Internet]. [London]: Public Health England; 2019 [cited 2019 Sep 29]. Available from: www.facebook.com/PublicHealthEngland

66. Duda JL, Williams GC, Ntoumanis N, Daley A, Eves FF, Mutrie N, et al. Effects of a standard provision versus an autonomy supportive exercise referral programme on physical activity, quality of life and well-being indicators: a cluster randomised controlled trial. Int J Behav Nutr Phys Act [Internet]. 2014;11(1):10. Available from: http://ijbnpa.biomedcentral.com/articles/10.1186/1479-5868-11-10

67. Kok M, Solomon-Moore E, Greaves C, Smith J, Kimberlee R, Jones M. Evaluation of Living Well, Taking Control: a community-based diabetes prevention and management programme [Internet]. [Bristol]: University of The West of England; 2016. Available from: http://eprints.uwe.ac.uk/30234/13/LWTC Evaluation Report_finalISBN.pdf

68. Mercer S. Evaluation of the Glasgow “Deep End” Links Worker Programme [Internet]. [Edinburgh]: NHS Health Scotland; 2017. Available from: http://www.healthscotland.com/uploads/documents/29438-1. Evaluation of the Glasgow “Deep End” Links Worker Programme - May 2017 - English.pdf

69. Mills H, Crone D, James DVB, Johnston LH. Exploring the Perceptions of Success in an Exercise Referral Scheme: a mixed method investigation. Eval Rev [Internet]. 2012 Dec 2;36(6):407–29. Available from: http://journals.sagepub.com/doi/10.1177/0193841X12474452

70. Dayson C, Bashir N, Bennett E, Sanderson E. The Rotherham Social Prescribing Service for People with Long-Term Health Conditions: Annual Evaluation Report. Sheffield: Sheffield Hallam University. Centre for Regional Economic and Social Research; 2016.

71. Kimberlee RH. Gloucestershire Clinical Commissioning Group’s Social Prescribing Service: Evaluation Report [Internet]. Bristol: University of the West of England; 2016. Available from: http://www.periphery.co.uk/joyn

72. Rouse PC, Ntoumanis N, Duda JL, Jolly K, Williams GC. In the beginning: Role of autonomy support on the motivation, mental health and intentions of participants entering an exercise referral scheme. Psychol Health [Internet]. 2011 Jun;26(6):729–49. Available from: http://www.tandfonline.com/doi/abs/10.1080/08870446.2010.492454

73. Brandling J, House W. Investigation into the feasibility of a social prescribing service in primary care: a pilot project [Internet]. Bath: University of Bath; 2007 [cited 2018 Jun 2]. Available from: http://opus.bath.ac.uk/22487/1/Brandling_SocialPrescribingFeasabilityReport.pdf

74. Wormald H, Waters H, Sleap M, Ingle L. Participants’ perceptions of a lifestyle approach to promoting physical activity: targeting deprived communities in Kingston-Upon-Hull. BMC Public Health [Internet]. 2006 Dec 4;6(1):202. Available from: https://bmcpublichealth.biomedcentral.com/articles/10.1186/1471-2458-6-202

75. NHS Leeds West CCG. Patient Empowerment Project Final Evaluation Report [Internet]. [Leeds]: NHS Leeds West Clinical Commissioning Group; 2016. Available from: https://www.leedsccg.nhs.uk/content/uploads/2018/05/Patient-Empowerment-Project-Leeds-West-Final-Report.pdf

76. Mistry B, Phillips L, Simpson J. Prescription Plus Crawley. The Case for Project Expansion. Crawley; 2017.

77. Jones M, Kimberlee R, Deave T. South West Well-being Programme: Final Evaluation Report. [Internet]. Bristol; 2009. Available from: http://hsc.uwe.ac.uk/net/research/Data/Sites/1/UWE-SWWB-Report-WebVersion.pdf

78. Tava’E N, Nosa V. The green prescription programme and the experiences of Pacific women in Auckland. J Prim Health Care. 2012;4(4):313–9.

79. Kimberlee R. What is social prescribing? Adv Soc Sci Res J [Internet]. 2015 Jan 25 [cited 2018 Jun 10];2(1). Available from: http://scholarpublishing.org/index.php/assrj/article/view/808

80. ERS Research and Consultancy. Newcastle Social Prescribing Project. Final Report. [Newcastle upon Tyre]: ERS Research and Consultancy; 2013.

81. Grayer J, Cape J, Orpwood L, Leibowitz J, Buszewicz M. Facilitating access to voluntary and community services for patients with psychosocial problems: a before-after evaluation. BMC Fam Pract [Internet]. 2008 Dec 7;9(1):27. Available from: http://bmcfampract.biomedcentral.com/articles/10.1186/1471-2296-9-27

82. Aggar C, Thomas T, Gordon C, Bloomfield J, Baker J. Social Prescribing for Individuals Living with Mental Illness in an Australian Community Setting: A Pilot Study. Community Ment Health J [Internet]. 2020 May 13; Available from: http://link.springer.com/10.1007/s10597-020-00631-6

83. Beardmore A. Working in social prescribing services: a qualitative study. J Health Organ Manag [Internet]. 2019 Nov 4;34(1):40–52. Available from: https://www.emerald.com/insight/content/doi/10.1108/JHOM-02-2019-0050/full/html

84. Tierney S, Wong G, Roberts N, Boylan A-M, Park S, Abrams R, et al. Supporting social prescribing in primary care by linking people to local assets: a realist review. BMC Med [Internet]. 2020 Dec 13;18(1):49. Available from: https://bmcmedicine.biomedcentral.com/articles/10.1186/s12916-020-1510-7

85. Kellezi B, Wakefield JRH, Stevenson C, McNamara N, Mair E, Bowe M, et al. The social cure of social prescribing: a mixed-methods study on the benefits of social connectedness on quality and effectiveness of care provision. BMJ Open [Internet]. 2019 Nov 14;9(11):e033137. Available from: http://bmjopen.bmj.com/lookup/doi/10.1136/bmjopen-2019-033137

86. Wallace C, Elliott M, Thomas S, Davies-McIntosh E, Beese S, Roberts G, et al. Using consensus methods to develop a Social Prescribing Learning Needs Framework for practitioners in Wales. Perspect Public Health [Internet]. 2020 Jan 28;175791391989794. Available from: http://journals.sagepub.com/doi/10.1177/1757913919897946

87. Frostick C, Bertotti M. The frontline of social prescribing – How do we ensure Link Workers can work safely and effectively within primary care? Chronic Illn [Internet]. 2019 Oct 17;174239531988206. Available from: http://journals.sagepub.com/doi/10.1177/1742395319882068

88. Mercer SW, Fitzpatrick B, Grant L, Chng NR, McConnachie A, Bakhshi A, et al. Effectiveness of Community-Links Practitioners in Areas of High Socioeconomic Deprivation. Ann Fam Med [Internet]. 2019 Nov 11;17(6):518–25. Available from: http://www.annfammed.org/lookup/doi/10.1370/afm.2429

89. Payne K, Walton E, Burton C. Steps to benefit from social prescription: a qualitative interview study. Br J Gen Pract [Internet]. 2020 Jan;70(690):e36–44. Available from: http://bjgp.org/lookup/doi/10.3399/bjgp19X706865
